# AI assessment changes human behavior

**DOI:** 10.1073/pnas.2425439122

**Published:** 2025-06-20

**Authors:** Jonas Goergen, Emanuel de Bellis, Anne-Kathrin Klesse

**Affiliations:** ^a^Institute of Behavioral Science and Technology, University of St.Gallen, St.Gallen 9000, Switzerland; ^b^Rotterdam School of Management, Erasmus University Rotterdam, Rotterdam 3062 PA, The Netherlands

**Keywords:** artificial intelligence, algorithms, AI assessment, behavioral changes, lay beliefs

## Abstract

AI is increasingly replacing human decision-makers across domains. AI-based tools have become particularly common in assessment decisions, such as when recruiting employees or admitting students. Calls for transparency and new legislation require organizations to disclose the use of AI assessment tools, thus making people under assessment aware of its use. We investigate whether this shift from human to AI assessment affects people’s behavior during the assessment. We propose that people emphasize their analytical characteristics and downplay their intuitive and emotional ones under AI (vs. human) assessment, a phenomenon we label “the AI assessment effect.” Twelve studies (eight in text and four in the Supporting Information; *N* = 13,342) document the AI assessment effect and its underlying mechanism: the lay belief that AI prioritizes analytical characteristics in its assessment. Whereas prior work has studied perceptions of AI assessment tools and their productivity gains, the current research demonstrates systematic behavioral changes because of AI assessment. The findings offer theoretical contributions to the psychology of AI and practical insights for organizations using AI assessment.

## Significance

The shift from human to AI assessment raises an important question: do people behave differently when being assessed by AI? If so, this might have significant consequences for both people under assessment and the organizations conducting the assessment. Focusing on candidate selection decisions as a key assessment domain, the current research shows that AI assessment leads people to present themselves as more analytical because they believe that AI particularly values analytical characteristics. This behavioral shift could fundamentally alter who gets selected for positions, potentially undermining the validity of assessment processes. Overall, this work reveals how people change their behavior under AI assessment, offering insights for organizations and policymakers navigating the integration of AI in high-stakes decisions.

Should a student be admitted to an educational program? Should a prospective employee be given a job offer? Although such decisions used to be made solely by humans, the rapid development and increasing availability of AI and algorithms is leading to a fundamental shift in assessment procedures. Organizations are now relying on AI-based assessment tools (“AI assessment” hereinafter) because they promise gains in efficiency and productivity (1–4). Particularly in the context of human resource management, AI assessment has become widespread (5, 6).

In this research, we employ a psychological lens and suggest that informing people about AI (vs. human) assessment leads to changes in behavior during the assessment so that people emphasize their analytical characteristics. We refer to this phenomenon as “the AI assessment effect.” We argue that this effect stems from the lay belief that AI prioritizes analytical capabilities over emotional skills and intuition, which would make analytical behavior more likely to be rewarded in the assessment.

The prediction that AI assessment triggers changes in behavior broadly aligns with anecdotal evidence and qualitative research suggesting that people adapt to the new assessment paradigm. That is, people report changing their behavior to “pacify” the assessor, learn in classes how to alter their conduct to “get past the AI hiring filter,” and edit their job application material to “beat the bots” (7–9).

Our prediction receives initial empirical support from an exploratory study that we conducted with Equalture, an employment software company offering game-based assessment procedures for employee recruitment. Specifically, we administered a short survey to candidates who participated in the company’s game-based assessment. We asked 1,421 applicants who they believed made the assessment, on a continuum with endpoints 1 (exclusively made by a human) and 7 (exclusively made by an algorithm), and whether they accordingly adapted their behavior during the assessment, with endpoints 1 (no adjustment at all) and 7 (strong adjustment). Although the company did not mention the form of the assessor (algorithm vs. human), candidates suspected that the assessment was predominantly algorithm-based (*Mdn* = 5, *M* = 4.69; *t*(1,420) = 17.85, *P* < 0.001). Importantly, the more applicants suspected algorithm involvement in the assessment, the stronger the self-reported adjustment of their behavior (*r*(1,407) = 0.12, *P* < 0.001, 95% CI = [0.06, 0.18]; for details, see *SI Appendix*, section A).

Why is it important to understand whether and how being informed of AI assessment can alter people’s behavior? First, people are becoming more aware of AI assessment as a result of new legislation (e.g., the European Union’s AI Act, Illinois’ Artificial Intelligence Video Interview Act, and New York City’s Local Law 144), requiring organizations to be transparent about the use of AI assessment (10–12). Second, assessment procedures are intended to evaluate people’s authentic qualities and performance to make informed decisions. Yet, if people strategically change the way they behave during the assessment because they believe their assessor is an AI, assessment decisions may be distorted, with potentially dire consequences. For example, organizations might hire people who do not match the job profile, resulting in dissatisfaction, inefficiencies, and diminished employee well-being. Third, the lay belief that AI prioritizes analytical characteristics might be inaccurate, as AI is building capabilities to detect, analyze, interpret, and mimic emotions and intuition (13–15). Thus, behavioral changes intended to improve assessment outcomes might be misinformed.

Prior work on AI in assessment decisions—conducted across different disciplines—has predominantly investigated the objective outcomes of AI assessment for 1) organizations (e.g., increased efficiency) and 2) people under assessment (e.g., decreased discrimination), as well as people’s perceptions of and attitude toward it (e.g., trust, fairness); for an overview, see *SI Appendix*, section B. Our research diverges from prior work by examining the possibility that disclosing AI assessment results in systematic changes in the behavior of people under assessment.

## Theory

People’s tendency to control and manage the impressions they make on others is a bedrock in psychology and the basis of impression management and self-presentation theory (16, 17). The tendency to manage the image conveyed to others is particularly pronounced in assessment situations because the assessment has direct consequences (e.g., being selected or rejected; 18). When people have (or think they have) a clear idea of the assessor’s standards, they may strategically adapt their behavior to match what they believe the assessor will evaluate positively. Such behavior has been documented by research on the phenomena of accountability (18) and faking (19). In particular, candidates may misrepresent themselves during an assessment to garner favorable evaluations from the assessor (20, 21). Though common, such strategic shifts in self-presentation might harm the validity of assessment (measures) and are negatively related to job performance (21–23). They are most likely to occur when two conditions are met: First, people must know (or presume to know) what behavior is assessed, and second, they must be able to estimate (or believe they can estimate) what kind of behavior will be most favorably evaluated by the assessor (24).

Regarding AI and algorithms, existing research has shown that people possess lay beliefs about how AI works and where it excels (or falls short). Such lay beliefs, describing people’s common-sense explanations for the world around them (25)—whether right or wrong—drive their reactions to and interactions with AI (26–29). When informed that they will undergo AI assessment, without being offered specific information about the AI, people are thus likely to rely on their lay beliefs regarding the type of behavior AI will assess most favorably. Prior work suggests that people perceive AI as inherently lacking emotional and intuitive capabilities, but possessing analytical and rational ones (30, 31). Thus, AI-based decision-making generally, and assessment processes specifically, may be construed as purely analytical and data-driven, relying on objective, quantifiable, and rule-based analysis rather than subjective, intuitive, and qualitative reasoning (32–34). Consequently, people might expect AI to define its assessment criteria and preferences to prioritize analytical traits while neglecting emotional, subjective, and qualitative dimensions. This assumption is in line with prior work suggesting that people perceive AI and algorithms as relying on facts, logic, and rationality rather than emotions and intuition when offering recommendations (30, 31). Relatedly, prior work suggests that AI assessment is viewed as reducing individuals to quantifiable, analytical traits (35).

Building on this, we suggest that people may believe that AI prioritizes analytical thinking or traits in its assessment of people, a belief that we term “the analytical priority lay belief.” We further posit that this belief may prompt a strategic change in behavior by the people under AI assessment, such that they adjust their self-presentation to emphasize their analytical characteristics and downplay their intuition. We focus on the analytical–intuitive dimension for several reasons. First, it corresponds to lay beliefs about AI (32). Second, it reflects well-established dual-process accounts underlying human reasoning, judgment, and social cognition (36). Third, its manifestations in personality, skills, and behavior are fundamental decision criteria relevant to organizations using AI assessment (37).

We test the AI assessment effect across eight studies in the main text and four additional studies in the *SI Appendix* (*N* = 13,342; for an overview, see *SI Appendix*, section C). To ensure the generalizability and robustness of the AI assessment effect, we vary the design and experimental paradigms (between- and within-subjects studies, vignette studies, incentive-aligned paradigms, and real applications), the dependent variable (self-reports and behavior), and the context (job recruiting, college admissions, and research pool applications). To investigate whether the AI assessment effect generalizes to actual, more noisy candidate selection procedures, we conducted one study with job applicants on a recruiting website. All studies were preregistered. We provide access to all data and analysis code via *SI Appendix* and OSF (38).

## Results

### Pilot Study.

The Pilot Study (https://aspredicted.org/6mn9-c2jy.pdf) tested the AI assessment effect in the field. For this purpose, we created two open job descriptions for an event planner (for an actual event) on the freelancing platform Upwork. The two positions were identical in the described task and profile but differed in the information that was provided regarding how applications are assessed. In the AI (human) condition, we specified that the application would be assessed by an AI (a human) (*SI Appendix*, section D). To prevent prospective candidates from seeing both positions, we invited freelancers to apply for either one or the other. Invited freelancers could apply for the job by answering two prescreening questions. As our key variable, we asked: “Please describe yourself to convince the artificial intelligence (AI tool) [recruiter] assessing you. Are you more of an analytical or intuitive person? Write down a number between 1 and 7, with 1 = strongly intuitive and 7 = strongly analytical.” We used this score to evaluate whether freelancers described themselves as being a more analytical person under AI assessment.

We received a total of 278 applications (60% female, 40% male). In line with our preregistered hypothesis, freelancers invited to the AI-assessed position tended to report more analytical self-descriptions than those invited to the human-assessed position (*M*_AI_ = 4.41 vs. *M*_human_ = 4.02; *t*(274) = 2.04, *P* = 0.042, *d* = 0.25, 95% CI = [0.01, 0.49]); the effect was robust when controlling for applicants’ gender. The findings provide preliminary evidence for the AI assessment effect in the field. Yet we acknowledge the noisy nature of online platform testing (39)—random assignment to conditions could not be guaranteed and extraneous factors may have contributed to the effect.

### Study 1.

In Study 1 (https://aspredicted.org/kxc8-48ng.pdf), we aimed to provide causal evidence for the AI assessment effect in a controlled setting. To do so, we told participants that their performance on a task would be assessed by either an AI or a human, and then asked everyone to report the extent to which they would approach the task in an analytical versus intuitive manner. The second objective of this study was to explore whether the AI assessment effect stems from participants’ lay belief that AI prioritizes analytical characteristics in its assessment.

Five hundred thirteen participants (*M*_age_ = 37.4 y; 60% female, 38% male) took part in a two-cell (AI vs. human) between-subjects experimental design. Participants imagined applying for admission to a fictitious college (Horizon University). They were randomly assigned to either the AI or human assessment. Depending on condition, they were informed that either an AI or a person would assess them in how they approach a task. We purposefully did not reveal details about the task to test our predictions in the absence of information that might cue analytical or intuitive approaches as more effective (40).

We then measured participants’ self-reported approach to the task with an abbreviated version of the situational thinking style measure (41). Specifically, five items gauged an analytical task approach (e.g., “I would figure things out logically”; α = 0.87), and five items captured a more intuitive task approach (e.g., “I would go by what felt good to me”; α = 0.93; 7-point agreement Likert scales; for full measures, see *SI Appendix*, section E). Following the original measure, we analyzed both dimensions separately. Moreover, we assessed participants’ analytical priority lay belief for the AI and human via three items (e.g., “The AI [human] places an emphasis on analytical attributes rather than intuitive ones”; 7-point agreement Likert scales; α = 0.93).

#### Task approach.

**Fig. 1.**
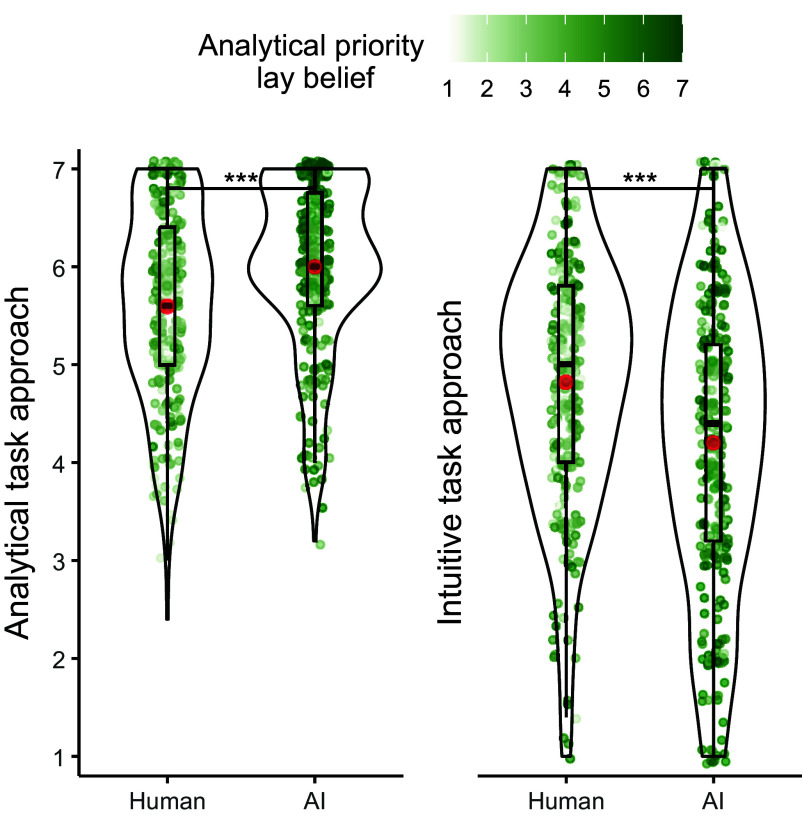
Differences in analytical and intuitive task approach in Study 1. Participants reported approaching the task more analytical and less intuitive under AI (vs. human) assessment. The green dots are individual observations color-coded for analytical priority lay beliefs. The red dots visualize the means. The bold horizontal lines visualize medians. Upper and lower box plot bounds represent third and first quartiles, respectively. The whiskers extend to maximum 1.5 × interquartile range from the quartile boundary. The violin plots are trimmed at the maximum and minimum observed value per condition. Violin plot width corresponds to the frequency of observations at any value on the y-axis. N = 513. ***P < 0.001.

Under AI (vs. human) assessment, participants reported approaching the task more analytically (*M*_AI_ = 6.00 vs. *M*_human_ = 5.59; *t*(511) = 4.95, *P* < 0.001, *d* = 0.44, 95% CI = [0.26, 0.62]) and less intuitively (*M*_AI_ = 4.20 vs. *M*_human_ = 4.82; *t*(511) = −4.93, *P* < 0.001, *d* = −0.43, 95% CI = [−0.60, −0.25]; Fig. 1).

#### Analytical priority lay belief.

Participants perceived the AI to prioritize the analytical dimension more so than the human (*M*_AI_ = 5.79 vs. *M*_human_ = 4.19; *t*(511) = 13.98, *P* < 0.001, *d* = 1.23, 95% CI = [1.02, 1.46]). We next ran two separate mediation analyses to examine whether this lay belief mediated the AI assessment effect. We specified the assessor as the independent variable (0 = human, 1 = AI), analytical priority lay belief as the mediator, and analytical and intuitive task approaches, respectively, as the dependent variables (42). We found the proposed mediation for both analytical and intuitive task approaches in the form of significant indirect effects (*b*_analytical_ = 0.30, 95% CI = [0.17, 0.44]; *b*_intuitive_ = −0.35, 95% CI = [−0.52, −0.18]). More specifically, AI assessment was associated with greater analytical priority lay beliefs (*b* = 1.61, *P* < 0.001), which in turn resulted in increased analytical task approaches (*b* = 0.18, *P* < 0.001) and decreased intuitive task approaches (*b* = −0.22, *P* < 0.001).

To test the robustness and generalizability of the AI assessment effect, we conducted four replication studies (*N* = 1,424), slightly varying the context (college admission, job application), design (between and within subjects), terminology (“AI” vs. “algorithm”), and dependent variables of self-reported task approach (analytical vs. intuitive, reliance on affect versus cognition) (43). All studies offer consistent evidence for the AI assessment effect (*d* ranges from 0.22 to 0.72; see *SI Appendix*, sections F–I).

### Study 2.

Study 2 (https://aspredicted.org/c2vz-3k2m.pdf) tested the robustness of the AI assessment effect in a two-stage study with a representative US sample (age, gender, and ethnicity). One question pertains to how homogeneous the effect is and whether it depends on salient individual differences. We used an “exploration of moderators under uncertainty” approach (44) and grouped the moderators into three categories: individual differences related to 1) AI receptiveness, 2) the job application context, and 3) personality more generally.

Following good practice, we measured all moderators in a stage 1 survey temporally separated from testing the AI assessment effect (45). Participants (*N* = 1,486; *M*_age_ = 45.1 y; 50% female, 48% male) responded to measures related to AI receptiveness [including objective AI literacy (46), attitudes toward AI (47) (α = 0.94), subjective understanding of AI (48), and familiarity with AI, approximated by use of AI in daily life (46); for full measures, see *SI Appendix*, section J], the application context (including experience with applications and anxiety about one’s future employment outlook; self-developed items based on ref. 49; α = 0.92), and personality [including Big Five Inventory 10 (50) and fear of negative evaluation (51); α = 0.96]. At the end of the survey, participants responded to demographic questions (age, gender, income, education, and occupation).

We launched the stage 2 survey four days later, and 1,166 participants (*M_age_* = 45.7 y; 51% female, 48% male) returned to participate in a two-cell (AI vs. human) between-subjects design similar to Study 1 but in the context of applying for a job. As the dependent variable, participants reported how they would describe their own approach to tasks on a one-dimensional three-item scale that was anchored at a more analytical pole and a more intuitive pole (43, 52) (e.g., “When approaching tasks, I would rely on…” 1 = my thinking vs. 7 = my feeling; α = 0.88).

#### Analytical task approach.

Participants presented themselves more analytically under AI assessment, as evidenced by the lower mean (*M*_AI_ = 2.54 vs. *M*_human_ = 3.00; *t*(1,162) = −5.07, *P* < 0.001, *d* = −0.30, 95% CI = [−0.41, −0.18]).

#### Moderations.

Our analysis strategy was similar across moderators. We ran an ANOVA model specifying task approach as the dependent variable and AI versus human assessment as the first factor. The second factor was the respective individual difference variable. Only the interaction effect with age reached significance (*b* = 0.01, SE = 0.01, *t*(1,160) = 2.17, *P* = 0.030). A Johnson–Neyman (JN) floodlight analysis (53) indicated that the AI assessment effect is more pronounced among younger cohorts (JN point = 62 y; see *SI Appendix*, section J).

Moreover, we found a marginally significant interaction for fear of negative evaluation (*b* = −0.12, SE = 0.06, *t*(1,160) = −1.84, *P* = 0.066), agreeableness (*b* = 0.13, SE = 0.07, *t*(1,160) = 1.91, *P* = 0.056), and occupation (*SI Appendix*, section J), which we report here for the sake of completeness. The patterns suggest that the AI assessment effect emerges for people with higher fears of negative evaluation (JN point = 2.17) and lower agreeableness (JN point = 6.41), while it is stronger (weaker) among unemployed (retired) participants compared with employed participants. Importantly, the AI assessment effect is robust when including all covariates in the model (*b* = −0.48, SE = 0.09, *t*(1,136) = −5.04, *P* < 0.001).

### Study 3.

Study 3 (https://aspredicted.org/kns4-dz6k.pdf) employed a two-stage randomized mixed design in which we manipulated form of the assessor across the two stages, allowing us to examine both between- and within-subjects changes in self-presentation as a consequence of AI versus human assessment. For exploratory purposes, this study included a no-assessment condition in stage 1. Here, participants were simply asked to describe themselves, with no mention of assessment, to get an approximation of “the ground truth” (i.e., how participants generally approach tasks).

A total of 1,485 participants (*M*_age_ = 39.7 y; 58% female, 40% male) were randomly assigned to an AI, human, or no-assessment condition in stage 1. Whereas the two assessment conditions followed the paradigm employed in prior studies, participants in the no-assessment condition were asked to describe their approach to a task in general, with no mention of assessment. The key dependent variable was Study 1’s 5-item measure of analytical task approach (α = 0.88). This focal measure was presented among work-related filler items (e.g., communication preferences) and followed by additional measures of self-presentation, namely, creativity, risk-taking, effort investment, and ethical and social considerations in approaching tasks included for exploratory purposes (*SI Appendix*, section K).

A minimum of 4 d after stage 1, we launched the stage 2 survey, to which 1,138 participants (*M*_age_ = 40.6 y; 58% female, 41% male) returned. Regardless of their initial condition, participants were randomly assigned to either an AI or a human assessment condition. As part of the measurement battery of stage 2, participants repeated the same 5-item analytical measure from stage 1 (α = 0.89), presented alongside filler items and followed by the exploratory measures.

Participants could encounter one of six possible sequences in stages 1 and 2 (AI → AI, AI → human, human → AI, human → human, no assessment → AI, no assessment → human). This design enabled us to gauge 1) a between-subjects comparison at stage 1, for which we predicted a more analytical task approach in the AI (vs. human) condition, and 2) within-subjects changes between stage 1 and stage 2, for which we predicted a stronger shift in task approaches when participants switched from one assessor (e.g., human) to another (e.g., AI) than when being assessed by the same assessor twice. Finally, 3) the design enabled us to compare the two assessments with the no-assessment baseline, allowing us to explore which assessment form is closer to how participants describe themselves when not being assessed.

#### Between-subjects differences in stage 1.

**Fig. 2.**
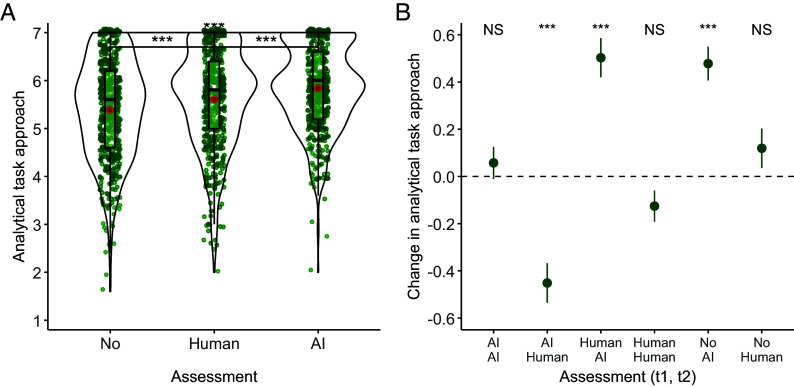
Differences in analytical task approach in Study 3 between subjects (*A*) and within subjects over the two stages (*B*). Participants reported approaching the task more analytical under AI assessment (vs. no assessment and human assessment) (*A*). Participants also reported approaching the task more (less) analytically after switching from human (AI) to AI (human) assessment and from no assessment to AI assessment (*B*). The specifics of panel *A* correspond to Fig. 1. In panel *B*, green dots describe means with SEM for the six possible combinations of condition assignment across stages 1 and 2. ***P < 0.001, NS *P* > 0.05.

We focus our analysis on participants who completed both stages. In stage 1, we found significant differences in self-reported task approaches between conditions (*F*(2, 1,135) = 19.27, *P* < 0.001, *η*_p_^2^ = 0.03; Fig. 2*A*). Under AI assessment, task approaches were significantly more analytical (*M* = 5.85) than under human assessment (*M* = 5.60; *t*(1,135) = 3.49, *P* < 0.001, *d* = 0.26, 95% CI = [0.12, 0.39]), replicating the results of prior studies.

#### Within-subjects differences from stage 1 to stage 2.

We first investigated whether there were stronger shifts in task approaches when a participant went from human to AI assessment (or vice versa), compared with seeing the same assessor twice. A two-way ANOVA on the absolute difference in analytical task approaches between stages, with assessment alteration across stages (same vs. different) and order (human first vs. AI first) as between-subjects factors, showed a significant main effect of assessment alteration (*F*(1, 736) = 11.69, *P* < 0.001, *η*_p_^2^ = 0.03). We found neither a significant main effect of order nor an interaction effect. As predicted, AI assessment led to more analytical task approaches (*M* = 6.00) relative to human assessment (*M* = 5.52; *t*(389) = 8.10, *P* < 0.001, *d* = 0.41, 95% CI = [0.31, 0.50]; Fig. 2*B*).

#### Exploratory analyses.

Compared with no assessment, AI assessment starkly increased analytical task approaches for both the between-subjects comparison at stage 1 (*M*_AI_ = 5.85 vs. *M*_no_ = 5.42; *t*(1,135) = 6.20, *P* < 0.001, *d* = 0.45, 95% CI = [0.31, 0.60]) and the within-subjects comparison over the two stages (*M*_AI_ = 5.87 vs. *M*_no_ = 5.39; *t*(204) = 6.71, *P* < 0.001, *d* = 0.48, 95% CI = [0.33, 0.61]). There was also a smaller upward adjustment in analytical task approaches under human assessment in stage 1 (*M*_human_ = 5.60 vs. *M*_no_ = 5.42; *t*(1,135) = 2.63, *P* = 0.009, *d* = 0.19, 95% CI = [0.04, 0.35]).

Given that AI (vs. human) assessment prompts participants to present themselves differently, it may result in differences in terms of who will be selected (vs. rejected) for a specific job. For instance, an organization might define the average analytical task approach of a suitable candidate on a 7-point scale to be above the threshold of 6. Employing this self-defined threshold allows us to explore whether there are differences in a candidate’s likelihood of being selected, depending on whether they were assessed by AI or a human. For the exemplary threshold of 6, 27% of all participants would be selected for the job only if assessed by AI but not if assessed by a human. For the sake of robustness, we repeated this analysis for various thresholds as outlined in our preregistration, all of which produce similar patterns (*SI Appendix*, section K).

This study offers several insights. The findings provide robust evidence for the AI assessment effect based on between- and within-subjects comparisons. Moreover, AI assessment resulted in greater deviations from “the ground truth” (i.e., when no assessment was present) than human assessment. Finally, the study highlights that AI assessment can lead to different people being selected for a position as compared with assessment by humans, suggesting direct consequences for organizations using AI assessment.

### Study 4.

Study 4 (https://aspredicted.org/7WV_L9K) tested the AI assessment effect for behavior in a realistic application context. To this end, we employed a paradigm that asks labor pool workers to make a deliberate choice between presenting themselves as more analytical or more intuitive by ranking a series of attributes, some of which are associated with analytical capabilities. In contrast to the measures in Studies 1 to 3, this one was behavioral and required participants to actively decide which characteristics to prioritize in their self-presentation.

To make the self-presentation consequential and incentive aligned, we (truthfully) informed participants that their performance in this ranking task determined their inclusion in a research pool. The paradigm mimics both prior work (54) and actual procedures common in human resource management practice (55). Depending on condition, participants were informed that their performance on the task would be assessed by an “algorithm” or a “person.” We predicted that people would rank attributes associated with analytical capabilities higher and depict them as more reflective of their capabilities under AI (vs. human) assessment.

The study comprised two stages separated by at least 3 d. Two thousand participants were recruited for stage 1 (*M*_age_ = 39.7 y; 56% female, 42% male). We informed them about the potential to qualify for an exclusive research pool and measured their demographics and beliefs about the required analytical and intuitive capabilities for 15 professional positions (e.g., data analyst, social worker), one of which they would later apply to (i.e., research pool participant).

As preregistered, we launched stage 2 after 3 d, and 1,789 participants (*M*_age_ = 40.7 y; 57% female, 41% male) returned. They were randomly assigned to the AI or human assessment condition. All participants were informed that acceptance into the pool was determined by their performance on a ranking task assessed by either a “qualified algorithm” or a “qualified person” (for assessment protocols, see *SI Appendix*, section L). Then, participants chose how to present themselves by ranking eight attributes—four analytical and four intuitive—in an order that most accurately described them, with the most important one being ranked first. We pretested these attributes for discrimination along the analytical–intuitive continuum and a comparable level of favorability (*SI Appendix*, section L).

#### Self-presentation ranking task.

As preregistered, we compared the rank sum of the four analytical attributes between conditions, where a lower rank sum indicates a more analytical self-presentation. A Wilcoxon rank-sum test demonstrated that participants ranked analytical attributes higher (they showed lower scores) under AI than human assessment (*Mdn*_AI_ = 16 vs. *Mdn*_human_ = 18, *z* = −4.46, *P* < 0.001, *r* = 0.11, 95% CI = [0.06, 0.16]). This finding was robust to alternative analysis strategies (e.g., standardized mean rank differences). The AI assessment effect did not interact with participants’ beliefs about the required analytical and intuitive capabilities for the position they were assessed for (*SI Appendix*, section L).

Study 4 offered evidence for the AI assessment effect employing a real application context and observing actual behavior. We also found mediation via the proposed analytical priority lay belief; however, internal validity of our measure was low, and thus we refrain from discussing the mediation analysis in depth (see *SI Appendix*, section L for the results).

### Studies 5a and 5b.

We argue that the AI assessment effect is explained by an analytical priority lay belief. Whereas Study 1 provided process evidence for this lay belief based on mediation analyses, Studies 5a and 5b tested this lay belief more directly. Study 5a employed a “consider-the-opposite paradigm” (31) asking participants to think about their expectation regarding what the AI focuses on in its assessment and then consider the opposite of this initial belief about AI assessment. Extending this, in Study 5b, we explicitly pointed to the analytical priority lay belief and asked participants to set aside this lay belief and instead consider why AI might be able to prioritize emotions and intuitions as well.

### Study 5a.

#### Pretest.

Study 5a’s pretest established the effectiveness of our consider-the-opposite manipulation (see below). A three-cell between-subjects design (*N* = 441; *M*_age_ = 38.6 y; 56% female, 43% male) with an AI, human (as in Study 4), and an AI-opposite condition demonstrated that the consider-the-opposite paradigm was effective, reducing analytical priority lay beliefs in the AI-opposite condition (*M*_AI_opp_ = 5.19 vs. *M*_human_ = 4.90; *t*(438) = 1.92, *P* = 0.055, *d* = 0.23, 95% CI = [0.01, 0.47]; *M*_AI_ = 5.55 vs. *M*_human_ = 4.90; *t*(438) = 4.45, *P* < 0.001, *d* = 0.50, 95% CI = [0.25, 0.72]; *M*_AI_opp_ = 5.19 vs. *M*_AI_ = 5.55; *t*(438) = −2.32, *P* = 0.021, *d* = −0.27, 95% CI = [−0.51, −0.04]).

#### Main study.

The main study (https://aspredicted.org/th3x-7rss.pdf) was also a three-cell between-subjects experiment. A total of 2,321 participants (*M*_age_ = 40.0 y; 57% female, 42% male) were randomly assigned to the AI, human, or AI-opposite condition. The instructions and the self-presentation ranking task (the key dependent variable) were identical to those used in Study 4, except that we adapted the incentive compatibility, informing participants that the 10 top performers would enter our research pool. In the AI-opposite condition, and before performing the task, participants read the consider-the-opposite manipulation:

*Think for a moment about what you expect the algorithm to be capable of and thus what the algorithm focuses on in its assessment. Before you proceed, we would like you to consider the opposite. Can your expectations about what the algorithm assessor is good at when assessing candidates be wrong? Imagine that you were trying to be as unbiased as possible in evaluating this assessor, consider yourself to be in the same role as a judge or juror. Could the algorithm assessor be good at the opposite of what you expect them to be good at? Please write down some reasons for why and how you could be wrong in terms of your expectations about what the algorithm assessor is good at when assessing people.*

#### Self-presentation ranking task.

A Kruskal–Wallis test on the rank sum of the analytical attributes yielded a group difference (*H*(2, n = 2,321) = 30.85, *P* < 0.001, *η^2^* = 0.01, 95% CI = [0.001, 0.02]), which we qualified with planned contrasts. Participants presented themselves as more analytical under AI (vs. human) assessment (*Mdn*_AI_ = 18 vs. *Mdn*_human_ = 20; *z* = −4.23, *P* < 0.001, *r* = 0.10, 95% CI = [0.05, 0.15]). Importantly, in the AI-opposite condition, participants presented themselves as less analytical than in the AI condition (*Mdn*_AI_ = 18 vs. *Mdn*_AI_opp_ = 20; *z* = −5.15, *P* < 0.001, *r* = 0.13, 95% CI = [0.08, 0.18]) and on levels comparable to the human condition (*z* = −1.25, *P* = 0.210, *r* = 0.03, 95% CI = [0.00, 0.08]). A series of robustness tests provide further evidence for the effect (*SI Appendix*, section M).

### Study 5b.

#### Pretest.

We ran a three-cell between-subjects experiment (*N* = 444; *M*_age_ = 42.3 y; 54% female, 44% male), with an AI, human, and AI-intuitive condition, in which we implemented a stronger manipulation asking participants to consider the emotional and intuitive capacities of the AI (see below). This manipulation further reduced analytical priority lay beliefs, bringing them down to levels comparable to the human condition (*M*_AI_intuitive_ = 5.05 vs. *M*_human_ = 5.01; *t*(441) = 0.27, *P* = 0.79, *d* = 0.03, 95% CI = [−0.23, 0.29]; *M*_AI_ = 5.67 vs. *M*_human_ = 5.01; *t*(441) = 4.51, *P* < 0.001, *d* = 0.51, 95% CI = [0.30, 0.75]; *M*_AI_intuitive_ = 5.05 vs. *M*_AI_ = 5.67; *t*(441) = −4.08, *P* < 0.001, *d* = −0.48, 95% CI = [−0.70, −0.22]).

#### Main study.

In the main study (https://aspredicted.org/Y4K_SB6), participants (*N* = 2,370; *M*_age_ = 37.6 y; 66% female, 31% male) completed Study 4’s ranking task. In the AI-intuitive condition, participants read the following manipulation before performing the task:

*Some people might think that an algorithm is not capable to process emotions and intuition and thus focuses on analytical attributes in assessment… but this is a misjudgment. For a moment, set aside your expectations about the algorithm. Please write down a reason why the algorithm might be able to focus on emotions and intuitions as well.*

#### Self-presentation ranking task.

Again, we found a group difference (*H*(2, n = 2,370) = 21.09, *P* < 0.001, *η^2^* = 0.01, 95% CI = [0.002, 0.02]), which we qualified with planned contrasts. Participants presented themselves as more analytical under AI (vs. human) assessment (*Mdn*_AI_ = 19 vs. *Mdn*_human_ = 20; *z* = −2.61, *P* = 0.009, *r* = 0.06, 95% CI = [0.02, 0.11]). Importantly, participants in the AI-intuitive condition presented themselves as less analytical than those in the AI condition (*Mdn*_AI_ = 19 vs. *Mdn*_AI_intuitive_ = 21; *z* = −4.45, *P* < 0.001, *r* = 0.11, 95% CI = [0.07, 0.17]). In fact, they ranked analytical attributes even lower than those in the human condition (*Mdn*_AI_intuitive_ = 21, *Mdn*_human_ = 20; *z* = −2.30, *P* = 0.021, *r* = 0.06, 95% CI = [0.01, 0.11]), speaking to the explicit nature of the manipulation. Additional analyses provide further evidence for the effect (*SI Appendix*, section N).

Studies 5a and 5b document that the AI assessment effect can be attenuated if participants consider the opposite of their initial beliefs and even reversed when explicitly instructed to contemplate the intuitive capacities of the AI. These findings provide experimental evidence that the analytical priority lay belief drives the AI assessment effect.

### Study 6.

In all previous studies, we disclosed AI vs. human assessment to participants. In hiring practice, however, some organizations disclose the use of AI assessment while emphasizing that the final decision is made by humans (see *SI Appendix*, section O for examples). Study 6 (https://aspredicted.org/3b3v-g3pp.pdf) was inspired by such real-life disclosure instances. In addition to AI and human assessment, in this study, we added a third condition in which participants learned that AI assesses candidates’ performance in the first stages, but that the final decision is made by a human. This three-cell between-subjects design (*N* = 2,370; *M*_age_ = 42.8 y; 57% female, 42% male) allowed us to compare a joint assessment (AI and human) with our focal conditions. As the dependent variable, participants completed the same attribute ranking task as in Study 4.

#### Self-presentation ranking task.

We found a group difference between the three conditions (*H*(2, n = 2,330) = 35.79, *P* < 0.001, *η^2^* = 0.01, 95% CI = [0.01, 0.03]). Planned contrasts revealed that participants again presented themselves as more analytical under AI (vs. human) assessment (*Mdn*_AI_ = 18 vs. *Mdn*_human_ = 20; *z* = −5.97, *P* < 0.001, *r* = 0.15, 95% CI = [0.10, 0.20]). Under joint assessment, participants described themselves as more analytical than under exclusively human assessment (*Mdn*_joint_ = 19 vs. *Mdn*_human_ = 20; *z* = −2.54, *P* = 0.011, *r* = 0.06, 95% CI = [0.01, 0.11]), but as less analytical than under exclusively AI assessment (*Mdn*_AI_ = 18 vs. *Mdn*_joint_ = 19; *z* = −3.38, *P* < 0.001, *r* = 0.09, 95% CI = [0.04, 0.14]). In other words, adding humans to an AI hiring process reduces the AI assessment effect, but does not switch it off completely.

## Discussion

The rise of AI is changing the way people are being assessed across domains, from the workplace to public service. Organizations increasingly rely on AI assessment to increase efficiency, yet we know little about its effects on the behavior of the people under assessment. In this work, we found evidence for the AI assessment effect across 12 studies (*N* = 13,342) and for different dependent variables, contexts, and experimental paradigms. Specifically, people tend to emphasize their analytical characteristics and downplay their emotions and intuition under AI assessment. Our evidence suggests that this effect is driven by the lay belief that AI prioritizes analytical characteristics. Consequently, challenging this lay belief can attenuate the AI assessment effect.

This research contributes to the evolving literature on the psychology of AI (56). Whereas prior work has examined the barriers and enablers of adopting AI, as well as the benefits of its technological advances, we test the psychological and behavioral consequences of AI adoption, specifically in the context of assessment. Although previous research on AI assessment has focused predominantly on outcomes for organizations and candidates (*SI Appendix*, section B), it has largely neglected the possibility that people may behave differently under AI assessment. One notable exception is recent work by Suen and Hung (57, 58). The authors document that in interview settings, different AI interfaces (with varying social presence and transparency) and combinations of human interviewers with and without AI assistance can either increase or decrease faking.

We believe this research has implications for the validity and reliability of AI assessment. If people strategically adjust their behavior in line with their lay beliefs about what AI prioritizes, their true capabilities and/or personalities may not be revealed (22), which may result in distorted decision-making in consequential situations such as college admissions, hiring, and public service provision, as foreshadowed in Study 3. Therefore, we recommend that organizations identify and address the AI assessment effect in their own assessment practices to ensure that they receive authentic candidate responses.

This research has implications also for the growing calls for transparency regarding AI assessment. An important question in this regard pertains to the interaction of transparency regulations with the effect proposed in this research. For example, Article 13 of the European Union AI Act specifies that for high-risk AI applications, such as AI assessment, people need to be informed not only about the use of AI but also about the AI’s capabilities and limitations, including information on how the AI makes decisions (10). Receiving such information could affect the strategic behavioral adjustments observed in this research. If transparency adds a novel bias to assessment, it is important to consider remedies, from correcting lay beliefs to adapting legislation.

Whereas we mainly focused on one of the most prevalent contexts of AI assessment—human resource management—future research may explore the implications of the AI assessment effect across diverse contexts and populations, such as decisions about who is granted access to public services. In this research, we focused exclusively on the analytical–intuitive dimension as our key dependent variable; it may be worth exploring whether AI assessment also prompts changes to other dimensions relevant to candidates’ self-presentation. For example, Study 3’s exploratory analyses revealed that people under AI (vs. human) assessment adjust downward on dimensions of creativity, ethical and social considerations, risk-taking, and effort investment. Additionally, examining the long-term consequences of the effect for people’s life trajectories and psychological well-being could provide valuable insights. With the rise of advanced generative AI, it is also necessary to investigate whether different types of AI (e.g., those that incorporate more intuitive or creative elements) elicit varied responses.

## Materials and Methods

Participation in the presented studies was voluntary and involved minimal risk; participants in all but the field studies gave informed consent, could withdraw their participation at any time, and were above the age of 18. In the exploratory and pilot study, the field nature inhibited us from receiving explicit consent. We complied with all ethical rules of the University of St.Gallen and obtained approval for the pilot and exemption from approval for Studies 1 to 6 from the university’s Institutional Review Board. All analyses were conducted in R 4.2.1, except for the power calculations, which were executed in G*Power 3.1.9.7. Unless noted otherwise, we employed an alpha level of 5% for power analyses. We did not formally test for underlying normal distributions in our analyses. However, in all studies, we obtained CI with bootstrapping and at least 1,000 resamples, which does not rely on assumptions of normal distributions. We report two-sided test statistics unless noted otherwise. Details on the specific analyses can be found in the analysis code on OSF. All studies were conducted on Tivian’s Unipark survey software, except for the exploratory study (company system), the pilot study (Upwork), and Study 2 (Qualtrics). We recruited participants from Prolific for all studies but the exploratory study (actual applicants) and the pilot study (Upwork freelancers). Assignment to conditions in the between-subjects experiments was executed quasi-randomly by the survey software, following a uniform distribution. All reported results held when including excluded participants.

### Pilot Study.

As preregistered, we invited participants for a total duration of 21 d on the Upwork platform. To maintain a quasi-random assignment and prevent overlap, we alternated our invitations between the two open positions. After sending a total of 70 invites for the first position (the system’s maximum number of invites), we switched to the second position, ensuring that freelancers already invited to one were excluded from the other. By documenting our invitations meticulously (see OSF) and alternating the order of invitations in subsequent batches, we aimed to minimize potential biases. Although the positions were posted from independent accounts, we cannot rule out the possibility that some freelancers might have encountered both postings; where applicable we excluded those freelancers from our data. In scope of the platform’s limitations, we invited a total of 1,861 freelancers, of whom 354 applied. As preregistered, we excluded 60 candidates who did not answer our questions at all, answered inappropriately (e.g., no number or clearly interpretable response), or indicated that they did not believe the task to be genuine, which left us with 294 valid replies. We had to remove 16 freelancers who applied to both positions, for a final sample of 278 (*N*_AI_ = 148, *N*_human_ = 130).

### Study 1.

For Study 1, we calculated a required sample size of 506 participants to detect a small to medium effect size of *d* = 0.25 at 80% power in a two-cell between-subjects design. We advertised the study to 525 participants to account for exclusions and received 529 complete responses. There was no difference in attrition between experimental conditions (*N*_AI_ = 3, *N*_human_ = 2, *P* = 1). We used the following attention checks: “Have you ever been involved in an accident that resulted in your own death?” (“Yes,” “No”) and “What do you get when you add the numbers 5 and seven together? (“2,” “12,” “14”). In line with the preregistration, 16 participants were excluded after failing at least one of two included attention checks, resulting in a final sample size of 513 (*N*_AI_ = 258, *N*_human_ = 255). We further measured how much experience participants had with college applications (1 = “no experience at all” to 7 = “a lot of experience”). The conceptual replications of this study are reported in *SI Appendix*, sections F to I.

### Study 2.

For Study 2, we required 1,160 participants to detect small effects (*f*^2^ = 0.01) in the linear models at a power of 80% and an alpha of 1% (to account for interaction effects). Due to the two-stage nature of this study, we advertised the study to 1,500 participants and received 1,499 complete responses. We chose the representative sampling option on Prolific selecting participants in accordance with U.S. Census data for age, gender, and ethnicity. We report sample descriptives for these three variables in *SI Appendix*, section J. Thirteen participants were excluded failing the same attention checks as in Study 1 for a sample of 1,486. In stage 2, we obtained 1,177 full responses, with no difference in attrition between experimental conditions at stage 2 (*N*_AI_ = 7, *N*_human_ = 2; *P* = 0.184). We report differences in participants returning versus not in *SI Appendix*, section J. Seven participants failed the same attention checks, two did not provide consent yet were not filtered out automatically, and one participant took the study twice, leaving a final sample of 1,166 participants (*N*_AI_ = 581, *N*_human_ = 585). Further information on this study is presented in *SI Appendix*, section J.

### Study 3.

In Study 3, we calculated a required sample size of 1,200 to detect a small group difference (*d* = 0.2) at 80% power for the stage 1 between-subjects comparison. For the within-subjects comparison across stages, this allowed us to detect a small difference (*d* = 0.2) with 98% power. We advertised the study to 1,500 participants to account for attrition at stage 2. We ended up with 1,518 complete responses and there was no difference in attrition between experimental conditions at stage 1 (*N*_AI_ = 17, *N*_human_ = 9, *N*_no_ = 9; *P* = 0.155). In line with the preregistration, 33 participants were excluded at stage 1, resulting in a final sample of 1,485 (*N*_AI_ = 495, *N*_human_ = 492, *N*_no_ = 498). We used the same attention checks for exclusion as in prior studies. In stage 2, we received 1,165 complete responses, with attrition marginally different across experimental conditions at stage 2 (*N*_human_ = 42, *N*_AI_ = 26, *P* = 0.062). We report differences in participants returning versus not in *SI Appendix*, section K. Twenty-seven participants failed at least one of the attention checks, resulting in a final sample of 1,138 participants (*N*_AI_ = 579, *N*_human_ = 559). Further information on this study is presented in *SI Appendix*, section K.

### Study 4.

In Study 4, based on an expected effect size of *d* = 0.15 at 80% power, we calculated a required sample size of 1,464 for a two-cell between-subjects design. Given the study’s two-stage design, we advertised the first stage to 2,000 participants. We received 2,000 complete responses to the first stage, and 1,834 participants returned to complete the second stage, with no differences in attrition between experimental conditions (*N*_AI_ = 14, *N*_human_ = 14, *P* = 1). We report differences in participants returning versus not in *SI Appendix*, section L. Of these, 43 failed at least one out of two attention checks (see Study 1), and one participant filled out the survey twice, resulting in a final sample size of 1,789 (*N*_AI_ = 895, *N*_human_ = 894). In the second stage, we measured AI involvement in the assessment (“The assessment evaluation was a process involving me and …” 1 = “another person only,” 4 = “both another person and an algorithm,” 7 = “an algorithm only”). Further, we gauged whether participants believed in the consequentiality of the study (“Do you believe that this study was designed to recruit for a research pool?”; “Yes,” “No”), and included a hypothesis-guessing question in the form of an open text field (“In your own view, what do you think is the research team interested in?”). Eventually, we assessed participants’ self-monitoring tendencies (59) (α = 0.88). Further information on this study is presented in *SI Appendix*, section L.

### Studies 5a and 5b.

In both studies, we aimed for 2,400 participants, a comparable sample size per cell as in Study 4, which would give us 99.5% power to detect a small group difference (*η^2^* = 0.01).

In Study 5a, we received 2,376 complete responses. Attrition differed between conditions (*N*_AI_ = 27, *N*_human_ = 28, *N*_AI_opp_ = 196, *P* < 0.001). We explain this by participants dropping out on the writing task page (*N* = 179). Fifty-five participants failed at least one attention check (see Study 1), resulting in a final sample size of 2,321 (*N*_AI_ = 828, *N*_human_ = 826, *N*_AI_opp_ = 667). As in Study 4, we gauged whether participants believed in the consequentiality of the study and included a hypothesis-guessing question. Further information on Study 5a can be found in *SI Appendix*, section M.

In Study 5b, we received 2,446 complete responses. Attrition differed between conditions (*N*_AI_ = 31, *N*_human_ = 34, *N*_AI_intuitive_ = 144, *P* < 0.001), which we again explain by participants dropping out on the writing task page (n = 134). Seventy-six participants failed at least one attention check (see Study 1), resulting in a final sample size of 2,370 (*N*_AI_ = 825, *N*_human_ = 829, *N*_AI_intuitive_ = 716). We again gauged whether participants believed in the consequentiality of the study, included a hypothesis-guessing question, and assessed self-monitoring tendencies. Further information on Study 5b can be found in *SI Appendix*, section N.

### Study 6.

As in Studies 5a and 5b, in Study 6, we aimed for 2,400 participants. We received 2,387 complete responses. Attrition did not differ between conditions (*N*_AI_ = 39, *N*_human_ = 35, *N*_joint_ = 46, *P* = 0.440). Fifty-seven participants failed at least one attention check (see Study 1), resulting in a final sample size of 2,330 (*N*_AI_ = 774, *N*_human_ = 788, *N*_joint_ = 768). Further information on Study 6 can be found in *SI Appendix*, section O.

## Supplementary Material

Appendix 01 (PDF)

## Data Availability

Data collected from Prolific and Upwork, preregistrations, and the analysis code are available on OSF (38). For more information on the exploratory study, readers can reach out to the corresponding author.
