# Supplementary material for: AI assessment changes human behavior
Source: Proc Natl Acad Sci U S A. 2025 Jun 20;122(25):e2425439122. doi: 10.1073/pnas.2425439122 (PMC12207480; doi:10.1073/pnas.2425439122)
Supplement: Supplementary file 1 — Appendix 01 (PDF) [file pnas.2425439122.sapp.pdf]

Supporting Information for
AI assessment changes human behavior

Jonas Goergen, Institute of Behavioral Science and Technology, University of St.Gallen, 9000 St.Gallen, Switzerland

Emanuel de Bellis, Institute of Behavioral Science and Technology, University of St.Gallen, 9000 St.Gallen, Switzerland

Anne-Kathrin Klesse, Rotterdam School of Management, Erasmus University Rotterdam, 3062 PA Rotterdam, The Netherlands

*Emanuel de Bellis, Torstrasse 25, 9000 St.Gallen, +41 71 224 7712

Email: emanuel.debellis@unisg.ch

This PDF file includes:

Figures S1 to S18
Tables S1 to S105

Supplementary Information A: Exploratory Study

a. Detailed reporting

To explore the potential consequences of AI assessment for candidate behavior, we collaborated with an employment software company (https://aspredicted.org/KGB_LXV). The company positions itself to recruiters as offering game-based assessment, instead of the traditional CV-based assessment, to reduce biases in hiring. They do not use AI in their assessment.

Applicants for 74 positions offered by 59 organizations in various industries went through the company's game-based assessment and could subsequently participate in a voluntary follow-up survey. We included two questions at the end of this survey, which ran between 07 Feb 2024 and 15 Apr 2024. Of the total 9,196 applicants in our time window, 1,421 (M_{age} = 28.7 years; 37% female, 62% male) completed the survey. On a seven-point scale, we first measured applicants' perception of whether the assessment is "exclusively made by a human" (1), "exclusively made by an algorithm" (7), or a combination of both (2–6). They were then asked to indicate the extent to which they adapted their behavior during the assessment to the assessor (i.e., "To what extent did you adjust your in-game behavior for this anticipated reviewer?"; 1 = "no adjustment at all" to 7 = "strong adjustment"). Moreover, in consultation with the company, we decided to explore whether applicants' perception of the assessor correlates with a problem-solving score, capturing the way applicants approached the games in the assessment. We received problem-solving scores in the form of standardized values for every participant. According to the company, higher scores were indicative of more systematic thinking. However, for confidentiality reasons, we were not provided more information about this score, except for the fact that temporal metrics were factored in.

Regarding applicants' perception of who is assessing them, we found that, on average, applicants suspected the involvement of an algorithm in the assessment, indicated by a median of 5 and a mean of 4.69 (one-sided test from midpoint: $t(1{,}420) = 17.85$, $P < .001$, $d = 0.47$, 95% CI = [0.42, 0.53]). Out of all applicants, 12.74% assumed algorithm-based assessment exclusively, 84.52% a combination of algorithm- and human assessment, and 2.74% human assessment exclusively. Second, the more applicants suspected algorithm involvement in their assessment, the stronger the self-reported adjustment of their behavior ($r(1{,}407) = .12$, $P < .001$, 95% CI = [.06, .18]).

We further pre-registered a correlation between the problem-solving score and the perception of algorithmic assessment. Contrary to the pre-registration, we did not find such a correlation ($r(1{,}420) = -.03$, $P = .51$, 95% CI = [−0.03, 0.07]). In retrospect, this is not surprising because candidates had no information on the game and its purpose, and thus likely lacked insights into what behavior within the game would showcase analytical (vs. intuitive) capabilities—a necessary condition for the AI assessment effect to occur. In addition, we cannot examine the composition of the problem-solving score, making it impossible for us to certify a match between the score and our conceptualization of self-presentational behavior.

This exploratory study offered two preliminary insights. First, a large share of applicants assumed that they were assessed by an algorithm, despite the company not providing such information. Second, the greater the extent to which the applicants assumed algorithm involvement, the stronger the adjustment of their reported behavior.

b. Full list of variables

- Company
- Job title
- Ethnicity
- Age
- Gender
- Country of residence
- Highest education degree completed

- ASD (autism spectrum disorder) diagnosed
- ADHD (attention deficit hyperactivity disorder) diagnosed
- OCD (obsessive-compulsive disorder) diagnosed
- CB (color blindness) diagnosed
- Dyslexia diagnosed
- Subscribed to newsletter
- Epilepsy diagnosed
- Ethnic or racial minority
- LGBTQ
- Gender minority
- Religious minority
- Other minority
- Perception human vs. algorithm
- Adjusted behavior
- Problem-solving score

c. **Descriptive statistics**

Table S1: Descriptive statistics.

Variable	Frequency (%) / means (SD)
Gender	
Female	525 (36.95%)
Male	881 (62.00%)
Non-binary	4 (0.28%)
Not disclosed	11 (0.77%)
Age (years)	28.67 (8.52)
Perception human vs. algorithm	4.69 (1.45)
Adjusted behavior	3.26 (1.91)
No response	12
Problem-solving score	−0.21 (1.03)

N = 1,421

d. **Perception of human versus algorithm**

Table S2: Frequency of scale points for perception of algorithm involvement in assessment.

Variable	Frequency (%)
Perception human vs. algorithm (categories)	
Exclusively made by an algorithm (7)	181 (12.74%)
Exclusively made by a human (1)	39 (2.74%)
Combination of both (2–6)	1,201 (84.52%)

N = 1,421

Supplementary Information B: Literature Table

Table S3: Overview of exemplary prior research on AI assessment across different disciplines, with a focus on the behavioral sciences.

Research focus	Main topics	Selected references
Objective assessment outcomes	Results, performance, and bias	Abduraham et al., 2024 (1); Campion et al., 2024 (2); Chalfin et al., 2016 (3); Curto et al., 2024 (4); Fan et al. 2023 (5); Hickman et al., 2021 (6); Lambrecht and Tucker, 2019 (7); Liff et al., 2024 (8); Lira et al., 2023 (9); Zhang et al., 2021 (10)
Subjective perceptions and attitudes	Trust and acceptance	Deriu et al., 2024 (11); Fumagalli et al., 2022 (12); Gonzalez et al., 2022 (13); Kares et al., 2023 (14); Langer et al., 2019 (15); Mirowska and Mesnet, 2022 (16); Raveendhran and Fast, 2021 (17); Schick and Fischer, 2021 (18)
	Fairness and ethics	Acikgoz et al., 2020 (19); Bedemariam and Wessel, 2023 (20); Bonezzi and Ostinelli, 2021 (21); Bigman et al., 2022 (22); Feldkamp et al., 2024 (23); Hilliard et al. 2022 (24); Jago and Laurin, 2022 (25); Ochmann et al., 2024 (26); Yan et al., 2024 (27)
Behavioral consequences	Behavior during assessment	*The current research*

Supplementary Information C: Empirical Overview

Table S4: Overview of studies included in the manuscript.

Study	Key Objective	Sample and Design	Measures	Key Finding
Pilot	Show the AI assessment effect in the field	*N* = 278 Upwork freelancers<br>Two-cell (AI vs. human assessment) quasi-experiment	Dependent variable (DV): Pre-screening question on analytical (vs. intuitive) self-description	AI assessment effect: More analytical self-description under AI (vs. human) assessment
1	Show the AI assessment effect under controlled conditions	*N* = 513 research pool participants<br>Two-cell (AI vs. human assessment) between-subjects experiment	DV: Analytical and intuitive task approach<br>Mediator: Analytical priority lay belief	AI assessment effect: More analytical and less intuitive task approach under AI (vs. human) assessment
2	Test robustness of the AI assessment effect by exploring potential moderators	*N* = 1,166 research pool participants<br>Two-stage between-subjects design (AI vs. human assessment)	DV: Analytical vs. intuitive task approach<br>Individual differences: AI receptiveness, job application context, personality	Replicate the AI assessment effect<br>The AI assessment effect is moderated by age, fear of negative evaluation, agreeableness, and occupation
3	Test robustness of the AI assessment effect employing within- and between-subjects design	*N* = 1,138 research pool participants<br>Two-stage mixed design (stage 1: AI vs. human vs. no assessment; stage 2: AI vs. human assessment)	DV: Analytical task approach<br>Exploratory measures: creativity, effort invested, ethical and social considerations, and risk-taking	Replicate the AI assessment effect (1) compared to no assessment and (2) for within-subjects design
4	Show the AI assessment effect using a behavioral outcome variable	*N* = 1,789 research pool participants<br>Two-stage between-subjects design (AI vs. human assessment)	DV: Self-description ranking<br>Individual difference: Required capabilities for position	Replicate the AI assessment effect for behavioral dependent variable
5a	Manipulate the analytical priority lay belief	*N* = 2,321 research pool participants<br>Three-cell between-subjects design (AI vs. human vs. consider-the-opposite AI)	DV: Self-description ranking<br>Manipulation check: Analytical priority lay belief	Replicate the AI assessment effect<br>AI assessment effect vanishes when considering the opposite of initial beliefs about AI

5b	Manipulate the analytical priority lay belief	*N* = 2,370 research pool participants<br><br>Three-cell between-subjects design (AI vs. human assessment vs. consider the AI's intuitive capabilities)	DV: Self-description ranking<br><br>Manipulation check: Analytical priority lay belief	Replicate the AI assessment effect<br><br>AI assessment effect reverses when considering AI's intuitive capabilities
6	Compare different disclosure practices (AI vs. human vs. AI and human jointly)	*N* = 2,330 research pool participants<br><br>Three-cell between-subjects design (AI vs. human vs. AI and human joint assessment)	DV: Self-description ranking	Replicate AI assessment effect<br><br>AI assessment effect persists for AI and human joint assessment

Note: Four conceptual replications of Study 1 reported in SI-F to SI-I.

Supplementary Information D: Pilot Study

a. **Stimuli**

Figure S1: Stimuli used in AI and human conditions.

AI condition

📢 🧑 📢 PLEASE NOTE: YOUR APPLICATION WILL BE EVALUATED BY OUR RECRUITER 📢 🧑 📢

We are looking for an experienced event planning freelancer to support us in organizing the Consumer-Technology Dialogue 2026, a high-profile academic event in Switzerland. This event will bring together researchers from across Europe to discuss cutting-edge insights into consumer interactions with technology.

✅ Venue Research & Coordination – Identifying and evaluating venue options in Switzerland.
✅ Program Development – Supporting the structuring of sessions, speaker coordination, and scheduling.
✅ Content Strategy – Assisting in crafting key messaging, communication materials, and engagement strategies.
✅ General Event Support – Handling additional organizational tasks as needed.

What We're Looking For

🔹 Event Planning Experience – Prior experience in academic, corporate, or professional event planning.
🔹 Organizational Skills – Ability to manage multiple tasks, timelines, and stakeholders effectively.
🔹 Communication & Coordination – Strong written and verbal communication skills.
🔹 Problem-Solving & Adaptability – Comfortable navigating challenges and adjusting plans when needed.

📢 🧑 📢 PLEASE NOTE: YOUR APPLICATION WILL BE EVALUATED BY OUR RECRUITER 📢 🧑 📢

Human condition

📢 🤖 📢 PLEASE NOTE: YOUR PROPOSAL IS EVALUATED BY OUR ARTIFICIAL INTELLIGENCE (AI) TOOL 📢 🤖 📢

We are looking for an experienced event planning freelancer to support us in organizing the Consumer-Technology Dialogue 2026, a high-profile academic event in Switzerland. This event will bring together researchers from across Europe to discuss cutting-edge insights into consumer interactions with technology.

✅ Venue Research & Coordination – Identifying and evaluating venue options in Switzerland.
✅ Program Development – Supporting the structuring of sessions, speaker coordination, and scheduling.
✅ Content Strategy – Assisting in crafting key messaging, communication materials, and engagement strategies.
✅ General Event Support – Handling additional organizational tasks as needed.

What We're Looking For

🔹 Event Planning Experience – Prior experience in academic, corporate, or professional event planning.
🔹 Organizational Skills – Ability to manage multiple tasks, timelines, and stakeholders effectively.
🔹 Communication & Coordination – Strong written and verbal communication skills.
🔹 Problem-Solving & Adaptability – Comfortable navigating challenges and adjusting plans when needed.

📢 🤖 📢 PLEASE NOTE: YOUR PROPOSAL IS EVALUATED BY OUR ARTIFICIAL INTELLIGENCE (AI) TOOL 📢 🤖 📢

b. **Measures**

- Key dependent variable: Please describe yourself to convince the recruiter assessing you. Are you more of an analytical or intuitive person? Write down a number between 1 and 7, with 1 = strongly intuitive and 7 = strongly analytical.
- Exploratory dependent variable: Event planning can be approached in very different ways. Use FOUR KEYWORDS to describe your task approach and convince the recruiter.

c. **Key result robustness check**

Table S5: Results for ANCOVA on key dependent variables, including covariate gender.

Dependent variable	Comparison	*df*	*t*	*P*	*d*	95% CI
Analytical task approach	AI–human	273	2.14	.033	0.25	[0.003, 0.50]

d. **Exploratory variable analysis**

We pre-registered to run an exploratory analysis on a second dependent variable, which asked applicants to provide four keywords describing their approach to the event planning task. Our hypothesis was that participants under AI assessment would use keywords more reflective of analytical approaches compared to human assessment. Accordingly, we submitted all reported keywords to a Prolific sample (N = 184; M_{age} = 38.2 years; 60% female); each participant rated 50 keywords on the extent to which they can be considered reflective of an intuitive or analytical approach to the event planning task. We then compared the average scores across keywords for every applicant between the AI and human conditions, as pre-registered. We did not find the expected difference between the AI (M = 4.27) and human (M = 4.31) condition (t(271) = −0.83, *P* = .409, d = −0.10, 95% CI [−0.35, 0.13]).

Table S6: Results for independent samples t-test on the task approach reflected in keyword use.

Dependent variable	Comparison	*df*	*t*	*P*	*d*	95% CI
Analytical task approach	AI–human	271	−0.83	.409	−0.10	[−0.35, 0.13]

We speculate that this lack of a difference is due to at least two reasons: First, a potential lack of power to detect an effect. Second, we noticed that the majority of participants used keywords included in our vacancy description, which suggests that participants may have copy-pasted characteristics that seemed appreciated rather than offering their own keywords (see below). The fact that many participants kept their keywords decisively close to the task description may have made it difficult to detect an influence of our manipulation on the reported task approach.

Table S7: Word count for top 20 keywords total and by condition.

Term	Total	AI	Human
detail-oriented	109	60	49
strategic	95	56	39
adaptive	77	49	28
organized	74	45	29
collaborative	71	34	37
adaptable	43	21	22
proactive	42	17	25
creative	24	14	10
communication	17	11	6
structured	16	12	4
organization	13	9	4
adaptability	12	7	5
flexible	12	8	4
meticulous	12	7	5
planning	12	7	5
resourceful	12	5	7
intuitive	11	3	8
coordination	10	7	3
efficient	9	6	3
research	9	5	4

Note: To evaluate the analytical or intuitive valence of each word we kept the original words. Specifically, we did not stem words, which would harden interpretation for the participants rating the words and we did not recode words unless for obvious writing errors. We deemed the choice

of, for example, nouns over adjectives as conveying information of interest (e.g., adaptable and adaptability). A full list of unique words is available on OSF.

e. **Assessment**

As pre-registered, to select one candidate, we provided both ChatGPT 4o and an RA the same text and dataset below.

Figure S2: ChatGPT 4o prompting and output screenshots.

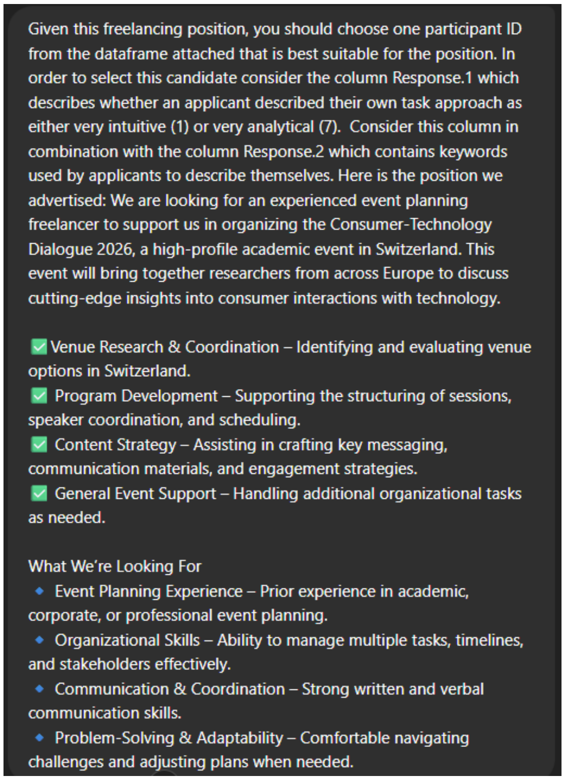

Given this freelancing position, you should choose one participant ID from the dataframe attached that is best suitable for the position. In order to select this candidate consider the column Response.1 which describes whether an applicant described their own task approach as either very intuitive (1) or very analytical (7). Consider this column in combination with the column Response.2 which contains keywords used by applicants to describe themselves. Here is the position we advertised: We are looking for an experienced event planning freelancer to support us in organizing the Consumer-Technology Dialogue 2026, a high-profile academic event in Switzerland. This event will bring together researchers from across Europe to discuss cutting-edge insights into consumer interactions with technology.

✅ Venue Research & Coordination – Identifying and evaluating venue options in Switzerland.
✅ Program Development – Supporting the structuring of sessions, speaker coordination, and scheduling.
✅ Content Strategy – Assisting in crafting key messaging, communication materials, and engagement strategies.
✅ General Event Support – Handling additional organizational tasks as needed.

What We're Looking For
🔹 Event Planning Experience – Prior experience in academic, corporate, or professional event planning.
🔹 Organizational Skills – Ability to manage multiple tasks, timelines, and stakeholders effectively.
🔹 Communication & Coordination – Strong written and verbal communication skills.
🔹 Problem-Solving & Adaptability – Comfortable navigating challenges and adjusting plans when needed.

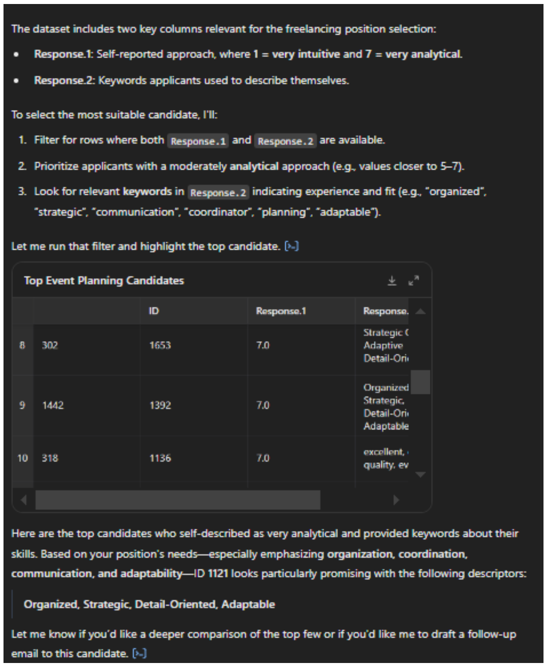

The dataset includes two key columns relevant for the freelancing position selection:

- **Response.1**: Self-reported approach, where 1 = **very intuitive** and 7 = **very analytical**.
- **Response.2**: Keywords applicants used to describe themselves.

To select the most suitable candidate, I'll:

1. Filter for rows where both `Response.1` and `Response.2` are available.
2. Prioritize applicants with a moderately **analytical** approach (e.g., values closer to 5–7).
3. Look for relevant **keywords** in `Response.2` indicating experience and fit (e.g., "organized", "strategic", "communication", "coordinator", "planning", "adaptable").

Let me run that filter and highlight the top candidate. [>-]

Top Event Planning Candidates

		ID	Response.1	Response.
8	302	1653	7.0	Strategic (Adaptive Detail-Ori
9	1442	1392	7.0	Organized Strategic, Detail-Ori Adaptable
10	318	1136	7.0	excellent, quality, ev

Here are the top candidates who self-described as very analytical and provided keywords about their skills. Based on your position's needs—especially emphasizing **organization, coordination, communication, and adaptability**—**ID 1121** looks particularly promising with the following descriptors:

> **Organized, Strategic, Detail-Oriented, Adaptable**

Let me know if you'd like a deeper comparison of the top few or if you'd like me to draft a follow-up email to this candidate. [>-]

Supplementary Information E: Study 1

a. **Scenario description**

For the remainder of the survey, consider the following scenario.

You apply for admission at Horizon University.
Right at the beginning of the assessment, Horizon University informs you:
Your application today will be evaluated by a person [an artificial intelligence].
It is solely this person [AI] that will monitor and assess your performance in the process and examine your responses. The person's [AI's] performance review decides whether you proceed to a next round.

With this information at hand, how would you approach this assessment?

b. **Measures**

Analytical task approach

- I would approach these tasks analytically.
- I would tackle these tasks systematically.
- I would apply precise rules to deduce the answers.
- I would figure things out logically.
- I would use clear rules.

All items measured on seven-point Likert scales from 1 = "strongly disagree" to 7 = "strongly agree."

Intuitive task approach

- I would trust my hunches.
- I would go by what felt good to me.
- I would use my gut feelings.
- I would rely on my sense of intuition.
- I would use my heart as a guide for my actions.

All items measured on seven-point Likert scales from 1 = "strongly disagree" to 7 = "strongly agree."

Analytical priority lay belief

We would like to understand better how you perceive the human (AI) evaluator from before. Please indicate your agreement with the following statements.

- Analytical attributes take precedence over intuitive ones for the human (AI).
- The focus of the human (AI) is on analytical attributes rather than intuitive ones.
- The human (AI) places an emphasis on analytical attributes rather than intuitive ones.

All items measured on seven-point Likert scales from 1 = "strongly disagree" to 7 = "strongly agree."

c. **Descriptive statistics**

Table S8: Descriptive statistics for key items in Study 1.

Variable	Frequency (%) / means (SD)
Gender	
Female	306.00 (59.65%)
Male	196.00 (38.21%)
Diverse	7.00 (1.36%)
Not disclosed	4.00 (0.78%)
Age (years)	37.43 (13.43)
Analytical task approach	5.80 (0.94)
Intuitive task approach	4.51 (1.45)

Variable	Frequency (%) / means (SD)
Analytical priority lay belief	5.00 (1.53)
College application experience	4.72 (1.70)
Education	
Less than high school	2.00 (0.39%)
Master	73.00 (14.23%)
Bachelor	190.00 (37.04%)
Other	49.00 (9.55%)
High school	186.00 (36.26%)
Doctorate	13.00 (2.53%)
Occupation	
Unemployed	77.00 (15.01%)
Retired	31.00 (6.04%)
Self-employed	72.00 (14.04%)
Employed	254.00 (49.51%)
Student	68.00 (13.26%)
Other	11.00 (2.14%)

N = 513

Note: A coding error on Prolific prevented us from capturing information on participants' ethnicity and approved Prolific tasks.

d. **Task approach results**

Table S9: Results for independent samples t-tests on key dependent variables.

Dependent variable	Comparison	*df*	*t*	*P*	*d*	95% CI
Analytical task approach	AI–human	511	4.95	< .001	0.44	[0.27, 0.62]
Intuitive task approach	AI–human	511	−4.93	< .001	−0.43	[−0.61, −0.25]

Table S10: Results for ANCOVA on key dependent variables, including covariates age, gender, education, occupation, and college application experience.

Dependent variable	Comparison	*df*	*t*	*P*	*d*	95% CI
Analytical task approach	AI–human	506	5.01	< .001	0.44	[0.26, 0.63]
Intuitive task approach	AI–human	506	−5.05	< .001	−0.43	[−0.60, −0.24]

e. **Analytical priority lay belief results**

Table S11: Results for independent samples t-test on mediator.

Dependent variable	Comparison	*df*	*t*	*P*	*d*	95% CI
Analytical priority lay belief	AI–human	511	13.98	< .001	1.23	[1.02, 1.46]

Table S12: Regression results for mediation model: Analytical task approach.

	Analytical task approach	Analytical priority lay belief	Analytical task approach
(Intercept)	5.796***	4.996***	5.796***
	(0.041)	(0.057)	(0.039)
Condition	0.402***	1.606***	0.107
	(0.081)	(0.115)	(0.092)
Analytical priority lay belief			0.183***
			(0.030)
R^2	0.046	0.277	0.110
Adj. R^2	0.044	0.275	0.107

	Analytical task approach	Analytical priority lay belief	Analytical task approach
Number obs.	513	513	513

Note: Unstandardized regression coefficients are displayed, with standard errors in parentheses.
*P < .05, **P < .01, ***P < .001
Indirect effect: b = 0.30 (0.07), P < .001, 95% CI = [0.17, 0.44]

Table S13: Regression results for mediation model: Intuitive task approach.

	Intuitive task approach	Analytical priority lay belief	Intuitive task approach
(Intercept)	4.508***	4.996***	4.508***
	(0.062)	(0.057)	(0.061)
Condition	–0.615***	1.606***	–0.265
	(0.125)	(0.115)	(0.144)
Analytical priority lay belief			–0.218***
			(0.047)
R^2	0.045	0.277	0.084
Adj. R^2	0.043	0.275	0.080
Number obs.	513	513	513

Note: Unstandardized regression coefficients are displayed, with standard errors in parentheses.
*P < .05, **P < .01, ***P < .001
Indirect effect: b = −0.35 (0.09), P < .001, 95% CI = [−0.52, −0.18]

f. **Key results without exclusions**

Table S14: Results for independent samples t-tests on key dependent variables and mediator.

Dependent variable	Comparison	*df*	*t*	*P*	*d*	95% CI
Analytical task approach	AI–human	527	4.79	< .001	0.42	[0.24, 0.59]
Intuitive task approach	AI–human	527	−5.01	< .001	−0.44	[−0.61, −0.25]
Analytical priority lay belief	AI–human	527	14.07	< .001	1.22	[1.02, 1.43]

Table S15: Regression results for mediation model: Analytical task approach.

	Analytical task approach	Analytical priority lay belief	Analytical task approach
(Intercept)	5.796***	4.999***	5.796***
	(0.040)	(0.056)	(0.039)
Condition	0.384***	1.587***	0.098
	(0.080)	(0.113)	(0.091)
Analytical priority lay belief			0.180***
			(0.030)
R^2	0.042	0.273	0.103
Adj. R^2	0.040	0.272	0.100
Number obs.	529	529	529

Note: Unstandardized regression coefficients are displayed, with standard errors in parentheses.
*P < .05, **P < .01, ***P < .001
Indirect effect: b = 0.29 (0.07), P < .001, 95% CI = [016, 0.43]

Table S16: Regression results for mediation model: Intuitive task approach.

	Intuitive task approach	Analytical priority lay belief	Intuitive task approach
(Intercept)	4.519***	4.999***	4.519***
	(0.061)	(0.056)	(0.060)
Condition	–0.611***	1.587***	–0.279*
	(0.122)	(0.113)	(0.140)
Analytical priority lay belief			–0.209***
			(0.046)
R^2	0.045	0.273	0.081
Adj. R^2	0.044	0.272	0.078
Number obs.	529	529	529

Note: Unstandardized regression coefficients are displayed, with standard errors in parentheses.
*$P < .05$, **$P < .01$, ***$P < .001$
Indirect effect: $b = -0.33$ (0.08), $P < .001$, 95% CI = [−0.50, −0.17]

Supplementary Information F: Conceptual Replication Study 1: Adjusted Scenario

This study replicated Study 1 with slight adjustments in the scenario and added a second dependent variable (indicated below using *italics*). We further measured participants' need for cognition and faith in intuition in this study.

a. Scenario description

For the remainder of the survey, consider the following scenario.

You apply for admission at Horizon University.
Right at the beginning of the assessment, Horizon University informs you:
Your application today will be evaluated by a person [an artificial intelligence].
It is solely this person [AI] that will monitor and assess your performance in the process and examine your responses. The person's [AI's] performance review decides whether you proceed to a next round.

With this information at hand, how would you approach this assessment?
Please indicate whether you would rather focus on the analytical or intuitive side of yours by responding to all items below.

b. Measures

See SI-B

Reliance on cognition versus affect
I would rather rely on...
- …"my thoughts" (1) to "my feelings" (7)
- ... "the rational side of me" to "the emotional side of me" (7)
- … "my head" (1) to "my heart" (7)

c. Descriptive statistics

Table S17: Descriptive statistics.

Variable	Frequency (%) / means (SD)
Gender	
Female	289.00 (57.00%)
Male	205.00 (40.43%)
Diverse	9.00 (1.78%)
Not disclosed	4.00 (0.79%)
Age	39.88 (12.86)
Analytical task approach	5.59 (1.04)
Intuitive task approach	4.10 (1.47)
Reliance cognition vs. affect	2.71 (1.56)
Analytical priority lay belief	5.57 (1.56)
College application experience	4.64 (1.75)
Need for cognition	4.78 (0.85)
Faith in intuition	3.56 (0.80)
Education	
Less than high school	2.00 (0.39%)
Master	74.00 (14.60%)
Bachelor	231.00 (45.56%)
Other	46.00 (9.07%)
High school	138.00 (27.22%)
Doctorate	16.00 (3.16%)
Occupation	
Unemployed	49.00 (9.66%)

Variable	Frequency (%) / means (SD)
Retired	24.00 (4.73%)
Self-employed	62.00 (12.23%)
Employed	337.00 (66.47%)
Student	24.00 (4.73%)
Other	11.00 (2.17%)
Approved Prolific Tasks	1,836.10 (2,104.10)
Ethnicity	
Asian	66.00 (13.02%)
Black	64.00 (12.62%)
No Consent	2.00 (0.39%)
Expired data	14.00 (2.76%)
Mixed	29.00 (5.72%)
Other	21.00 (4.14%)
White	311.00 (61.34%)

N = 507

d. **Task approach results**

Table S18: Results for independent samples t-tests on key dependent variables.

Dependent variable	Comparison	*df*	*t*	*P*	*d*	95% CI
Analytical task approach	AI–human	505	6.02	< .001	0.53	[0.35, 0.71]
Intuitive task approach	AI–human	505	−5.89	< .001	−0.52	[−0.70, −0.35]
Reliance cognition vs. affect	AI–human	505	−7.60	< .001	−0.68	[−0.86, −0.49]

Table S19: Results for ANCOVA on key dependent variables, including covariates age, gender, education, occupation, approved tasks, and ethnicity.

Dependent variable	Comparison	*df*	*t*	*P*	*d*	95% CI
Analytical task approach	AI–human	496	5.94	< .001	0.53	[0.35, 0.70]
Intuitive task approach	AI–human	496	−5.74	< .001	−0.52	[−0.72, −0.33]
Reliance cognition vs. affect	AI–human	496	−7.43	< .001	−0.68	[−0.84, −0.48]

e. **Analytical priority lay belief results**

Table S20: Results for independent samples t-tests on mediator.

Dependent variable	Comparison	*df*	*t*	*P*	*d*	95% CI
Analytical priority lay belief	AI–human	505	13.52	< .001	1.20	[1.00, 1.41]

Table S21: Regression results for mediation model: Analytical task approach.

	Analytical task approach	Analytical priority lay belief	Analytical task approach
(Intercept)	5.593***	5.570***	5.593***
	(0.045)	(0.060)	(0.039)
Condition	0.540***	1.611***	–0.038
	(0.090)	(0.119)	(0.092)
Analytical priority lay belief			0.359***
			(0.029)
R^2	0.067	0.266	0.279
Adj. R^2	0.065	0.264	0.276
Number obs.	507	507	507

Note: Unstandardized regression coefficients are displayed, with standard errors in parentheses.
**P* < .05, ***P* < .01, ****P* < .001
Indirect effect: *b* = 0.58 (0.07), *P* < .001, 95% CI = [0.45, 0.72]

Table S22: Regression results for mediation model: Intuitive task approach.

	Intuitive task approach	Analytical priority lay belief	Intuitive task approach
(Intercept)	4.101***	5.570***	4.101***
	(0.063)	(0.060)	(0.060)
Condition	–0.745***	1.611***	–0.207
	(0.127)	(0.119)	(0.140)
Analytical priority lay belief			–0.334***
			(0.045)
R^2	0.064	0.266	0.157
Adj. R^2	0.062	0.264	0.153
Number obs.	507	507	507

Note: Unstandardized regression coefficients are displayed, with standard errors in parentheses.
**P* < .05, ***P* < .01, ****P* < .001
Indirect effect: *b* = −0.54 (0.09), *P* < .001, 95% CI = [−0.71, −0.38]

Table S23: Regression results for mediation model: Reliance cognition vs. affect.

	Reliance cognition vs. affect	Analytical priority lay belief	Reliance cognition vs. affect
(Intercept)	2.711***	5.570***	2.711***
	(0.066)	(0.060)	(0.057)
Condition	–0.998***	1.611***	–0.095
	(0.131)	(0.119)	(0.132)
Analytical priority lay belief			–0.561***
			(0.042)
R^2	0.103	0.266	0.335
Adj. R^2	0.101	0.264	0.333
Number obs.	507	507	507

Note: Unstandardized regression coefficients are displayed, with standard errors in parentheses.
P < .05, **P* < .01, ***P* < .001
Indirect effect: *b* = −0.90 (0.11), *P* < .001, 95% CI = [−1.12, −0.70]

f. Moderation by need for cognition

Table S24: Regression results for moderation models: Condition and need for cognition.

Dependent variable	Predictor	*df*	*b*	*t*	*P*
Analytical	Condition	503	0.26	6.00	< .001
	Need for cognition	503	0.14	3.29	.001
	Interaction	503	0.01	0.23	.819
Intuitive	Condition	503	−0.25	−5.86	< .001
	Need for cognition	503	−0.10	−2.44	.015
	Interaction	503	−0.03	−0.58	.560
Reliance cognition vs. affect	Condition	503	−0.32	−7.59	< .001
	Need for cognition	503	−0.12	−2.83	.005
	Interaction	503	−0.03	−0.60	.549

Note: Condition: Human = 0, AI = 1
Need for cognition mean-centered.

Table S25: Regression results for moderation models: Condition and faith in intuition.

Dependent variable	Predictor	*df*	*b*	*t*	*P*
Analytical	Condition	503	0.26	6.00	< .001
	Faith in intuition	503	−0.00	−0.05	.959
	Interaction	503	−0.02	−0.40	.693
Intuitive	Condition	503	−0.23	−5.83	< .001
	Faith in intuition	503	0.41	10.36	< .001
	Interaction	503	0.02	0.61	.544
Reliance cognition vs. affect	Condition	503	−0.31	−7.47	< .001
	Faith in intuition	503	0.14	3.33	.001
	Interaction	503	−0.08	−1.80	.072

Note: Condition: Human = 0, AI = 1
Faith in intuition mean-centered

Table S26: Regression results for moderation models: Condition and application experience.

Dependent variable	Predictor	*df*	*b*	*t*	*P*
Analytical	Condition	503	0.26	6.08	< .001
	Experience	503	0.01	0.29	.773
	Interaction	503	−0.10	−2.26	.024
Intuitive	Condition	503	−0.25	−5.85	< .001
	Experience	503	0.05	1.18	.238
	Interaction	503	−0.02	−0.46	.647
Reliance cognition vs. affect	Condition	503	−0.32	−7.63	< .001
	Experience	503	0.01	0.35	.729
	Interaction	503	0.02	0.43	.670

Note: Condition: Human = 0, AI = 1
Experience mean-centered

g. **Key results without exclusions**

Table S27: Results for independent samples t-tests on key dependent variables and mediator.

Dependent Variable	Comparison	*df*	*t*	*P*	*d*	95% CI
Analytical task approach	AI–human	526	5.95	< .001	0.52	[0.33, 0.68]
Intuitive task approach	AI–human	526	−5.84	< .001	−0.51	[−0.70, −0.34]
Affect vs. cognition	AI–human	526	−7.56	< .001	−0.66	[−0.83, −0.48]
Analytical priority lay belief	AI–human	526	13.67	< .001	1.19	[1.01, 1.39]

Supplementary Information G: Conceptual Replication Study 1: Algorithm Framing

This study replicated Study 1 using an “algorithm” instead of “AI” manipulation and a job application instead of a college application context.

a. Scenario description

You applied at Neosa Inc. for an assistant manager position.
Neosa Inc. invited you to an online assessment where you are evaluated on different tasks.
Right at the beginning of the assessment, Neosa Inc. informs you:
Your performance today will be evaluated by a person [algorithm].
It is solely this person [algorithm] that will monitor and assess your performance in the process and examine your responses. The person's [algorithm’s] performance review decides whether you proceed to a next round.

With this information at hand, how would you approach this assessment?
Please indicate whether you would rather focus on the analytical or intuitive side of yours by responding to all items below.

b. Measures

See SI-E

c. Descriptive statistics

Table S28: Descriptive statistics for key variables.

Variable	Frequency (%) / means (SD)
Gender	
Female	245.00 (46.85%)
Male	267.00 (51.05%)
Diverse	9.00 (1.72%)
Not disclosed	2.00 (0.38%)
Age (years)	40.18 (13.57)
Analytical task approach	5.75 (1.01)
Intuitive task approach	3.95 (1.49)
Reliance cognition vs. affect	2.52 (1.42)
Analytical priority lay belief	5.25 (1.68)
Job application experience	4.97 (3.88)
Need for cognition	4.64 (0.89)
Faith in intuition	3.43 (0.83)
Education	
Less than high school	5.00 (0.96%)
Master	81.00 (15.49%)
Bachelor	227.00 (43.40%)
Other	41.00 (7.84%)
High school	149.00 (28.49%)
Doctorate	20.00 (3.82%)
Occupation	
Unemployed	60.00 (11.47%)
Retired	21.00 (4.02%)
Self-employed	76.00 (14.53%)
Employed	335.00 (64.05%)
Student	16.00 (3.06%)
Other	15.00 (2.87%)
Approved Prolific Tasks	1,885.95 (1,526.11)
Unknown	1

Variable	Frequency (%) / means (SD)
Ethnicity	
Asian	63.00 (12.07%)
Black	61.00 (11.69%)
No Consent	2.00 (0.38%)
Expired data	17.00 (3.26%)
Mixed	30.00 (5.75%)
Other	20.00 (3.83%)
White	329.00 (63.03%)
Unknown	1

N = 523

d. Task approach results

Table S29: Results for independent samples t-tests on key dependent variables.

Dependent variable	Comparison	*df*	*t*	*P*	*d*	95% CI
Analytical task approach	Algorithm–human	521	4.02	< .001	0.35	[0.18, 0.53]
Intuitive task approach	Algorithm–human	521	−2.56	.011	−0.22	[−0.40, −0.05]
Reliance cognition vs. affect	Algorithm–human	521	−3.15	.002	−0.28	[−0.47, −0.10]

Table S30: Results for ANCOVA on key dependent variables, including covariates age, gender, education, need for cognition, ethnicity, and approved Prolific tasks*.

Dependent variable	Comparison	*df*	*t*	*P*	*d*	95% CI
Analytical task approach	Algorithm–human	514	4.30	< .001	0.35	[0.18, 0.53]
Intuitive task approach	Algorithm–human	514	−3.15	.002	−0.22	[−0.40, −0.05]
Reliance cognition vs. affect	Algorithm–human	514	−3.53	< .001	−0.28	[−0.47, −0.10]

*Faith in intuition, experience, and occupation differed between conditions.

e. Analytical priority lay belief results

Table S31: Results for independent samples t-tests on mediator.

Dependent variable	Comparison	*df*	*t*	*P*	*d*	95% CI
Analytical priority lay belief	Algorithm–human	521	19.24	< .001	1.68	[1.44, 1.93]

Table S32: Regression results for mediation model: Analytical task approach.

	Analytical task approach	Analytical priority lay belief	Analytical task approach
(Intercept)	5.746***	5.250***	5.746***
	(0.044)	(0.056)	(0.041)
Condition	0.350***	2.162***	–0.202
	(0.087)	(0.112)	(0.108)
Analytical priority lay belief			0.255***
			(0.032)
R^2	0.030	0.416	0.135
Adj. R^2	0.028	0.414	0.132
Number obs.	523	523	523

Note: Unstandardized regression coefficients are displayed, with standard errors in parentheses.
*$P < .05$, **$P < .01$, ***$P < .001$

Indirect effect: *b* = 0.55 (0.08), *P* < .001, 95% CI = [0.40, 0.72]

Table S33: Regression results for mediation model: Intuitive task approach.

	Intuitive task approach	Analytical priority lay belief	Intuitive task approach
(Intercept)	3.945***	5.250***	3.945***
	(0.065)	(0.056)	(0.062)
Condition	–0.330*	2.162***	0.333*
	(0.129)	(0.112)	(0.163)
Analytical priority lay belief			–0.307***
			(0.049)
R^2	0.012	0.416	0.082
Adj. R^2	0.010	0.414	0.079
Number obs.	523	523	523

Note: Unstandardized regression coefficients are displayed, with standard errors in parentheses.
**P* < .05, ***P* < .01, ****P* < .001
Indirect effect: *b* = −0.66 (0.11), *P* < .001, 95% CI = [−0.89, −0.45]

Table S34: Regression results for mediation model: Reliance cognition vs. affect.

	Reliance cognition vs. affect	Analytical priority lay belief	Reliance cognition vs. affect
(Intercept)	2.523***	5.250***	2.523***
	(0.062)	(0.056)	(0.057)
Condition	–0.398**	2.162***	0.505***
	(0.123)	(0.112)	(0.149)
Analytical priority lay belief			–0.418***
			(0.045)
R^2	0.020	0.416	0.161
Adj. R^2	0.018	0.414	0.158
Number obs.	523	523	523

Note: Unstandardized regression coefficients are displayed, with standard errors in parentheses.
**P* < .05, ***P* < .01, ****P* < .001
Indirect effect: *b* = −0.90 (0.12), *P* < .001, 95% CI = [−1.6, −0.67]

f. Moderation by need for cognition

Table S35: Regression results for moderation models: Condition and need for cognition.

Dependent variable	Predictor	*df*	*b*	*t*	*P*	95% CI
Analytical	Condition	519	0.18	4.31	< .001	[0.00, 0.06]
	Need for cognition	519	0.17	4.09	< .001	[0.00, 0.06]
	Interaction	519	0.07	1.71	.087	[0.00, 0.02]
Intuitive	Condition	519	−0.12	−2.83	.005	[0.00, 0.04]
	Need for cognition	519	−0.14	−3.21	.001	[0.00, 0.04]
	Interaction	519	−0.02	−0.46	.642	[0.00, 0.00]
Reliance cognition vs. affect	Condition	519	−0.15	−3.35	.001	[0.00, 0.05]
	Need for cognition	519	−0.11	−2.45	.015	[0.00, 0.03]
	Interaction	519	−0.03	−0.79	.432	[0.00, 0.01]

Note: Condition: Human = 0, algorithm = 1
Need for cognition mean-centered.

Faith in intuition differed between conditions; thus, we do not explore the moderation.

g. Key results without exclusions

Table S36: Results for independent samples t-tests on key dependent variables and mediator.

Dependent variable	Comparison	*df*	*t*	*P*	*d*	95% CI
Analytical task approach	Algorithm–human	525	4.09	< .001	0.36	[0.16, 0.53]
Intuitive task approach	Algorithm–human	525	−2.60	.010	−0.23	[−0.40, −0.06]
Reliance cognition vs. affect	Algorithm–human	525	−3.26	.001	−0.28	[−0.46, −0.10]
Analytical priority lay belief	Algorithm–human	525	19.44	< .001	1.69	[1.43, 1.92]

Table S37: Regression results for mediation model: Analytical task approach.

	Analytical task approach	Analytical priority lay belief	Analytical task approach
(Intercept)	5.746***	5.252***	5.746***
	(0.043)	(0.056)	(0.041)
Condition	0.353***	2.168***	–0.200
	(0.087)	(0.112)	(0.107)
Analytical priority lay belief			0.256***
			(0.032)
R^2	0.031	0.419	0.136
Adj. R^2	0.029	0.417	0.133
Number obs.	527	527	527

Note: Unstandardized regression coefficients are displayed, with standard errors in parentheses.
*$P < .05$, **$P < .01$, ***$P < .001$
Indirect effect: $b = 0.55$ (0.08), $P < .001$, 95% CI = [0.40, 0.72]

Table S38: Regression results for mediation model: Intuitive task approach.

	Intuitive task approach	Analytical priority lay belief	Intuitive task approach
(Intercept)	3.950***	5.252***	3.950***
	(0.064)	(0.056)	(0.062)
Condition	–0.335**	2.168***	0.329*
	(0.129)	(0.112)	(0.163)
Analytical priority lay belief			–0.306***
			(0.049)
R^2	0.013	0.419	0.082
Adj. R^2	0.011	0.417	0.079
Number obs.	527	527	527

Note: Unstandardized regression coefficients are displayed, with standard errors in parentheses.
*$P < .05$, **$P < .01$, ***$P < .001$
Indirect effect: $b = −0.66$ (0.11), $P < .001$, 95% CI = [−0.89, −0.45]

Table S39: Regression results for mediation model: Reliance cognition vs. affect.

	Reliance cognition vs. affect	Analytical priority lay belief	Reliance cognition vs. affect
(Intercept)	2.524***	5.262***	2.524***
	(0.062)	(0.056)	(0.057)
Condition	−0.392**	2.162***	0.512***
	(0.123)	(0.111)	(0.150)
Analytical priority lay belief			−0.418***
			(0.045)
R^2	.019	.418	.159
Adj. R^2	.017	.417	.156
Number obs.	527	527	527

Note: Unstandardized regression coefficients are displayed, with standard errors in parentheses.
$^*P < .05$, $^{**}P < .01$, $^{***}P < .001$
Indirect effect: $b = -0.90$ (0.13), $P < .001$, 95% CI = [−1.15, −0.67]

Supplementary Information H: Conceptual Replication Study 1: Within-subjects Design

This study proceeded in a similar context as the prior study but as a within-subjects design. Every participant imagined being assessed by both an AI and a human.

a. **Scenario description**

Put yourself in the following scenario:

You are applying for an assistant manager position at the company NEOSA Inc.
Here is some information on NEOSA Inc. and the application process:

- NEOSA Inc. operates a diversified business portfolio and attracts employees with diverse skills and backgrounds.
- NEOSA Inc. wants to get to know you as a person and test your decision-making.
- NEOSA Inc. invited you to an **online assessment** where you are evaluated on different tasks.
- **The application will proceed in two rounds**.

Next, you will imagine to enter the first round of assessment.

<Page break>

Right at the beginning of this round's assessment, Neosa Inc. informs you:
Your performance today will be evaluated by a person [an artificial intelligence].
It is solely this person [AI] that will monitor and assess your performance in the process and examine your responses. The person's [AI's] performance review decides whether you proceed to a next round.

With this information at hand, how would you approach this assessment?
Please indicate whether you would rather focus on the analytical or intuitive side of yours by responding to all items below.

b. **Measures**

See SI-E

c. **Descriptive statistics**

Table S40: Descriptive statistics.

Variable	Frequency (%) / means (SD)
Gender	
Female	61.54%
Male	37.44%
Diverse	1.03%
Age (years)	37.49 (12.51)
Analytical task approach	5.67 (0.96)
Intuitive task approach	4.34 (1.45)
AI involvement	4.14 (0.78)
Education	
Less than high school	1.54%
Master	14.36%
Bachelor	44.62%
Other	11.28%
High school	26.67%
Doctorate	1.54%
Occupation	
Unemployed	8.21%

Variable	Frequency (%) / means (SD)
Retired	5.13%
Self-employed	9.74%
Employed	65.64%
Student	7.18%
Other	4.10%
Approved Prolific Tasks	842.43 (1,277.15)
Ethnicity	
Asian	18.46%
Black	11.28%
Expired data	1.03%
Mixed	6.15%
Other	2.05%
White	61.03%

N = 195

d. **Task approach results**

Table S41: Results for dependent samples t-tests on key dependent variables.

Dependent variable	Comparison	*df*	*t*	*P*	*d*	95% CI
Analytical task approach	AI–human	194	6.90	< .001	0.49	[0.35, 0.62]
Intuitive task approach	AI–human	194	−8.54	< .001	−0.61	[−0.73, −0.45]

e. **Between-subjects comparison**

Table S42: Results for independent samples t-tests on key dependent variables.

Dependent variable	Comparison	*df*	*t*	*P*	*d*	95% CI
Analytical task approach	AI–human	193	5.00	< .001	0.72	[0.42, 1.02]
Intuitive task approach	AI–human	193	−3.16	.002	−0.45	[−0.76, −0.16]

f. **Key results without exclusions**

Table S43: Results for dependent samples t-tests on key dependent variables.

Dependent variable	Comparison	*df*	*t*	*P*	*d*	95% CI
Analytical task approach	AI–human	199	6.73	< .001	0.48	[0.33, 0.60]
Intuitive task approach	AI–human	199	−8.36	< .001	−0.59	[−0.73, −0.44]

Supplementary Information I: Conceptual Replication Study 1: Algorithm Framing and Within-subjects Design

In comparison to the prior study, this replication study changed the framing from “AI” to “algorithm.”

a. **Scenario description**

Put yourself in the following scenario:

You are applying for an assistant manager position at the company NEOSA Inc.
Here is some information on NEOSA Inc. and the application process:

- NEOSA Inc. operates a diversified business portfolio and attracts employees with diverse skills and backgrounds.
- NEOSA Inc. wants to get to know you as a person and test your decision-making.
- NEOSA Inc. invited you to an **online assessment** where you are evaluated on different tasks.
- **The application will proceed in two rounds**.

Next, you will imagine to enter the first round of assessment.

<Page break>

Right at the beginning of this round’s assessment, Neosa Inc. informs you:
Your performance today will be evaluated by a person [an algorithm].
It is solely this person [algorithm] that will monitor and assess your performance in the process and examine your responses. The person's [algorithm’s] performance review decides whether you proceed to a next round.

With this information at hand, how would you approach this assessment?
Please indicate whether you would rather focus on the analytical or intuitive side of yours by responding to all items below.

b. **Measures**

See SI-E

c. **Descriptive statistics**

Table S44: Descriptive statistics for key variables.

Variable	Frequency (%) / means (SD)
Gender	
Female	56.28%
Male	41.71%
Diverse	1.51%
Not disclosed	0.50%
Age (years)	39.91 (13.18)
Analytical task approach	5.68 (1.08)
Intuitive task approach	4.15 (1.43)
AI involvement	3.99 (0.80)
Education	
Less than high school	2.51%
Master	14.07%
Bachelor	43.72%
Other	6.53%
High school	31.66%

Variable	Frequency (%) / means (SD)
Doctorate	1.51%
Occupation	
Unemployed	9.55%
Retired	5.53%
Self-employed	9.55%
Employed	68.84%
Student	4.02%
Other	2.51%
Approved Prolific tasks	1,693.95 (1,793.47)
Ethnicity	
Asian	10.55%
Black	14.07%
No consent	0.50%
Expired data	4.52%
Mixed	4.52%
Other	4.02%
White	61.81%

N = 199

d. **Task approach results**

Table S45: Results for dependent samples t-tests on key dependent variables.

Dependent variable	Comparison	*df*	*t*	*P*	*d*	95% CI
Analytical task approach	Algorithm–human	198	6.83	< .001	0.48	[0.34, 0.60]
Intuitive task approach	Algorithm–human	198	−9.76	< .001	−0.69	[−0.81, −0.58]

e. **Between-subjects comparison**

Table S46: Results for independent samples t-tests on key dependent variables.

Dependent variable	Comparison	*df*	*t*	*P*	*d*	95% CI
Analytical task approach	Algorithm–human	197	2.82	.005	0.40	[0.11, 0.65]
Intuitive task approach	Algorithm–human	197	−3.89	< .001	−0.55	[−0.81, −0.24]

f. **Key results without exclusions**

Table S47: Results for dependent samples t-tests on key dependent variables.

Dependent variable	Comparison	*df*	*t*	*P*	*d*	95% CI
Analytical task approach	Algorithm–human	201	6.74	< .001	0.47	[0.34, 0.60]
Intuitive task approach	Algorithm–human	201	−9.74	< .001	−0.69	[−0.79, −0.57]

Supplementary Information J: Study 2

a. **Measures**

Stage 1

AI receptiveness

- AI literacy (28)
  - Recognizing AI:
    1. Which of the following is NOT powered by AI?
    a) Self-driving cars
    b) Google's search algorithm
    c) A basic calculator (correct)
    d) Chatbots
  - Understanding Intelligence:
    2. Which form of intelligence involves emotional understanding and social skills?
    a) Machine Intelligence
    b) Human Intelligence (correct)
    c) Animal Intelligence
    d) Artificial General Intelligence
  - Interdisciplinarity:
    3. Which of the following fields contributes to the development of artificial intelligence?
    a) Computer science
    b) Mathematics
    c) Psychology
    d) All of the above (correct)
  - General vs Narrow AI:
    4. What is the term for AI systems that can perform any intellectual task that a human can?
    a) Narrow AI
    b) General AI (correct)
    c) Weak AI
    d) Strong AI
  - AI's Strengths and Weaknesses:
    5. In which area does AI typically excel?
    a) Emotional understanding
    b) Pattern recognition (correct)
    c) Moral reasoning
    d) Creativity
  - Imagine Future AI:
    6. Which of the following is NOT a likely future application of AI?
    a) Personalized healthcare
    b) Emotional robots
    c) Time travel (correct)
    d) Sustainable energy management
  - Representations:
    7. What is a common form of knowledge representation in AI?
    a) Neural networks (correct)
    b) Waterfall model
    c) Agile methodology
    d) SWOT analysis
  - Decision-Making:
    8. Which algorithmic approach is commonly used for decision-making in AI?
    a) Dijkstra's algorithm

b) Depth-first search
c) Decision trees (correct)
d) Fourier Transform

- Machine Learning Steps:
  9. What is the first step in a typical machine learning process?
  a) Data collection (correct)
  b) Model selection
  c) Prediction
  d) Model evaluation
- Human Role in AI:
  10. Who is primarily responsible for the ethical considerations of an AI system?
  a) The AI system itself
  b) Data providers
  c) Human developers (correct)
  d) End-users
- Data Literacy:
  11. Which of the following is an example of metadata?
  a) A spreadsheet of numbers
  b) Column headers in a table (correct)
  c) A chart visualization
  d) Raw sensor data
- Learning from Data:
  12. How do supervised machine learning algorithms learn?
  a) From labeled data (correct)
  b) From rewards and punishments
  c) By observing human behavior
  d) From intrinsic motivation
- Critically Interpreting Data:
  13. Why can data not be taken at face value?
  a) It is always inaccurate
  b) It requires interpretation (correct)
  c) It is self-explanatory
  d) It is always biased
- Action & Reaction:
  14. How can an AI system interact with the physical world?
  a) By planning movements
  b) By reacting to sensor inputs
  c) By actuating motors
  d) All of the above (correct)
- Sensors:
  15. Which of the following sensors allow an AI system to perceive the world?
  a) Cameras
  b) Microphones
  c) Thermometers
  d) All of the above (correct)
- Ethics:
  16. Which is a key ethical issue surrounding AI?
  a) Algorithmic efficiency
  b) CPU usage
  c) Privacy (correct)
  d) Code readability
- Programmability:
  17. Which statement best describes the programmability of AI systems?
  a) They cannot be programmed by humans
  b) They program themselves

c) They are programmed using data
d) They are programmed by computer code (correct)

- AI attitude (29) *(1 = "strongly disagree" to 7 = "strongly agree")*
    - AI will make this world a better place.
    - I have strong negative emotions about AI. (reverse-coded)
    - I want to use technologies that rely on AI.
    - AI has more disadvantages than advantages. (reverse-coded)
    - I look forward to future AI developments.
    - AI offers solutions to many world problems.
    - I prefer technologies that do not feature AI. (reverse-coded)
    - I am afraid of AI. (reverse-coded)
    - I would rather choose a technology with AI than one without it.
    - AI creates problems rather than solving them. (reverse-coded)
    - When I think about AI, I have mostly positive feelings.
    - I would rather avoid technologies that are based on AI. (reverse-coded)

- AI subjective understanding (30)
    - To what extent do you understand how an AI performs the tasks it is assigned to? *(1 = "not at all" to 7 = "completely")*

- AI familiarity
    - How often a week do you use AI or tools that use AI as part of your work or private life? *(1 = "never" to 5 = "multiple times every day")*

Job application context

- Future employment anxiety (31) *(1 = "strongly disagree" to 7 = "strongly agree")*
    - I often feel insecure about how my career will develop in the next year or so.
    - Advancements in technology and automation make me concerned about my future job security.
    - I feel pressure to continuously improve my employability.
    - I often worry about my ability to find a job in the future.
    - The uncertainty of the job market makes me feel stressed.
    - I worry that my skills may not be enough for future employment.

- Application experience
    - How much experience do you have with job applications? *(1 = "no experience at all" to 7 = "a lot of experience")*

Personality

- Fear of negative evaluation (32) *(1 = "not at all characteristic of me" to 7 = "extremely characteristic of me")*
    - I worry about what other people will think of me even when I know it doesn't make any difference.
    - It bothers me when people form an unfavorable impression of me.
    - I am frequently afraid of other people noticing my shortcomings.
    - I worry about what kind of impression I make on people.
    - I am afraid that others will not approve of me.
    - I am concerned about other people's opinions of me.
    - When I am talking to someone, I worry about what they may be thinking about me.
    - I am usually worried about what kind of impression I make.
    - If I know someone is judging me, it tends to bother me.
    - Sometimes I think I am too concerned with what other people think of me.

- I often worry that I will say or do wrong things.
- Other people's opinions of me do not bother me.

- Big Five Inventory (33) (1 = "strongly disagree" to 7 = "strongly agree")
  "I see myself as someone who..."
  - ... is reserved
  - ... is outgoing, sociable
  - ... is generally trusting
  - ... tends to find fault with others
  - ... does a thorough job
  - ... tends to be lazy
  - ... is relaxed, handles stress well
  - ... gets nervous easily
  - ... has an active imagination
  - ... has few artistic interests

Stage 2

I would rather rely on...
- …"my thoughts" (1) to "my feelings" (7)
- ... "the rational side of me" to "the emotional side of me" (7)
- … "my head" (1) to "my heart" (7)

b. **Descriptive statistics**

Table S48: Descriptive statistics.

Variable	US Census	Study 1 + 2<br>*N* = 1,166[1]	Study 1 only<br>*N* = 320[1]	P^2
Gender				0.47
Female	50.5%	596.00 (51.11%)	154.00 (48.13%)	
Male	49.5%	557.00 (47.77%)	163.00 (50.94%)	
Diverse	-	8.00 (0.69%)	3.00 (0.94%)	
Not disclosed	-	5.00 (0.43%)	0.00 (0.00%)	
Education				0.048
Less than high school		39.00 (3.34%)	11.00 (3.44%)	
Master		208.00 (17.84%)	44.00 (13.75%)	
Bachelor		399.00 (34.22%)	141.00 (44.06%)	
Other		160.00 (13.72%)	39.00 (12.19%)	
High school		323.00 (27.70%)	76.00 (23.75%)	
Doctorate		37.00 (3.17%)	9.00 (2.81%)	
Occupation				0.14
Unemployed		123.00 (10.55%)	30.00 (9.38%)	
Retired		162.00 (13.89%)	30.00 (9.38%)	
Self-employed		134.00 (11.49%)	35.00 (10.94%)	
Employed		645.00 (55.32%)	193.00 (60.31%)	
Student		45.00 (3.86%)	19.00 (5.94%)	
Other		57.00 (4.89%)	13.00 (4.06%)	
Income		86,585.85 (85,104.24)	80,491.92 (73,228.87)	0.24
AI attitude		3.78 (0.47)	3.84 (0.52)	0.031
AI familiarity		2.62 (1.21)	2.77 (1.19)	0.040
Fear of negative evaluation		3.76 (1.40)	3.71 (1.39)	0.54
Future employment anxiety		3.85 (1.71)	3.88 (1.68)	0.78

Variable	US Census	Study 1 + 2<br>*N* = 1,166[1]	Study 1 only<br>*N* = 320[1]	*P*[2]
Extraversion		3.71 (1.44)	3.83 (1.42)	0.17
Openness		5.02 (1.34)	4.94 (1.29)	0.37
Neuroticism		3.68 (1.54)	3.55 (1.63)	0.19
Agreeableness		4.97 (1.30)	4.95 (1.32)	0.78
Conscientiousness		5.41 (1.21)	5.46 (1.23)	0.47
AI literacy		12.15 (2.24)	11.70 (2.84)	0.003
Job application experience		5.09 (1.66)	5.13 (1.65)	0.68
Age	*	45.74 (15.80)	42.82 (16.21)	0.004
AI subjective understanding		3.98 (1.56)	4.22 (1.57)	0.015
Approved Prolific tasks		587.54 (927.73)	548.02 (850.74)	0.49
Unknown		1	2	
Ethnicity				0.003
Asian	6.0%	60.00 (5.15%)	23.00 (7.23%)	
Black	12.4%	125.00 (10.73%)	47.00 (14.78%)	
No consent	-	0.00 (0.00%)	1.00 (0.31%)	
Mixed	-	149.00 (12.79%)	25.00 (7.86%)	
Other	8.4%	80.00 (6.87%)	31.00 (9.75%)	
White	61.6%	751.00 (64.46%)	191.00 (60.06%)	
Unknown	-	1	2	

[1]n (%); Mean (SD)
[2]Pearson's Chi-squared test; Two Sample t-test
* Age is measured in categories and includes under 18 in US Census data

c. **Moderations**

As pre-registered we specified models including each moderator separately as factor, with condition as second factor and their interaction as third factor. The dependent variable was analytical task approach measured as reliance on cognition (lower pole) versus affect (higher pole).

Table S49: Moderation models for covariates.

Effect	*SS*	*df*	*F*	*P*
(Intercept)	5,223.619	1	2,175.015	< .001
Condition	61.472	1	25.596	< .001
Age	9.941	1	4.139	0.042
**Interaction**	**11.324**	**1**	**4.715**	**0.030**
Residuals	2,785.911	1,160		
(Intercept)	2,747.797	1	1,154.200	< .001
Condition	10.208	1	4.288	0.039
Gender	5.943	3	0.832	0.476
Interaction	13.621	3	1.907	0.127
Residuals	2,752.082	1,156		
(Intercept)	4,535.455	1	1,864.373	< .001
Condition	57.982	1	23.835	< .001
Education	2.817	1	1.158	0.282
Interaction	0.018	1	0.007	0.932
Residuals	2,435.130	1,001		
(Intercept)	726.682	1	302.238	< .001
Condition	32.921	1	13.692	< .001
Occupation	21.755	5	1.810	0.108
**Interaction**	**23.846**	**5**	**1.984**	**0.078**

Effect	*SS*	*df*	*F*	*P*
Residuals	2,769.798	1,152		
(Intercept)	5,240.921	1	2,174.052	< .001
Condition	61.764	1	25.621	< .001
Income	1.786	1	0.741	0.390
Interaction	0.149	1	0.062	0.804
Residuals	2,796.377	1,160		
(Intercept)	5,246.360	1	2,178.133	< .001
Condition	62.163	1	25.808	< .001
AI attitude	3.831	1	1.590	0.208
Interaction	3.882	1	1.612	0.205
Residuals	2,794.034	1,160		
(Intercept)	5,235.335	1	2,206.371	< .001
Condition	61.186	1	25.786	< .001
Fear of negative evaluation	40.656	1	17.134	< .001
**Interaction**	**8.010**	**1**	**3.376**	**0.066**
Residuals	2,752.479	1,160		
(Intercept)	5,255.824	1	2,201.131	< .001
Condition	63.807	1	26.722	< .001
Future employment anxiety	21.384	1	8.955	0.003
Interaction	1.793	1	0.751	0.386
Residuals	2,769.829	1,160		
(Intercept)	5,222.516	1	2,179.460	< .001
Condition	59.680	1	24.906	< .001
Extraversion	8.538	1	3.563	0.059
Interaction	0.042	1	0.018	0.894
Residuals	2,779.642	1,160		
(Intercept)	5,241.611	1	2,181.656	< .001
Condition	61.084	1	25.424	< .001
Agreeableness	1.000	1	0.416	0.519
**Interaction**	**8.788**	**1**	**3.658**	**0.056**
Residuals	2,786.997	1,160		
(Intercept)	5,236.360	1	2,199.161	< .001
Condition	61.265	1	25.730	< .001
Conscientiousness	26.565	1	11.157	0.001
Interaction	1.711	1	0.718	0.397
Residuals	2,762.043	1,160		
(Intercept)	5,251.264	1	2,192.409	< .001
Condition	65.680	1	27.421	< .001
Neuroticism	9.010	1	3.762	0.053
Interaction	0.040	1	0.017	0.897
Residuals	2,778.435	1,160		
(Intercept)	5,187.159	1	2,152.298	< .001
Condition	58.925	1	24.450	< .001
AI literacy	0.932	1	0.387	0.534
Interaction	0.137	1	0.057	0.812
Residuals	2,795.665	1,160		
(Intercept)	5,233.137	1	2,212.282	< .001
Condition	60.643	1	25.636	< .001
Job application experience	29.684	1	12.549	< .001
Interaction	0.008	1	0.004	0.952
Residuals	2,743.972	1,160		
(Intercept)	5,237.220	1	2,176.530	< .001
Condition	61.348	1	25.495	< .001
AI familiarity	2.875	1	1.195	0.275

Effect	*SS*	*df*	*F*	*P*
Interaction	0.082	1	0.034	0.854
Residuals	2,791.221	1,160		
(Intercept)	5,241.689	1	2,176.771	< .001
Condition	61.703	1	25.624	< .001
AI subjective understanding	2.015	1	0.837	0.361
Interaction	0.109	1	0.045	0.832
Residuals	2,793.293	1,160		
(Intercept)	5,249.964	1	2,187.335	< .001
Condition	63.444	1	26.433	< .001
Approved Prolific tasks	8.348	1	3.478	0.062
Interaction	0.190	1	0.079	0.778
Residuals	2,781.790	1,159		
(Intercept)	296.626	1	123.278	< .001
Condition	8.563	1	3.559	0.059
Ethnicity	6.722	4	0.698	0.593
Interaction	9.366	4	0.973	0.421
Residuals	2,774.305	1,153		

Note: Numeric covariates are mean-centered.
Openness differed between conditions (*P* < .001); thus, we did not explore its role as moderator or covariate.

Figure S3: Interaction plot age and condition on reliance on cognition versus affect.

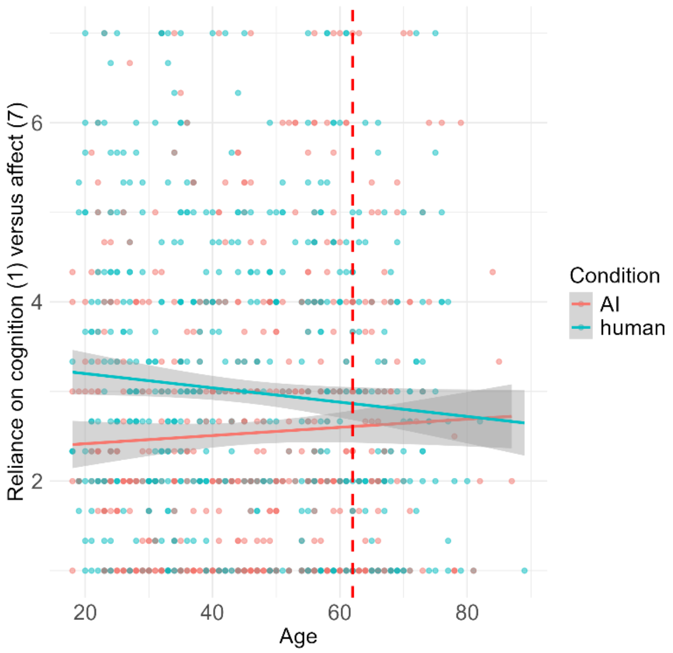

Note: When age is outside the interval [61.99, 428.42], the slope of condition is *P* < .05. The range of observed values of age is 18 to 89 years.

Figure S4: Interaction plot fear of negative evaluation and condition on reliance on cognition versus affect.

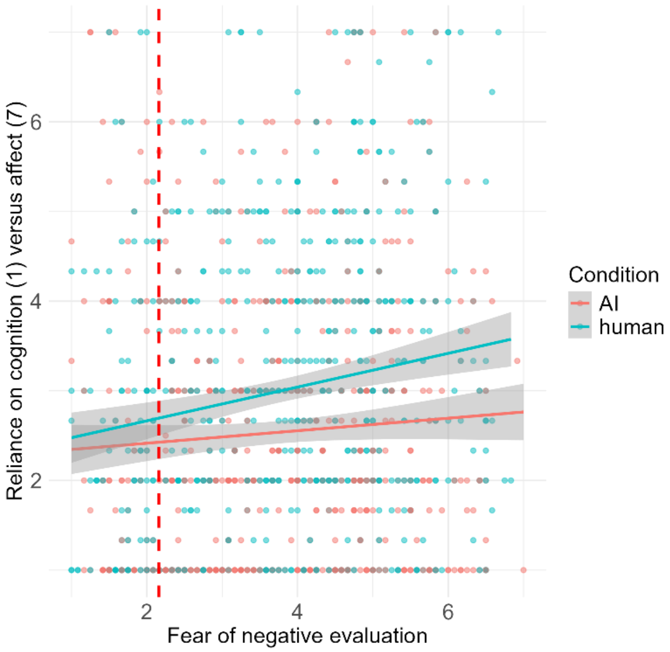

When fear of negative evaluation is inside the interval [2.16, 60.47], the slope of condition is $P < .05$. The range of observed values of fear of negative evaluation is 1 to 7.

Figure S5: Interaction plot agreeableness and condition on reliance on affect over cognition.

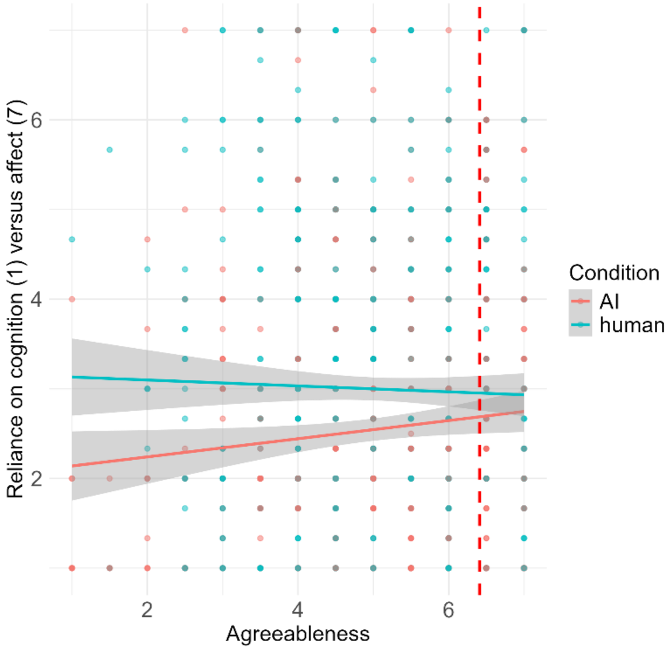

When agreeableness is inside the interval [−126.59, 6.41], the slope of condition is $P < .05$. The range of observed values of agreeableness is 1 to 7.

Figure S6: Interaction plot: occupation and condition on reliance on affect over cognition.

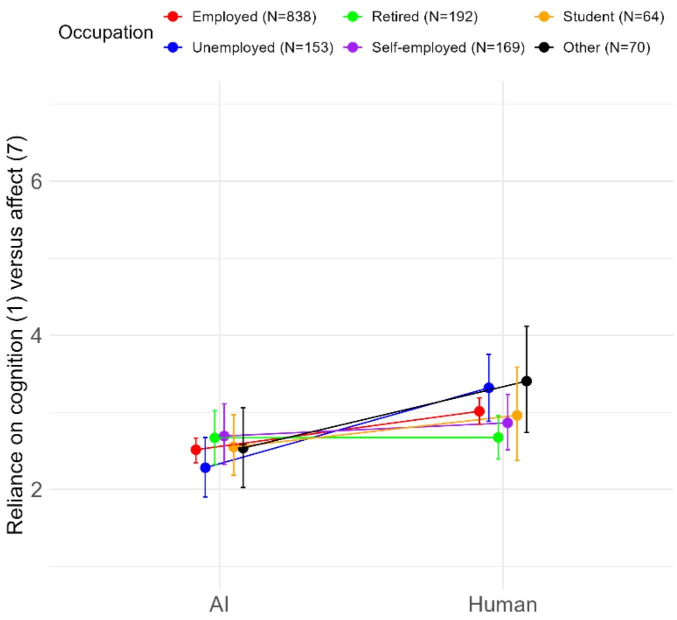

d. **Key results without exclusions**

Table S50: Independent-samples t-test without exclusions.

Dependent variable	Comparison	*df*	*t*	*P*	*d*	95% CI
Reliance on cognition versus affect	AI–human	1,179	−5.05	< .001	−0.29	[−0.41, −0.18]

Supplementary Information K: Study 3

a. **Measures**

Secondary measures

With this information at hand, how would you approach this assessment?

All items measured on seven-point Likert scales from 1 = "strongly disagree" to 7 = "strongly agree."

- Creativity:
  - I would generate multiple original solutions to these tasks.
  - I would approach these tasks with innovative thinking.
  - I would seek out-of-the-box solutions for these tasks.
  - I would experiment with unconventional methods to complete these tasks.
  - I would embrace imaginative ideas when tackling these tasks.
- Effort:
  - I would dedicate significant time to ensure these tasks are completed thoroughly.
  - I would persist through challenges to accomplish these tasks.
  - I would invest considerable energy into performing these tasks well.
  - I would maintain focus and diligence while working on these tasks.
  - I would strive to give my best effort in completing these tasks.
- Ethical and social considerations:
  - I would ensure my approach aligns with ethical standards.
  - I would consider the potential impact of my actions on others.
  - I would prioritize fairness and integrity in completing these tasks.
  - I would reflect on the moral implications before deciding on a course of action.
  - I would seek to uphold social responsibility in my approach to these tasks.
- Risk-taking:
  - I would embrace uncertainty to achieve the desired outcome.
  - I would be willing to take risks to complete these tasks.
  - I would prefer bold actions over cautious ones in tackling these tasks.
  - I would choose innovative methods, even if they involve some risk.
  - I would feel comfortable making decisions under uncertainty for these tasks.

b. **Descriptive statistics**

Table S51: Descriptive statistics.

Variable	Study 1 + 2<br>*N* = 1,138[1]	Study 1 only<br>*N* = 347[1]	*P*[2]
Age	40.63 (21.52)	36.52 (13.92)	< .001
Gender			0.10
Female	657.00 (57.73%)	209.00 (60.23%)	
Male	462.00 (40.60%)	129.00 (37.18%)	
Diverse	15.00 (1.32%)	4.00 (1.15%)	
Not disclosed	4.00 (0.35%)	5.00 (1.44%)	
Education			0.19
Less than high school	13.00 (1.14%)	1.00 (0.29%)	
Master	220.00 (19.33%)	60.00 (17.29%)	
Bachelor	424.00 (37.26%)	137.00 (39.48%)	
Other	98.00 (8.61%)	19.00 (5.48%)	
High school	327.00 (28.73%)	111.00 (31.99%)	
Doctorate	56.00 (4.92%)	19.00 (5.48%)	
Occupation			0.051
Unemployed	105.00 (9.23%)	23.00 (6.63%)	
Retired	64.00 (5.62%)	12.00 (3.46%)	

Variable	Study 1 + 2 N = 1,138[1]	Study 1 only N = 347[1]	P^2
Self-employed	136.00 (11.95%)	39.00 (11.24%)	
Employed	721.00 (63.36%)	221.00 (63.69%)	
Student	90.00 (7.91%)	42.00 (12.10%)	
Other	22.00 (1.93%)	10.00 (2.88%)	
Approved Prolific tasks	574.75 (911.50)	523.87 (834.48)	0.35
Ethnicity			0.19
Asian	66.00 (5.80%)	27.00 (7.78%)	
Black	143.00 (12.57%)	52.00 (14.99%)	
Expired data	10.00 (0.88%)	3.00 (0.86%)	
Mixed	70.00 (6.15%)	31.00 (8.93%)	
Other	43.00 (3.78%)	13.00 (3.75%)	
Prefer not to say	1.00 (0.09%)	1.00 (0.29%)	
White	805.00 (70.74%)	220.00 (63.40%)	

[1]Mean (SD); n (%)
[2]Two Sample t-test; Pearson's Chi-squared test

Table S52: Descriptive statistics for dependent variables in stage 1.

Dependent variable	AI N = 372[1]	Human N = 368[1]	No N = 398[1]
Analytical task approach	5.85 (0.85)	5.60 (1.01)	5.42 (1.02)
Ethical and social considerations	5.50 (1.17)	5.86 (0.91)	5.64 (1.04)
Creativity	4.55 (1.35)	5.08 (1.05)	4.99 (1.06)
Risk-taking	4.18 (1.39)	4.49 (1.20)	4.42 (1.21)
Effort	6.12 (0.93)	6.19 (0.80)	5.96 (0.89)

[1]Mean (SD); only including participants who returned to stage 2

Table S53: Descriptive statistics for dependent variables in stage 2.

Characteristic	AI N = 579[1]	Human N = 559[1]
Analytical task approach	5.91 (0.88)	5.53 (1.10)
Ethical and social considerations	5.54 (1.13)	5.87 (0.96)
Creativity	4.46 (1.39)	5.05 (1.16)
Risk-taking	4.09 (1.39)	4.54 (1.28)
Effort	6.15 (0.89)	6.26 (0.78)

[1]Mean (SD)

c. **Key results robustness checks**

Table S54: Between-subjects contrasts between conditions in stage 1.

Dependent Variable	Comparison	*df*	*t*	*p*	*d*	95% CI
Analytical task approach	AI–human	1,135	3.49	< .001	0.26	[0.13, 0.41]
	AI–no	1,135	6.20	< .001	0.45	[0.31, 0.60]
	Human–no	1,135	2.63	.009	0.19	[0.04, 0.35]
Ethical and social considerations	AI–human	1,135	−4.68	< .001	−0.34	[−0.48, −0.19]
	AI–no	1,135	−1.82	.070	−0.13	[−0.28, 0.01]
	Human–no	1,135	2.94	.003	0.21	[0.07, 0.35]
Creativity	AI–human	1,135	−6.16	< .001	−0.45	[−0.60, −0.30]
	AI–no	1,135	−5.30	< .001	−0.38	[−0.54, −0.24]
	Human–no	1,135	0.98	.327	0.07	[−0.06, 0.20]
Risk−taking	AI–human	1,135	−3.23	.001	−0.24	[−0.39, −0.09]

Dependent Variable	Comparison	*df*	*t*	*p*	*d*	95% CI
	AI–no	1,135	−2.53	.012	−0.18	[−0.32, −0.03]
	Human–no	1,135	0.77	.444	0.06	[−0.08, 0.18]
Effort investment	AI–human	1,135	−1.15	.250	−0.08	[−0.24, 0.06]
	AI–no	1,135	2.48	.013	0.18	[0.03, 0.33]
	Human–no	1,135	3.64	< .001	0.26	[0.12, 0.39]

Note: We include only those participants that returned in stage 2 in this analysis. The effect pattern persists when including all participants.

Table S55: Between-subjects contrasts between conditions in stage 1, including covariates age, gender, education, occupation, approved Prolific tasks, and ethnicity.

Dependent Variable	Comparison	*df*	*t*	*p*	*d*	95% CI
Analytical task approach	AI–human	1,129	3.57	< .001	0.26	[0.11, 0.40]
	AI–no	1,129	6.27	< .001	0.45	[0.31, 0.58]
	Human–no	1,129	2.62	.009	0.19	[0.05, 0.34]
Ethical and social considerations	AI–human	1,129	−4.95	< .001	−0.34	[−0.48, −0.19]
	AI–no	1,129	−2.04	.042	−0.13	[−0.28, 0.01]
	Human–no	1,129	3.01	.003	0.21	[0.08, 0.35]
Creativity	AI–human	1,129	−6.14	< .001	−0.45	[−0.61, −0.32]
	AI–no	1,129	−5.30	< .001	−0.38	[−0.53, −0.23]
	Human–no	1,129	0.96	.338	0.07	[−0.05, 0.20]
Risk−taking	AI–human	1,129	−3.15	.002	−0.24	[−0.38, −0.09]
	AI–no	1,129	−2.51	.012	−0.18	[−0.32, −0.03]
	Human–no	1,129	0.70	.481	0.06	[−0.08, 0.18]
Effort investment	AI–human	1,129	−1.26	.209	−0.08	[−0.22, 0.05]
	AI–no	1,129	2.38	.017	0.18	[0.03, 0.33]
	Human–no	1,129	3.66	< .001	0.26	[0.13, 0.41]

Table S56: Within-subjects contrasts across stages and for all combinations of conditions.

Dependent variable	Group	*t*	*df*	*P*	*d*
Analytical task approach	No, AI	−6.71	204	< .001	−0.47
	No, human	−1.42	192	0.156	−0.10
	Human, human	1.90	171	0.060	0.14
	AI, AI	−0.84	177	0.401	−0.06
	AI, human	5.37	193	< .001	0.39
	Human, AI	−6.07	195	< .001	−0.43
Creativity	No, AI	5.11	204	< .001	0.36
	No, human	−0.25	192	0.803	−0.02
	Human, human	0.95	171	0.342	0.07
	AI, AI	1.10	177	0.273	0.08
	AI, human	−5.62	193	< .001	−0.40
	Human, AI	6.05	195	< .001	0.43
Effort investment	No, AI	−3.93	204	< .001	−0.27
	No, human	−3.21	192	0.002	−0.23
	Human, human	−0.04	171	0.968	0.00
	AI, AI	−1.48	177	0.140	−0.11
	AI, human	−1.58	193	0.116	−0.11
	Human, AI	−0.27	195	0.784	−0.02
Ethical and social considerations	No, AI	−0.41	204	0.682	−0.03
	No, human	−2.55	192	0.012	−0.18
	Human, human	1.54	171	0.126	0.12
	AI, AI	−0.06	177	0.949	0.00
	AI, human	−4.45	193	< .001	−0.32

Dependent variable	Group	*t*	*df*	*P*	*d*
	Human, AI	3.41	195	0.001	0.24
Risk-taking	No, AI	2.57	204	0.011	0.18
	No, human	−1.53	192	0.127	−0.11
	Human, human	−0.34	171	0.735	−0.03
	AI, AI	2.50	177	0.013	0.19
	AI, human	−3.98	193	< .001	−0.29
	Human, AI	4.26	195	< .001	0.30

Note: The within-subjects comparison for analytical task approaches between AI and human reported in the manuscript collapsed the AI, human and human, AI cells.

d. **Selection exploration**

We explored whether AI assessment might affect the selection of candidates. As some participants underwent both AI and human assessment, we could explore whether the same person would be selected under one but not the other assessment type. We pre-registered different thresholds of analytical task approaches based on the Likert-scale values (4, 5, 6) and simulated whether a candidate would be selected, based on indicating an analytical task approach above this value, i.e., an organization might want to hire a moderately to very strong analytical candidate. Per threshold, we report the number of people that would have been selected (i.e., those that meet the threshold) under the respective assessment. Of main interest are cases where the same candidate is selected under AI assessment but not under human assessment (see **bold cells** in tables below). As the tables indicate, the number of people selected only under AI assessment increases with stricter thresholds, so that more than 100 people would only be selected under AI assessment in the case of a high threshold for analytical capabilities.

Table S57: Contingency table for threshold = 4

		Human Assessment
AI Assessment	Human: Not Selected	Human: Selected
AI: Not Selected	1	9
AI: Selected	**36**	344

McNemar's Chi-squared = 15.02, *P* < .001

Table S58: Contingency table for threshold = 5

		Human Assessment
AI Assessment	Human: Not Selected	Human: Selected
AI: Not Selected	27	35
AI: Selected	**99**	229

McNemar's Chi-squared = 29.62, *P* < .001

Table S59: Contingency table for threshold = 6

		Human Assessment
AI Assessment	Human: Not Selected	Human: Selected
AI: Not Selected	186	30
AI: Selected	**106**	68

McNemar's Chi-squared = 41.36, *P* < .001

e. Key results without exclusions

Table S60: Between-subjects contrasts between conditions in stage 1.

Dependent variable	Comparison	*df*	*t*	*p*	*d*	95% CI
Analytical task approach	AI–human	1,162	3.56	< .001	0.26	[0.13, 0.39]
	AI–no	1,162	6.48	< .001	0.46	[0.32, 0.59]
	Human–no	1,162	2.85	.004	0.20	[0.05, 0.35]
Ethical and social considerations	AI–human	1,162	−4.57	< .001	−0.33	[−0.47, −0.19]
	AI–no	1,162	−1.74	.082	−0.12	[−0.27, 0.03]
	Human–no	1,162	2.91	.004	0.21	[0.08, 0.34]
Creativity	AI–human	1,162	−6.16	< .001	−0.45	[−0.59, −0.31]
	AI–no	1,162	−5.27	< .001	−0.38	[−0.52, −0.22]
	Human–no	1,162	1.00	.318	0.07	[−0.06, 0.19]
Risk−taking	AI–human	1,162	−3.28	.001	−0.24	[−0.39, −0.09]
	AI–no	1,162	−2.48	.013	−0.18	[−0.32, −0.03]
	Human–no	1,162	0.85	.394	0.06	[−0.07, 0.19]
Effort investment	AI–human	1,162	−1.03	.302	−0.07	[−0.22, 0.06]
	AI–no	1,162	2.75	.006	0.20	[0.06, 0.34]
	Human–no	1,162	3.79	< .001	0.27	[0.13, 0.40]

Table S61: Within-subjects contrasts across stages and for all combinations of conditions.

Dependent variable	Group	*t*	*df*	*P*	*d*
Analytical task approach	No, AI	−6.89	210	0.000	−0.47
	No, human	−1.60	195	0.111	−0.11
	Human, human	2.10	177	0.037	0.16
	AI, AI	−0.97	179	0.336	−0.07
	AI, human	5.75	200	0.000	0.41
	Human, AI	−6.24	198	0.000	−0.44
Creativity	No, AI	5.20	210	0.000	0.36
	No, human	−0.36	195	0.717	−0.03
	Human, human	0.97	177	0.332	0.07
	AI, AI	1.03	179	0.304	0.08
	AI, human	−5.52	200	0.000	−0.39
	Human, AI	6.16	198	0.000	0.44
Effort investment	No, AI	−3.70	210	0.000	−0.25
	No, human	−3.24	195	0.001	−0.23
	Human, human	0.10	177	0.921	0.01
	AI, AI	−1.39	179	0.167	−0.10
	AI, human	−1.35	200	0.178	−0.10
	Human, AI	−0.32	198	0.749	−0.02
Ethical and social considerations	No, AI	−0.26	210	0.793	−0.02
	No, human	−2.46	195	0.015	−0.18
	Human, human	1.51	177	0.132	0.11
	AI, AI	−0.08	179	0.937	−0.01
	AI, human	−4.14	200	0.000	−0.29
	Human, AI	3.31	198	0.001	0.23
Risk−taking	No, AI	2.44	210	0.016	0.17
	No, human	−1.61	195	0.109	−0.12
	Human, human	−0.13	177	0.898	−0.01
	AI, AI	2.43	179	0.016	0.18
	AI, human	−3.97	200	0.000	−0.28
	Human, AI	4.20	198	0.000	0.30

Supplementary Information L: Study 4

a. **Measures Part 1**

For all professional roles below, please indicate whether you would rather emphasize your analytical or intuitive skills, if you were to apply for the professional role and would like to be hired.

Analytical skills may comprise the ability to dissect problems, as well as systematic and logical thinking.

Intuitive skills may comprise the ability to rely on gut feelings, as well as emotional and experiential thinking.

- Marketing manager
- Customer representative
- Financial manager
- Event planner
- Social worker
- Consultant
- Teacher
- Market researcher
- Accountant
- Nurse
- Psychologist
- Research pool participant (such as Prolific)
- Public relations specialist
- Operations manager
- Data analyst

All measured on seven-point Likert scale from 1 = “analytical skills” to 7 = “intuitive skills.”

b. **Results Part 1**

Figure S7: Intuitive capabilities required for tested professional positions.

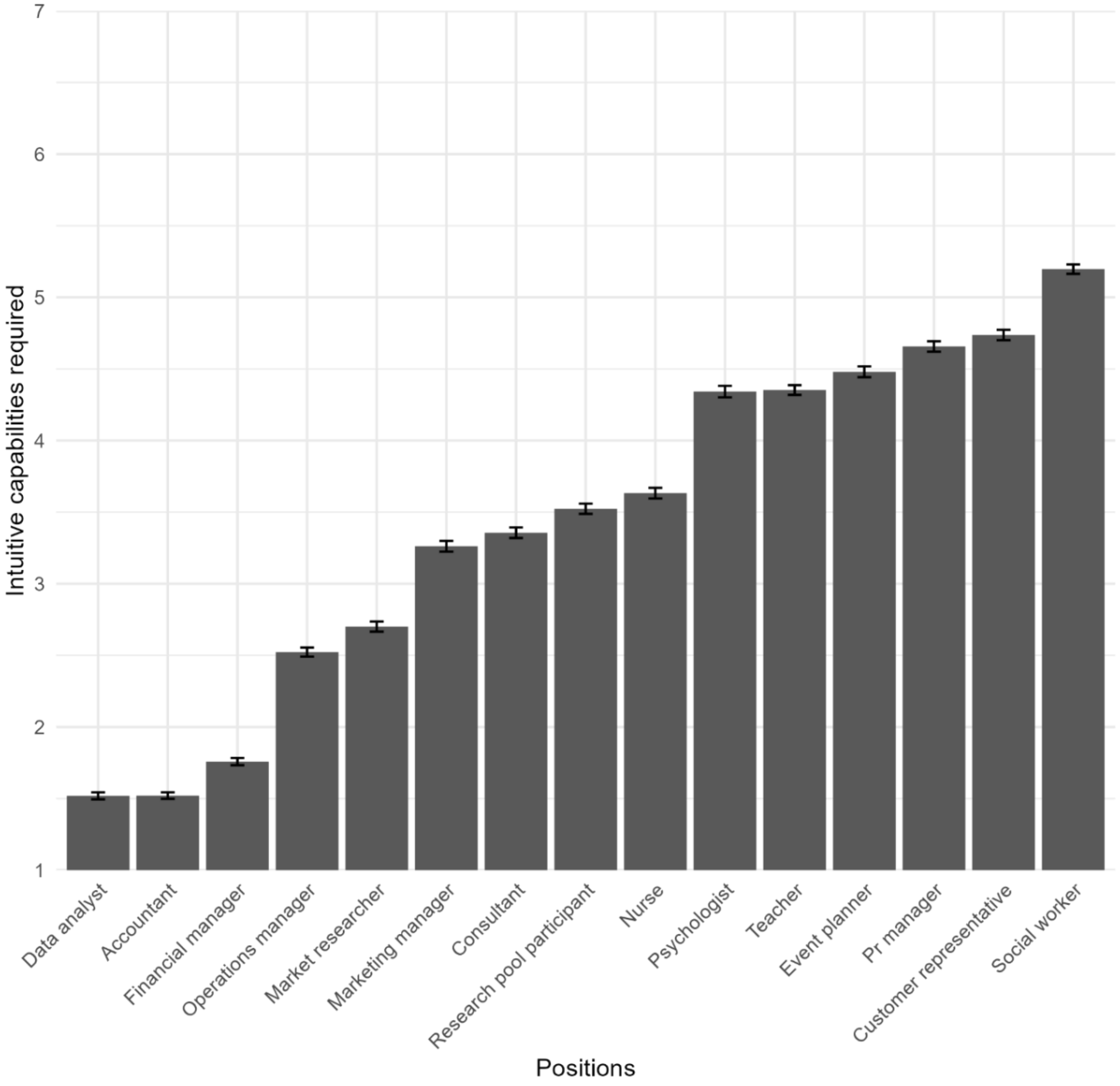

Note: Error bars represent standard errors of the mean.

c. **Descriptives and differences between study parts**

Table S62: Descriptive statistics.

Variable	Parts 1 and 2, N = 1,789[1]	Part 1 only, N = 211[1]	P[2]
Skills required for research pool	3.51 (1.58)	3.72 (1.74)	0.070
Age	40.71 (13.66)	39.46 (13.16)	0.45
Gender			0.11
Female	1,012.00 (56.57%)	103.00 (48.82%)	
Male	733.00 (40.97%)	104.00 (49.29%)	
Diverse	36.00 (2.01%)	4.00 (1.90%)	
Not disclosed	8.00 (0.45%)	0.00 (0.00%)	
Approved Prolific tasks	2,144.29 (2,147.38)	1,800.88 (2,054.56)	< .001
Ethnicity			< .001
Asian	241.00 (13.47%)	23.00 (10.90%)	
Black	227.00 (12.69%)	43.00 (20.38%)	
No consent	0.00 (0.00%)	1.00 (0.47%)	
Expired data	11.00 (0.61%)	0.00 (0.00%)	
Mixed	125.00 (6.99%)	16.00 (7.58%)	
Other	69.00 (3.86%)	6.00 (2.84%)	
White	1,116.00 (62.38%)	122.00 (57.82%)	

[1]Mean (SD); n (%)

[2]Independent samples t-test; Pearson's chi-squared test

Note: Age had one outlier that we removed from the analysis.

d. **Measures Part 2**

A few important remarks about how you will be evaluated.

A qualified person [algorithm] will evaluate your self-description.

Your self-description will only be read and evaluated by that person [algorithm], and the person [algorithm] decides about your inclusion into the pool.

For your self-description, please rank the attributes below.

For every attribute, think about where you want to rank it for the qualified **person [algorithm]**.

Attributes that you would like to emphasize more toward the person should be listed higher at the top (with the most important at position 1).

Figure S8: Survey interface for key dependent variable.

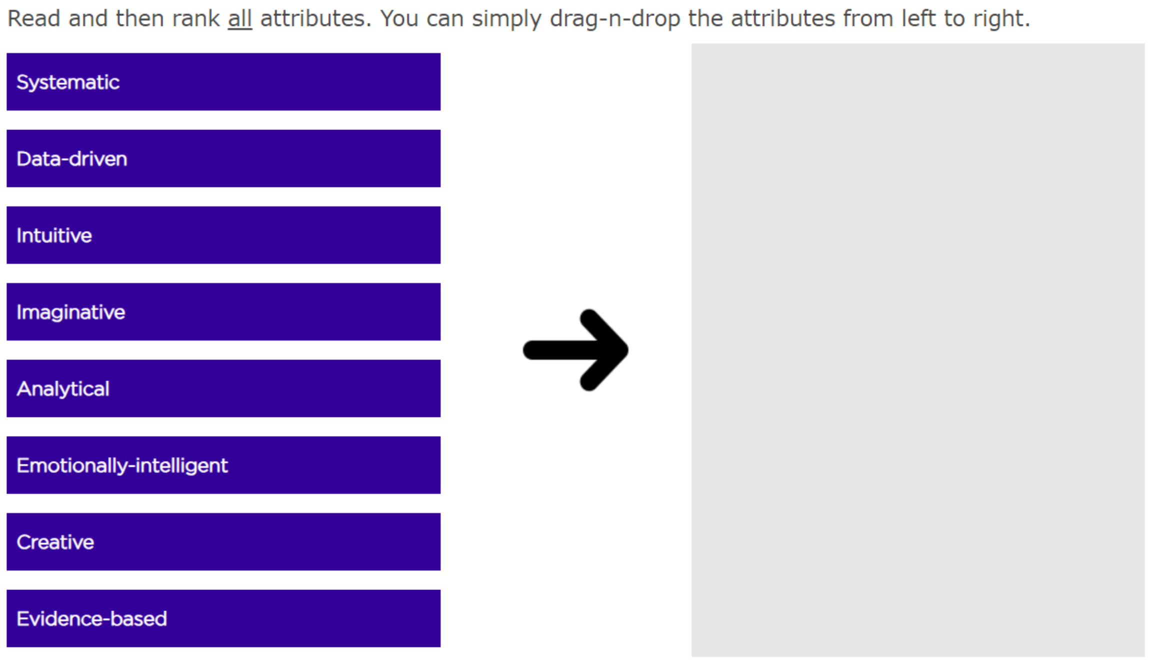

e. **Pretest attributes**

To pretest the attributes included in the self-presentation choice task, we recruited 100 participants from Prolific (M_{age} = 41.6 years; 54% female, 42% male). Every participant answered two questions about a total of 30 attributes. The first question was: “For each attribute below, please indicate the extent to which it signals that someone is an intuitive or rather analytical thinker” (1 = “intuitive” to 7 = “analytical”). The second read: “Please indicate for each attribute below to what extent it makes you look favorable” (1 = “not favorable” to 7 = “very favorable”). By scoring all attributes along the dimensions of intuitive–analytical and favorability, we aimed to prepare two groups of attributes that discriminated along the intuitive–analytical dimension while being on comparable levels on favorability. We tried to control for favorability driven by other factors to be able to isolate a self-presentation effect driven by concerns about the intuitive versus analytical impression made on others.

We followed a selection strategy of choosing the most discriminating items for an intuitive and analytical group of attributes ($t(99) = 16.84$, $P < .001$, $d = 1.68$), whereby neither group was significantly less favorable than the other ($t(99) = -1.53$, $P = .13$ $d = 0.15$). Below is the full set of attributes along with their scores for favorability and intuitive versus analytical signal. The final included intuitive attributes were creative, imaginative, emotionally intelligent, and intuitive. The analytical attributes were data-driven, evidence-based, systematic, and analytical.

Table S63: Dependent samples t-test comparing the intuitive and analytical attribute subgroups on favorability and intuitive–analytical signal.

Dependent variable	Comparison	*df*	*t*	*P*	*d*	95% CI
Favorability	Intuitive–analytical	99	1.53	.130	0.15	[−0.36, 0.03]
Intuitive (1) versus analytical (7) signal	Intuitive–analytical	99	−16.84	< .001	−1.68	[−2.11, −1.35]

Figure S9: Full list of tested attributes with their favorability and intuitive–analytical ratings.

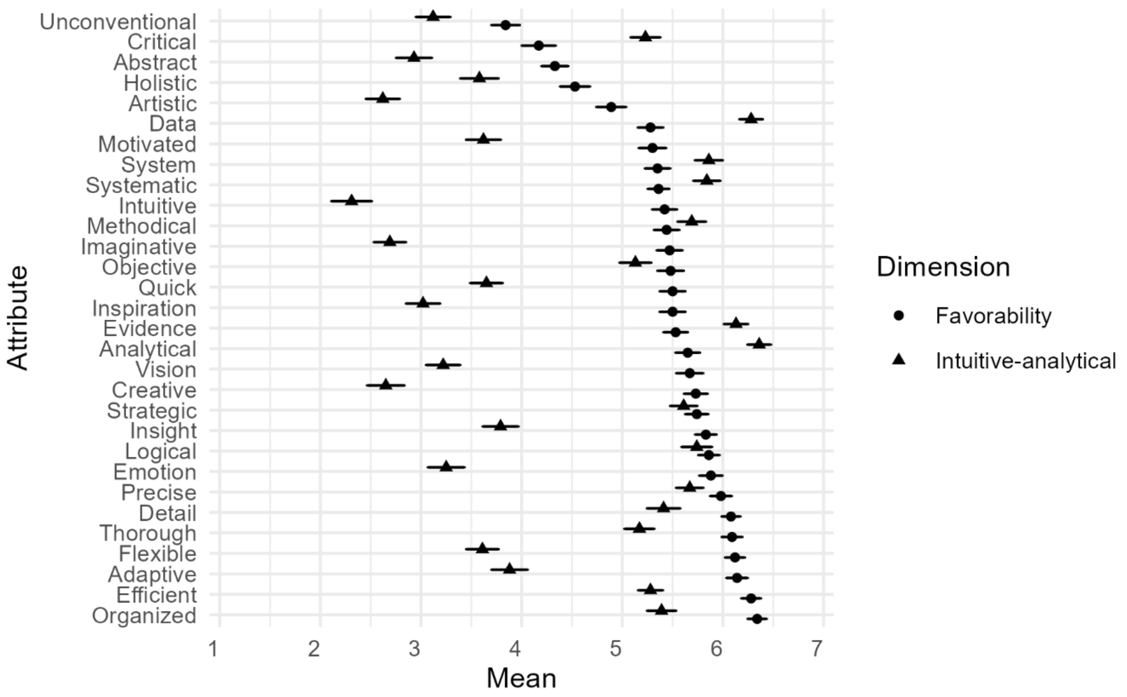

Note: Error bars represent standard error of the mean.

f. **Analytical priority lay belief measures and results**

In your own view, how important are the following attributes of your self-description for the person [algorithm] when evaluating you?

- Emotionally intelligent
- Systematic
- Intuitive
- Analytical

All measured on seven-point Likert scales from 1 = "not important at all" to 7 = "very important."
The measure of analytical priority lay belief demonstrated low internal reliability with a Cronbach's alpha of .48. In hindsight, we reasoned that our measure was mistakenly operationalized and did indeed measure two dimensions. Indeed, a factor analysis using principal axis factoring with varimax rotation. A two-factor solution was extracted and revealed a first factor defined by high loadings on analytical (0.621) and systematic (0.855), whereas the second factor was characterized by emotionally intelligent (0.579) and intuitive (0.747).

We analyzed every item individually, as well as the two-factor solution in light of our hypothesized effect. Only one of the four items showed the expected difference between the algorithm-based and human assessment conditions (see below). We also ran separate mediation models with the items and factors individually specified as the mediator, the condition as the independent variable, and the rank sum of analytical attributes as the dependent variable. Only the model that included "emotionally intelligent" as the mediator resulted in a mediation.

We stick to the pre-registered analysis using an index aggregating all four items. We measured analytical priority lay beliefs with two other operationalizations in Study 1a and Study 3 and our results are robust when using these measures.

Conceptually, we believe this lack of group differences and hence mediation can be traced back to the fact that the scale is rather measuring absolute importance instead of relative importance. The lay belief, however, is conceptualized as prioritizing analytical *relative to* intuitive skills, as measured by the other mediator scales and the rank sum. In other words, we merely assess how important every dimension is perceived to be in isolation in this study and not whether any assessor sees one dimension as more or less important than the other. We suggest that interpreting the results in light of this difference helps us understand the effect. Specifically, both AI and humans are perceived to value analytical skills, as shown by prior research (explaining little differences on these two items), yet AI is perceived to see less value in emotional skills given the lay belief about its emotional incapacities (explaining the difference on this item).

Table S64: Results for independent samples t-tests on mediator index and individual items.

Variable	AI, N = 895[1]	Human, N = 894[1]	P
Mediation index	4.04 (0.99)	3.92 (0.92)	.010
Systematic	4.81 (1.59)	4.83 (1.57)	.79
Analytical	5.63 (1.39)	5.53 (1.40)	.14
Emotionally intelligent (reversed)	2.86 (1.67)	2.53 (1.52)	< .001
Intuitive (reversed)	2.86 (1.54)	2.80 (1.52)	.40

[1]Mean (SD)

Table S65: Regression results for mediation model: Analytical rank sum.

	Analytical rank sum	Analytical priority lay belief	Analytical rank sum
(Intercept)	17.742***	3.981***	17.742***
	(0.128)	(0.023)	(0.097)
Condition	–1.141***	0.117**	–0.707***
	(0.256)	(0.045)	(0.194)
Analytical priority lay belief			–3.720***
			(0.101)
R^2	0.011	0.004	0.436
Adj. R^2	0.010	0.003	0.436
Number obs.	1789	1789	1789

Note: Unstandardized regression coefficients are displayed, with standard errors in parentheses.
*$P < .05$, **$P < .01$, ***$P < .001$
Indirect effect: $b = -0.43$ (0.17), $P = .009$, 95% CI = [−0.77, −0.11]

g. **Key results robustness checks**

Table S66: Results for independent samples t-test on standardized median rank differences.

Dependent variable	Comparison	*df*	*t*	*P*	*d*	95% CI
Standardized median rank difference (analytical - intuitive)	AI–Human	1,788	−4.40	< .001	−0.21	[−0.30, −0.11]

Table S67: Results for ANCOVA on analytical rank sum, including covariates age, gender, occupation, ethnicity, approved Prolific tasks, and skills required for research pool.

Dependent variable	Comparison	*df*	*t*	*P*	*d*	95% CI
Analytical rank sum	AI–human	1,780	−4.74	< .001	−0.21	[−0.31, −0.12]

Table S68: Results for ANCOVA on standardized median rank difference, including covariates age, gender, occupation, ethnicity, approved Prolific tasks, and skills required for research pool.

Dependent variable	Comparison	*df*	*t*	*P*	*d*	95% CI
Standardized median difference (analytical - intuitive)	AI–human	1,780	−4.66	< .001	−0.21	[−0.31, −0.12]

Table S69: Descriptive statistics for aggregate rank of analytical and intuitive attributes and results for Wilcoxon rank sum test between conditions.

Variable	AI, *N* = 895[1]	Human, *N* = 894[1]	*P*
Analytical attributes	4.29, 4, (2.25)	4.58, 5, (2.22)	< .001
Intuitive attributes	4.71, 5, (2.32)	4.42, 4, (2.35)	< .001

[1]Mean, median, (SD)

Figure S10: Mean rank per attribute including 95% CI for the mean and results for independent samples t-test between conditions.

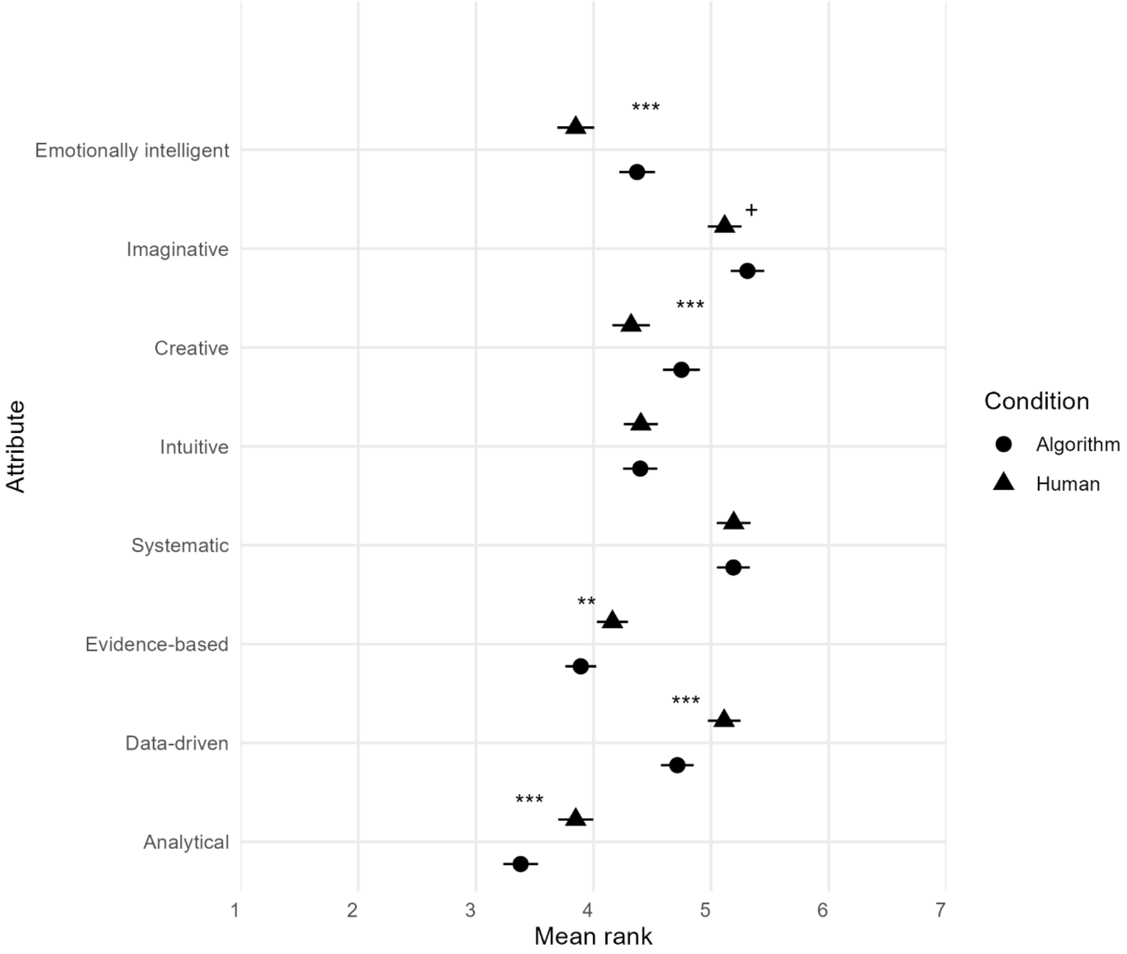

Note: *P < .05, **P < .01, ***P < .001, ^{+}P < .1

h. **Moderation by skills required for research pool**

Table S70: Regression results for moderation models: Condition and skills required for research pool participation.

Dependent variable	Predictor	*df*	*b*	*t*	*P*	95% CI
Analytical rank sum	Condition	1,784	0.11	4.50	< .001	[0.001, 0.02]
	Skills required for research pool	1,784	0.11	4.80	< .001	[0.002, 0.02]
	Interaction	1,784	0.02	0.83	.407	[0.00, 0.00]

Note: Condition: Human = 0, AI = 1

We did not explore a moderating role of self-monitoring tendencies as this variable differed between conditions.

i. **Results when excluding participants with no belief in true incentive alignment**

Table S71: Wilcoxon rank-sum test on analytical rank sum.

Dependent variable	Comparison	$\lvert z\rvert$	*P*	*r*	95% CI
Analytical rank sum	AI–human	4.58	< .001	0.12	[0.07, 0.18]

N = 1,391

j. **Results when excluding participants who correctly guessed the hypothesis**

Table S72: Wilcoxon rank-sum test on analytical rank sum.

Dependent variable	Comparison	$\lvert z\rvert$	*P*	*r*	95% CI
Analytical rank sum	AI–human	4.28	< .001	.10	[0.06, 0.15]

N = 1,708

k. **Key results without exclusions**

Table S73: Wilcoxon rank-sum test on analytical rank sum.

Dependent variable	Comparison	$\lvert z\rvert$	P	*r*	95% CI
Analytical rank sum	AI–human	4.73	< .001	.11	[0.06, 0.16]

N = 1,832

Table S74: Regression results for mediation model: Analytical rank sum.

	Analytical rank sum	Analytical priority lay belief	Analytical rank sum
(Intercept)	17.724***	3.990***	17.724***
	(0.126)	(0.022)	(0.095)
Condition	–1.190***	0.119**	–0.749***
	(0.252)	(0.045)	(0.191)
Analytical priority lay belief			–3.702***
			(0.100)
R^2	0.012	0.004	0.435
Adj. R^2	0.011	0.003	0.435
Number obs.	1832	1832	1832

Note: Unstandardized regression coefficients are displayed, with standard errors in parentheses. **P* < .05, ***P* < .01, ****P* < .001
Indirect effect: *b* = −0.44 (0.17), *P* = .007, 95% CI = [−0.77, −0.12]

I. Assessment protocols

In Study 2, the assessment of participants was conducted to include the best participants in a Prolific pool for other projects, in line with the study instructions. For both the AI and the human condition, the assessment prompt was identical and presented to a research assistant (RA) in the human assessment condition and input to ChatGPT 4o in the AI assessment condition; both were blind to the hypothesis:

> *"The following data originates from a study on participants' self-presentation. They ranked eight attributes from across an analytical-intuitive continuum in an order that best described themselves with the most important one coming first. In this data the first entry per row determines the participant ID, the second entry is the highest ranked item, the third entry the second-highest ranked item and so on. We aim to reach a sample of 100 participants, 50 excelling on the analytical dimension and 50 excelling on the intuitive dimension. Please make a selection of participant IDs based on this request."*

Along with this prompt, we also shared the data containing the ID column and the attribute rankings. Both the RA and AI were free to choose a strategy for determining fitting candidates. Below you can see the applied prompting and output in the AI condition. We included the respective participants in a separate participant list on Prolific and conducted studies with them.

Figure S11: ChatGPT 4o prompting and output screenshots.

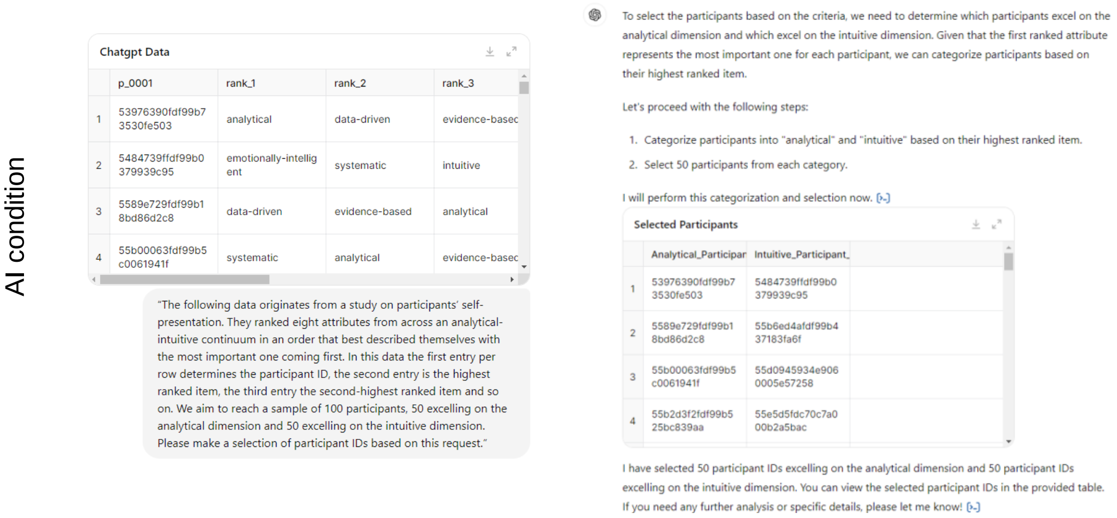

Supplementary Information M: Study 5a

a. Pretest "consider-the-opposite" protocol

To pretest the consider-the-opposite protocol, we recruited 441 participants from Prolific for a three-cell between-subjects design (M_{age} = 38.6 years; 56% female, 43% male). Participants were asked to imagine performing a self-description task similar to the one presented in Study 4. They were informed that their performance would be assessed either by a human or by an algorithm. In the third condition, participants encountered the consider-the-opposite protocol before proceeding. Specifically, they read:

Before continuing to the task, consider the following:

> *Think for a moment about what you expect the algorithm to be capable of and thus what the algorithm focuses on in its assessment. Before you proceed, we would like you to consider the opposite. Can your expectations about what the algorithm assessor is good at when assessing candidates be wrong? Imagine that you were trying to be as unbiased as possible in evaluating this assessor, consider yourself to be in the same role as a judge or juror. Could the algorithm assessor be good at the opposite of what you expect them to be good at? Please write down some reasons for why and how you could be wrong in terms of your expectations about what the algorithm assessor is good at when assessing people.*

Next, participants across conditions indicated their agreement with the following three items measuring the analytical priority lay belief: "The focus of the person [AI] is on analytical attributes rather than intuitive ones," "The person [AI] places an emphasis on analytical attributes rather than intuitive ones," "Analytical attributes take precedence over intuitive ones for the person [AI]" (all measured on seven-point agreement Likert scales; α = .92). Eventually, participants responded to two attention checks and demographics.

b. Measures

See SI-L

c. Descriptives

Table S75: Descriptive statistics for key variables.

Variable	Frequency (%) / mean (SD)
Gender	
Female	1,315.00 (56.66%)
Male	977.00 (42.09%)
Diverse	22.00 (0.95%)
Not disclosed	7.00 (0.30%)
Age (years)	40.02 (13.33)
Analytical rank sum	18.96, 20.00 (5.21)
Education	
Less than high school	18.00 (0.78%)
Master	449.00 (19.35%)
Bachelor	963.00 (41.49%)
Other	186.00 (8.01%)
High school	615.00 (26.50%)
Doctorate	90.00 (3.88%)
Occupation	
Unemployed	248.00 (10.69%)
Retired	120.00 (5.17%)
Self-employed	317.00 (13.66%)
Employed	1,429.00 (61.57%)
Student	130.00 (5.60%)

Variable	Frequency (%) / mean (SD)
Other	77.00 (3.32%)
Approved Prolific tasks	595.65 (828.51)
Ethnicity	
Asian	150.00 (6.46%)
Black	337.00 (14.52%)
No consent	6.00 (0.26%)
Expired data	18.00 (0.78%)
Mixed	150.00 (6.46%)
Other	71.00 (3.06%)
White	1,588.00 (68.42%)
Prefer not to say	1.00 (0.04%)

N = 2,321

Note: Age had two outliers that we removed from the analysis.

Table S76: Descriptive statistics for analytical rank sum by condition.

Variable	AI<br>*N* = 828[1]	AI opposite<br>*N* = 667[1]	Human<br>*N* = 826[1]
Analytical rank sum	18.17, 18.00, 5.28, 10.00, 26.00	19.57, 20.00, 5.14, 10.00, 26.00	19.27, 20.00, 5.10, 10.00, 26.00

[1]Mean, median, SD, range

d. **Key results robustness checks**

Table S77: Results for independent samples t-test on standardized median rank differences.

Dependent variable	Comparison	*df*	*t*	*P*	*d*	95% CI
Standardized median difference (analytical - intuitive)	AI–AI opposite	2,318	−4.76	< .001	−0.25	[−0.35, −0.14]
	AI–Human	2,318	−4.17	< .001	−0.21	[−0.30, −0.11]
	AI opposite–Human	2,318	0.81	.415	0.04	[−0.06, 0.14]

Table S78: Results for ANCOVA on key dependent variable, including covariates age, gender, education, occupation, ethnicity, and approved Prolific tasks.

Dependent variable	Comparison	*df*	*t*	*P*	*d*	95% CI
Analytical rank sum	Human–AI	2,306	−4.46	< .001	−0.27	[−0.37, −0.16]
	Human–AI opposite	2,306	−5.16	< .001	−0.21	[−0.31, −0.12]
	AI–AI opposite	2,306	−0.95	.342	0.06	[−0.05, 0.16]

Table S79: Results for contrasts on standardized median rank differences including covariates age, gender, education, occupation, ethnicity, and approved Prolific tasks.

Dependent variable	Comparison	*df*	*t*	*P*	*d*	95% CI
Standardized median rank difference (analytical - intuitive)	AI–AI opposite	2,312	−4.75	< .001	−0.25	[−0.35, −0.14]
	AI–Human	2,312	−4.28	< .001	−0.21	[−0.30, −0.11]
	AI opposite–Human	2,312	0.71	.480	0.04	[−0.06, 0.14]

Table S80: Descriptive statistics for rank of analytical and intuitive attributes and results for Kruskal-Wallis test between conditions.

Variable	AI<br>*N* = 828[1]	Human<br>*N* = 826[1]	AI opposite<br>*N* = 667[1]	*P*
Analytical attributes	4.54, 5, (2.25)	4.89, 5, (2.17)	4.82, 5, (2.16)	< .001
Intuitive attributes	4.45, 4, (2.33)	4.11, 4, (2.34)	4.18, 4, (2.37)	< .001

[1]Mean, median, (SD)

Table S81: Wilcoxon rank-sum tests between conditions for rank of analytical and intuitive attributes.

Dependent variable	Comparison	$\lvert z \rvert$	*P*	*r*
Analytical attributes	AI–human	4.98	< .001	.06
	AI–AI opposite	6.00	< .001	.08
	Human–AI opposite	1.36	.173	.02
Intuitive attributes	AI–human	4.80	.002	.06
	AI–AI opposite	5.81	< .001	.08
	Human–AI opposite	1.20	.230	.02

e. **Results when excluding those who correctly guessed the hypothesis**

Table S82: Wilcoxon-rank sum test by condition.

Dependent variable	Comparison	$\lvert z \rvert$	*P*	*r*	95% CI
Analytical rank sum	Human–AI	4.46	< .001	0.11	[0.06, 0.16]
	Human–AI opposite	1.04	.300	0.03	[0.001, 0.07]
	AI–AI opposite	5.12	< .001	0.13	[0.08, 0.18]

H(2, n = 2,275) = 31.75, $P < .001$, $\eta^2 = 0.01$, 95% CI = [0.005, 0.02]

f. **Key results without exclusions**

Table S83: Descriptive statistics for analytical rank sum by condition.

Dependent variable	AI<br>*N* = 849[1]	AI opposite<br>*N* = 680[1]	Human<br>*N* = 847[1]
Analytical rank sum	18.19, 19.00, 5.29, 10.00, 26.00	19.56, 20.00, 5.12, 10.00, 26.00	19.30, 20.00, 5.08, 10.00, 26.00

[1]Mean, median, SD, range

Table S84: Wilcoxon-rank sum test by condition.

Dependent variable	Comparison	$\lvert z \rvert$	*P*	*r*	95% CI
Analytical rank sum	Human–AI	4.31	< .001	0.10	[0.06, 0.15]
	Human–AI opposite	1.07	.283	0.03	[0.001, 0.17]
	AI–AI opposite	5.04	< .001	0.13	[0.08, 0.18]

H(2, n = 2,376) = 30.34, $P < .001$, $\eta^2 = 0.01$, 95% CI = [0.004, 0.02]

Figure S12: Mean rank per attribute including 95% CI for the mean and results for ANOVA between conditions.

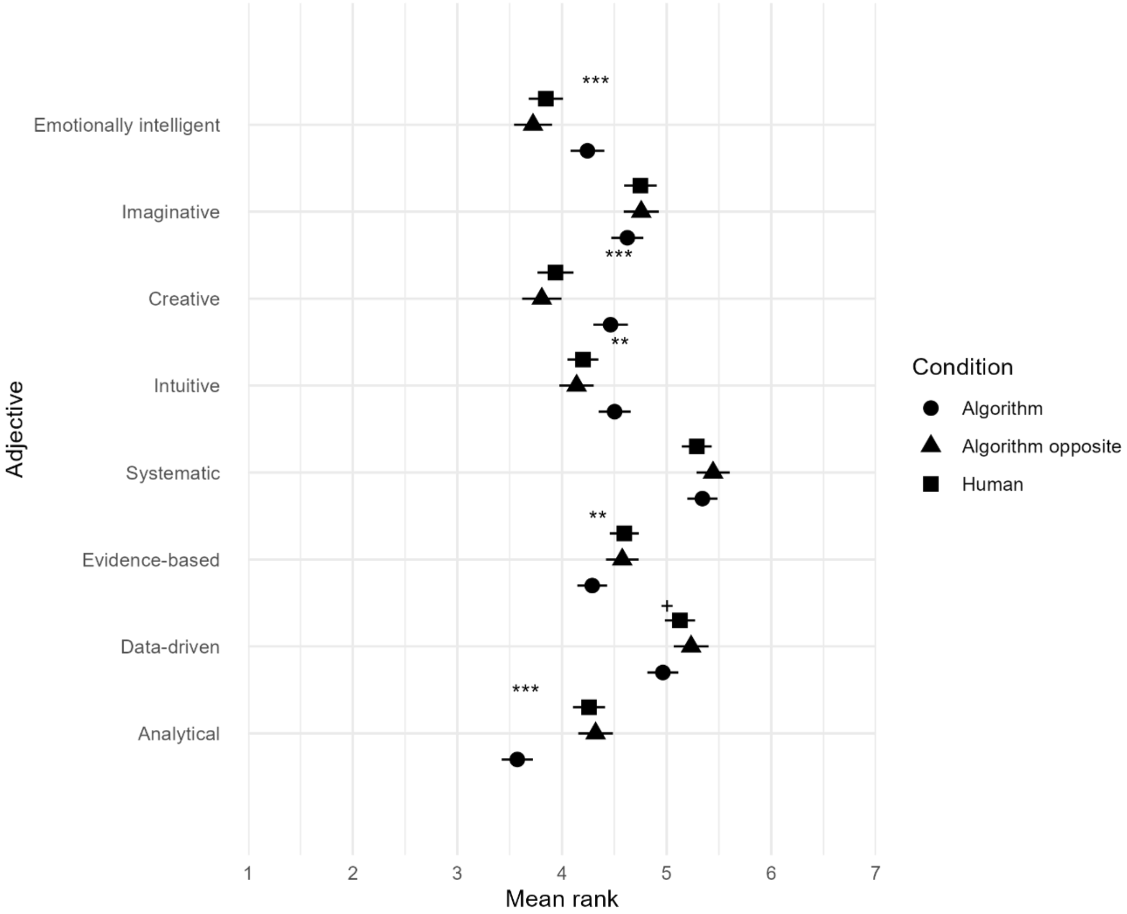

Note: *P < .05, **P < .01, ***P < .001, ^{+}P < .1

g. **Assessment protocols**

In Study 5a, we followed the same assessment protocol from Study 4 but specified that five instead of 50 participants should be selected. We had an RA assess the responses in the human condition and input the data in ChatGPT 4o for both AI conditions (see below).

Figure S13: ChatGPT 4o prompting and output screenshots for the AI and AI opposite condition.

AI condition

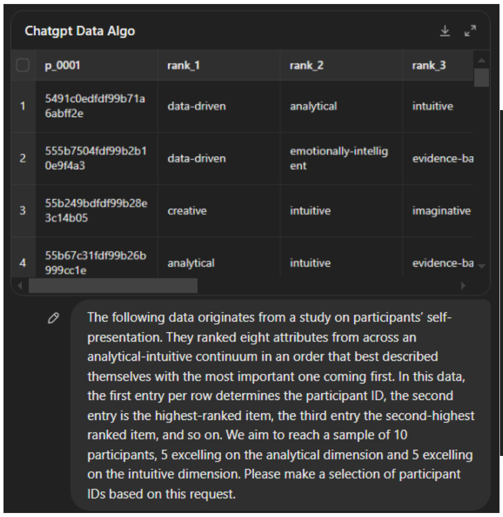

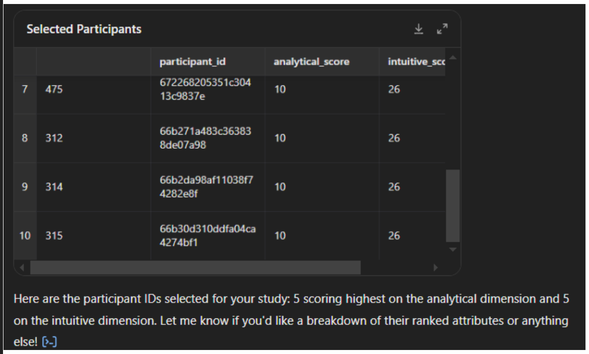

AI opposite condition

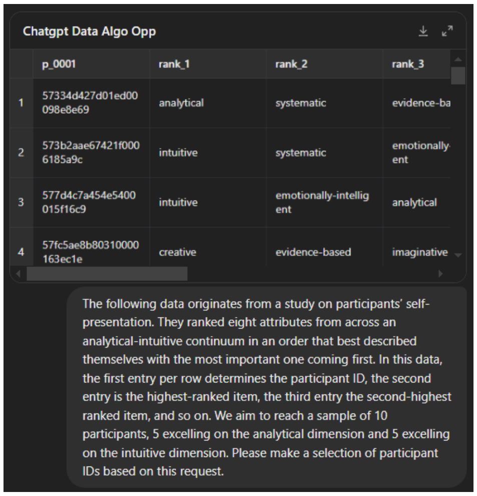

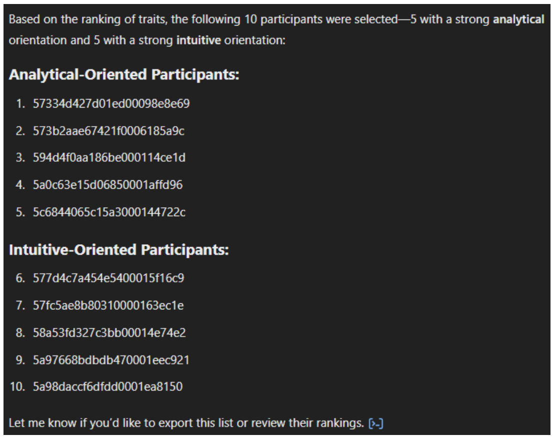

Supplementary Information N: Study 5b

a. **Pretest "consider-the-opposite" protocol**

To pretest the consider-the-opposite protocol, we recruited 442 participants from Prolific for a three-cell between-subjects design (M_{age} = 42.3 years; 54% female, 44% male). Participants were asked to imagine performing a self-description task similar to the one presented in Study 4. They were informed that their performance would be assessed either by a human or by an algorithm. In the third condition, participants encountered the following manipulation:

Before continuing to the task, consider the following:

> *Some people might think that an algorithm is not capable to process emotions and intuition and thus focuses on analytical attributes in assessment ...but this is a misjudgment. For a moment, set aside your expectations about the algorithm. Please write down a reason why the algorithm might be able to focus on emotions and intuitions as well.*

Next, participants across conditions indicated their agreement to the following three items measuring the analytical priority lay belief: "The focus of the person [algorithm] is on analytical attributes rather than intuitive ones," "The person [algorithm] places an emphasis on analytical attributes rather than intuitive ones," "Analytical attributes take precedence over intuitive ones for the person [algorithm]" (all measured on seven-point agreement Likert scales; α = .94). Eventually, participants responded to two attention checks and demographics.

b. **Measures**

See SI-L

c. **Descriptives**

Table S85: Descriptive statistics for key variables.

Variable	Frequency (%) / means, medians (SD)
Gender	
Female	1,561 (65.86%)
Male	744 (31.39%)
Diverse	53 (2.24%)
Not disclosed	12 (0.51%)
Age (years)	37.59 (14.06)
Self-monitoring	4.56 (0.89)
Analytical rank sum	19.51, 20.00 (5.27)
Education	
Less than high school	20.00 (0.84%)
Master	399.00 (16.84%)
Bachelor	957.00 (40.38%)
Other	221.00 (9.32%)
High school	690.00 (29.11%)
Doctorate	83.00 (3.50%)
Occupation	
Unemployed	224.00 (9.45%)
Retired	100.00 (4.22%)
Self-employed	259.00 (10.93%)
Employed	1,490.00 (62.87%)
Student	247.00 (10.42%)
Other	50.00 (2.11%)
Approved Prolific tasks	683.44 (1,073.72)
Unknown	1
Ethnicity	

Variable	Frequency (%) / means, medians (SD)
Asian	310.00 (13.09%)
Black	336.00 (14.18%)
No consent	10.00 (0.42%)
Expired data	34.00 (1.44%)
Mixed	186.00 (7.85%)
Other	104.00 (4.39%)
White	1,389.00 (58.63%)
Unknown	1

N = 2,370

Table S86: Descriptive statistics for analytical rank sum by condition.

Variable	AI<br>*N* = 829[1]	AI intuitive<br>*N* = 716[1]	Human<br>*N* = 825[1]
Analytical rank sum	18.91, 19.00, 5.33, 10.00, 26.00	20.10, 21.00, 5.34, 10.00, 26.00	19.61, 20.00, 5.08, 10.00, 26.00

[1]Mean, median, SD, range

d. **Key results robustness checks**

Table S87: Results for independent samples t-test on standardized median rank differences.

Dependent variable	Comparison	*df*	*t*	*P*	*d*	95% CI
Standardized median difference (analytical - intuitive)	AI–AI intuitive	2,367	−4.35	< .001	−0.22	[−0.32, −0.12]
	AI–Human	2,367	−2.90	.004	−0.14	[−0.24, −0.05]
	AI intuitive –Human	2,367	1.56	.119	0.08	[−0.02, 0.19]

Table S88: Results for ANCOVA on key dependent variable, including covariates age, gender, education, occupation, self-monitoring, ethnicity, and approved Prolific tasks.

Dependent variable	Comparison	*df*	*t*	*P*	*d*	95% CI
Analytical rank sum	Human–AI	2,346	2.82	.005	0.13	[0.04, 0.22]
	Human–AI intuitive	2,346	−2.20	.028	−0.09	[−0.19, −0.01]
	AI–AI intuitive	2,346	−4.91	< .001	−0.23	[−0.33, −0.13]

Table S89: Results for contrasts on standardized median rank differences including covariates age, gender, education, occupation, self-monitoring, ethnicity, and approved Prolific tasks.

Dependent variable	Comparison	*df*	*t*	*P*	*d*	95% CI
Standardized median rank difference (analytical - intuitive)	AI–AI intuitive	2,359	−4.70	< .001	−0.22	[−0.33, −0.13]
	AI–Human	2,359	−2.88	.004	−0.14	[−0.24, −0.05]
	AI intuitive –Human	2,359	1.92	.055	0.08	[−0.02, 0.18]

Table S90: Descriptive statistics for rank of analytical and intuitive attributes and results for Kruskal-Wallis test between conditions.

Variable	AI<br>*N* = 829[1]	Human<br>*N* = 825[1]	AI intuitive<br>*N* = 716[1]	*P*
Analytical attributes	4.73, 5, (2.22)	4.90, 5, (2.19)	5.03, 5, (2.17)	< .001
Intuitive attributes	4.27, 4, (2.34)	4.10, 4, (2.32)	3.97, 4, (2.29)	< .001

[1]Mean, median, (SD)

Table S91: Wilcoxon rank-sum tests between conditions for rank of analytical and intuitive attributes.

Dependent variable	Comparison	|z|	P	r
Analytical attributes	AI–human	3.18	.001	.04
	AI–AI intuitive	5.26	< .001	.07
	Human–AI intuitive	2.21	.027	.03
Intuitive attributes	AI–human	3.05	.002	.04
	AI–AI intuitive	4.99	< .001	.06
	Human–AI intuitive	2.03	.042	.03

e. Results when excluding participants with no belief in true incentive alignment

Table S92: Wilcoxon-rank sum tests for key dependent variable.

Dependent variable	Comparison	|z|	P	r	95% CI
Analytical rank sum	Human–AI	3.12	.002	0.09	[0.03, 0.15]
	Human–AI intuitive	1.66	.10	0.05	[0.002, 0.11]
	AI–AI intuitive	4.15	< .001	0.12	[0.06, 0.18]

N = 1,732

f. Results when excluding those who correctly guessed the hypothesis

Table S93: Wilcoxon-rank sum test by condition.

Dependent variable	Comparison	|z|	P	r	95% CI
Analytical rank sum	Human–AI	2.58	.009	0.06	[0.01, 0.11]
	Human–AI intuitive	2.41	.016	0.06	[0.01, 0.11]
	AI–AI intuitive	4.54	< .001	0.12	[0.06, 0.17]

H(2, n = 2,303) = 21.84, $P < .001$, $\eta^2 = 0.01$, 95% CI = [0.002, 0.02]

g. Key results without exclusions

Table S94: Descriptive statistics for analytical rank sum by condition.

Dependent variable	AI *N* = 854[1]	AI intuitive *N* = 741[1]	Human *N* = 851[1]
Analytical rank sum	18.92, 19.00, 5.31, 10.00, 26.00	20.13, 22.00, 5.32, 10.00, 26.00	19.63, 20.00, 5.06, 10.00, 26.00

[1]Mean, median, SD, range

Table S95: Wilcoxon-rank sum test by condition.

Dependent variable	Comparison	|z|	P	r	95% CI
Analytical rank sum	Human–AI	2.69	.007	0.07	[0.02, 0.12]
	Human–AI intuitive	2.37	.018	0.06	[0.01, 0.11]
	AI–AI intuitive	4.59	< .001	0.12	[0.06, 0.16]

H(2, n = 2,446) = 22.43, $P < .001$, $\eta^2 = 0.01$, 95% CI = [0.002, 0.02]

Figure S14: Mean rank per attribute including 95% CI for the mean and results for ANOVA between conditions.

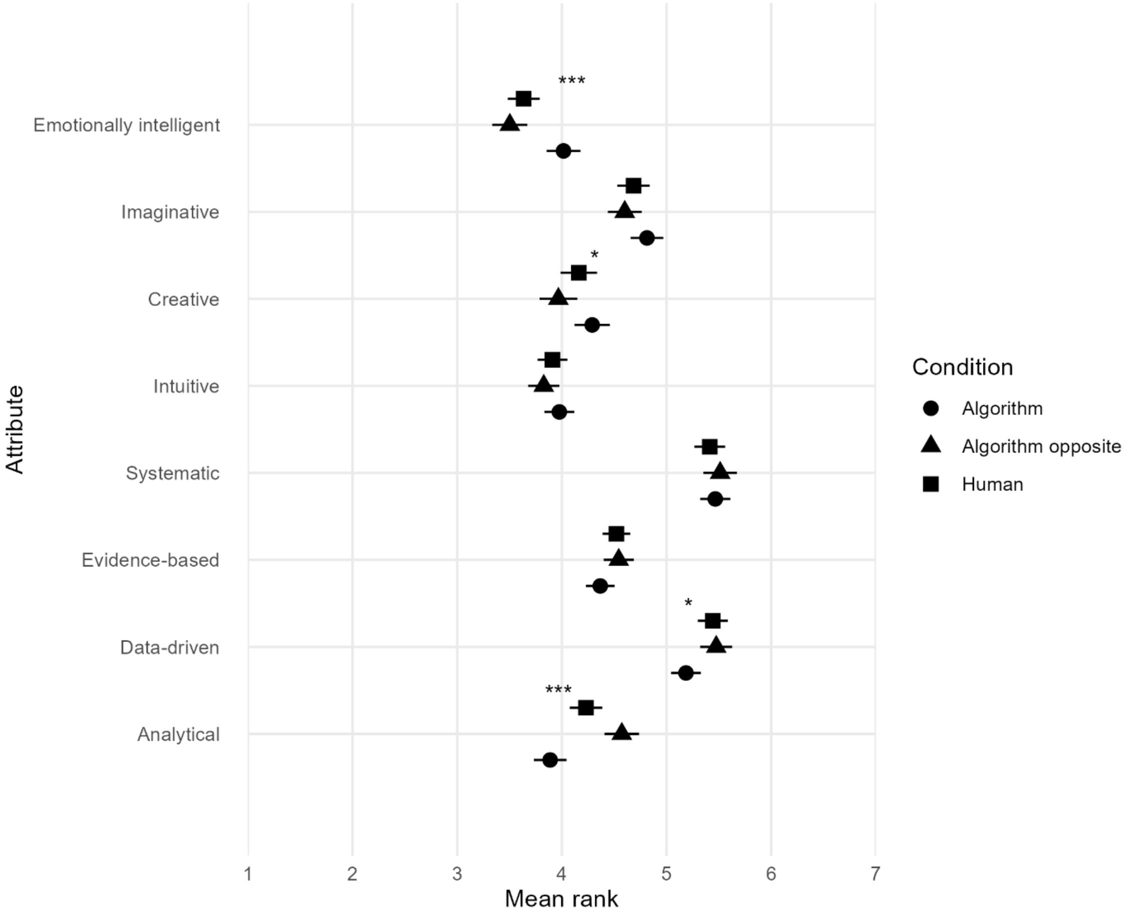

Note: *P < .05, **P < .01, ***P < .001, ^{+}P < .1

h. Assessment protocols

In Study 5b, we followed the identical assessment protocol from Study 4. We had an RA assess the responses in the human condition and input the data in ChatGPT 4o for both AI conditions (see below).

Figure S15: ChatGPT 4o prompting and output screenshots for the AI and AI intuitive conditions.

AI condition

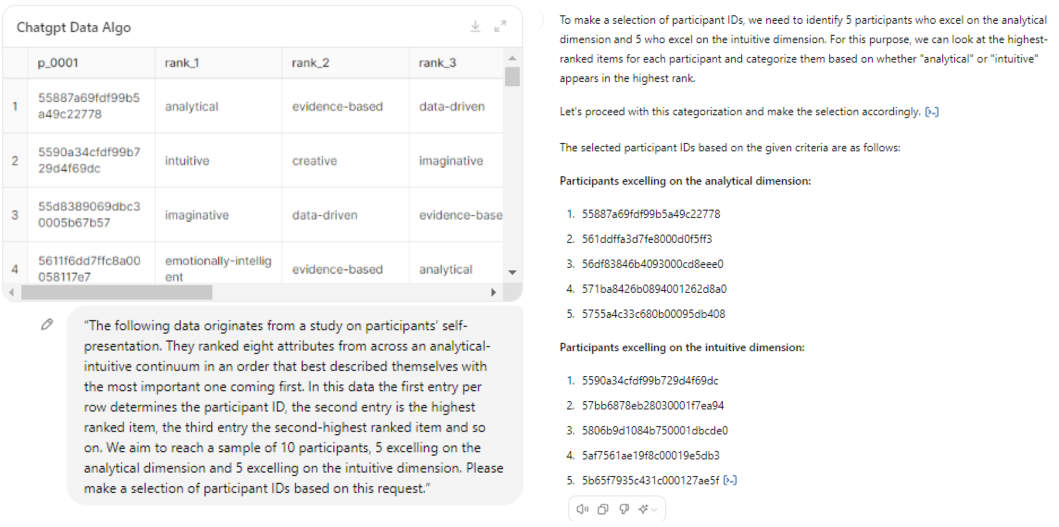

Chatgpt Data Algo

	p_0001	rank_1	rank_2	rank_3
1	55887a69fdf99b5a49c22778	analytical	evidence-based	data-driven
2	5590a34cfdf99b729d4f69dc	intuitive	creative	imaginative
3	55d8389069dbc30005b67b57	imaginative	data-driven	evidence-base
4	5611f6dd7ffc8a00058117e7	emotionally-intelligent	evidence-based	analytical

"The following data originates from a study on participants' self-presentation. They ranked eight attributes from across an analytical-intuitive continuum in an order that best described themselves with the most important one coming first. In this data the first entry per row determines the participant ID, the second entry is the highest ranked item, the third entry the second-highest ranked item and so on. We aim to reach a sample of 10 participants, 5 excelling on the analytical dimension and 5 excelling on the intuitive dimension. Please make a selection of participant IDs based on this request."

To make a selection of participant IDs, we need to identify 5 participants who excel on the analytical dimension and 5 who excel on the intuitive dimension. For this purpose, we can look at the highest-ranked items for each participant and categorize them based on whether "analytical" or "intuitive" appears in the highest rank.

Let's proceed with this categorization and make the selection accordingly.

The selected participant IDs based on the given criteria are as follows:

Participants excelling on the analytical dimension:

1. 55887a69fdf99b5a49c22778
2. 561ddffa3d7fe8000d0f5ff3
3. 56df83846b4093000cd8eee0
4. 571ba8426b0894001262d8a0
5. 5755a4c33c680b00095db408

Participants excelling on the intuitive dimension:

1. 5590a34cfdf99b729d4f69dc
2. 57bb6878eb28030001f7ea94
3. 5806b9d1084b750001dbcde0
4. 5af7561ae19f8c00019e5db3
5. 5b65f7935c431c000127ae5f

AI intuitive condition

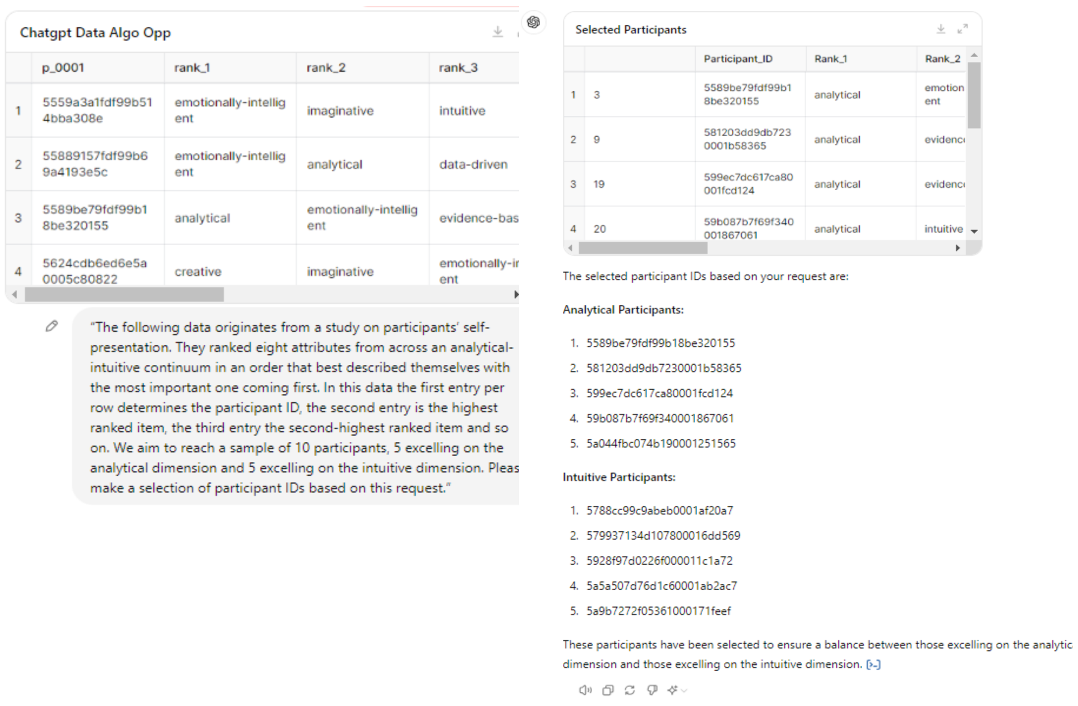

Chatgpt Data Algo Opp

	p_0001	rank_1	rank_2	rank_3
1	5559a3a1fdf99b514bba308e	emotionally-intelligent	imaginative	intuitive
2	55889157fdf99b69a4193e5c	emotionally-intelligent	analytical	data-driven
3	5589be79fdf99b18be320155	analytical	emotionally-intelligent	evidence-bas
4	5624cdb6ed6e5a0005c80822	creative	imaginative	emotionally-ir ent

"The following data originates from a study on participants' self-presentation. They ranked eight attributes from across an analytical-intuitive continuum in an order that best described themselves with the most important one coming first. In this data the first entry per row determines the participant ID, the second entry is the highest ranked item, the third entry the second-highest ranked item and so on. We aim to reach a sample of 10 participants, 5 excelling on the analytical dimension and 5 excelling on the intuitive dimension. Pleas make a selection of participant IDs based on this request."

Selected Participants

		Participant_ID	Rank_1	Rank_2
1	3	5589be79fdf99b18be320155	analytical	emotionent
2	9	581203dd9db7230001b58365	analytical	evidenc
3	19	599ec7dc617ca80001fcd124	analytical	evidenc
4	20	59b087b7f69f340001867061	analytical	intuitive

The selected participant IDs based on your request are:

Analytical Participants:

1. 5589be79fdf99b18be320155
2. 581203dd9db7230001b58365
3. 599ec7dc617ca80001fcd124
4. 59b087b7f69f340001867061
5. 5a044fbc074b190001251565

Intuitive Participants:

1. 5788cc99c9abeb0001af20a7
2. 579937134d107800016dd569
3. 5928f97d0226f000011c1a72
4. 5a5a507d76d1c60001ab2ac7
5. 5a9b7272f05361000171feef

These participants have been selected to ensure a balance between those excelling on the analytic dimension and those excelling on the intuitive dimension.

Supplementary Information O: Study 6

a. **Overview of exemplary disclosure practices**

Table S96: Examples for company disclosure of AI and human assessment.

	Disclosure		Website Link
**Salesforce**	Salesforce Candidate Privacy Statement<br><br>The way we analyze your personal data for the purposes of reviewing your application may involve the use of a tool that compares the candidate profile contained in your application against the profile of the job you applied for. Please note that use of such a tool is only one component of our hiring process and if a decision is made to hire a candidate, a recruiter will always be involved in reviewing the application. Separately, please also note your application may be automatically rejected, without human review, if an open role is filled while you are still in the recruitment process or if you do not meet the minimum job requirements.	Human involvement	Link
**Procter and Gamble**	How does automated decision-making work?<br><br>As part of P&G's recruitment process, we may require candidates to complete online assessments. These assessments measure skills and abilities that generally do not emerge from interviews, and help us to determine whether Candidates are suitably qualified for the roles for which they have applied and whether they exhibit characteristics that would make them successful at P&G.<br><br>P&G complies with applicable laws in conducting its online assessment process, including laws which apply to automated decision-making. To the extent P&G's process may be considered to include automated decision-making, candidates rejected on the basis of their online assessment results are given the opportunity to request a human review.<br><br>This review will consist of a member of P&G's Talent Acquisition team ensuring the computer accurately recorded your selected answers and confirming that you did not receive a passing score on the assessment(s) required for the role for which you applied. If you have additional questions, or would like to request such a human review, please click the button below to initiate the process.	Human review upon request	Link
**Philip Morris International**	Accept privacy notice	Human involvement	Link

	By checking the box, you also agree that the PMI hiring process is supported by an AI-powered system that uses natural language processing to compare PMI's requirements to the job and skills related data (such as job title and skill-based data) set out in the information you provide, including your resume. No automated decision-making is used in the context of our hiring process as the information that you provide will always ultimately be assessed in person by a recruitment agent.		
**Nvidia**	Applicant privacy policy<br><br>How We Use, Store, and Share Recruiting Data<br><br>Research and Development. We use Recruiting Data to develop and improve HR technologies that help us identify and match potential candidates with specific jobs. We may remove personally identifying information from your resume and use it to develop or improve these technologies. All recruiting and hiring decisions are ultimately made by our recruiters and hiring managers.	Human involvement	Link
**Johnson and Johnson**	Careers privacy notice<br><br>Automated Processing<br><br>For certain roles, we use automated tools to assist us in processing and evaluating job applications. These tools leverage complex algorithms and machine-learning technology to assist with the following operations.<br><br>These tools compare the information collected in connection with your application with the specific requirements and qualifications of the target role(s) in order to flag any missing qualifications and assign a ranking to your application. These rankings are then used to prioritize the review of applications and are visible to members of the recruiting team as one of several factors to consider in making decisions about whether to advance a candidate in the recruitment process.<br><br>• **Minimum requirements:** We use automated tools to determine whether, based on the information you have provided to us, you meet the minimum requirements for a position. For example, if the job requires three years of experience as a software developer, these tools will analyze the selections you make in forms you submit through the Careers Sites and/or the words and textual patterns collected in connection with your application to determine whether this type of experience is present or not. In some cases, these automated tools may lead to the rejection of your application without any additional review by a member of our recruitment team.<br><br>• **Job match ranking:** We use automated tools to assign a percentage value to your application that assesses how strong the fit is between your application and the requirements of the target role. Our tools may rely on several factors in assigning these	Human involvement	Link

	percentages, which may vary per job. For example, for certain jobs specific practical experience may be of more relevance than certifications, for others it may be the other way around. Based on the analysis, our automated tools can provide a Johnson & Johnson recruiting employee with an overview of candidates that are potentially qualified for the relevant position. The Johnson & Johnson employee will then assess these recommendations and based on our standard processes, move the candidate forward in the recruitment process.		
**Honeywell**	Applicant privacy notice<br><br>Automated Processing and Profiling<br>Subject to your express consent (which you voluntarily provide when joining our Talent Community or when checking the appropriate consent box during the application process), we conduct 'profiling' to evaluate your suitability for particular job opportunities. This involves using a matching algorithm that is able to evaluate your employment profile and predict your eligibility for a particular job opportunity. We carry out this automated processing for the purposes of:<br>• helping recruiters match candidates' profiles to job opportunities by suggesting candidates for consideration for a specific job opportunity; and<br>• sending personalized job recommendations to you based on your profile and/or preferences or other information that may be of interest for you such as Honeywell news, webinars and events.<br>Please note that based on this profiling, you may not be considered for certain job opportunities unless you have applied for them directly.4<br>We do not make hiring decisions based solely on automated decision-making processes and/or profiling. Your personal information will be subjected to human-made decisions as the ultimate hiring decision will be made by the recruiters or hiring manage	Human involvement	Link
**Chevron**	Applicant privacy notice<br><br>Chevron uses third-party partially automated decision-making tools to assist Chevron in assessing performance, including to support employment decisions.		Link
**Boeing**	Privacy Notice<br><br>General information:<br>Boeing may make certain decisions based solely on automated processing in a variety of ways (including through the use of machine learning and artificial intelligence) to support and improve business operations. Boeing does not, however, subject your Personal Information to automated decision making that produces legal or similarly significant effects.	Human involvement	Link

	Applicants from the US<br>Boeing may deploy certain automated tools to perform specific recruiting tasks that rely on machine learning (also referred to as Automated Intelligence or AI). These tools automatically score you with a rating based on the match of the information provided by you with the job requisition basic and preferred qualifications. Boeing recruiters utilize the screening score as a guide to further evaluate candidates; the score does not automatically result in a decision as to whether the candidate moves forward		
**Amgen**	Job Application Assessment: We use your personal information to assess your qualifications and suitability for the job position you applied for. This includes the use of Artificial Intelligence (AI) tool to assess your application. Any assessment made by the AI tool will be under human supervision and will be validated by our HR department. The legal basis for this processing is Amgen's legitimate interest in evaluating and selecting suitable candidates as well as Amgen's legitimate interest for the establishment, exercise or defense of legal claims.	Human involvement	Link
**3M**	Data Privacy:<br>- Evaluating your information for a position including through use of third parties and artificial intelligence.<br>- Some of these services could be automated, meaning that decisions are made about your application using automated means. Automated results are considered in tandem with, and not in lieu of, human judgement.<br>- In some circumstances, where you have the right under applicable law, you may: (a) have someone review a decision that you think may have been taken about your application using automated means	Human involvement	Link

b. **Stimuli**

Figure S16: Stimuli used in human, AI, as well as AI and human conditions.

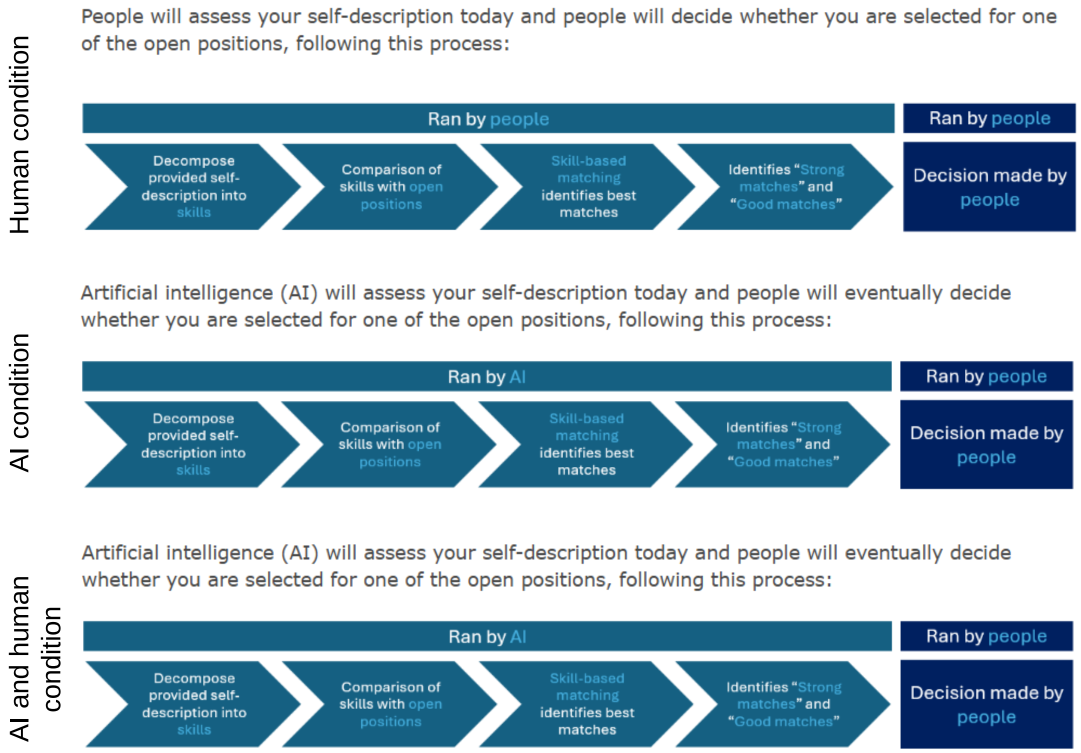

c. **Measures**

See SI-L

d. **Descriptives**

Table S97: Descriptive statistics for key variables.

Variable	Frequency (%) / means, medians (SD)
Gender	
Female	1,338.00 (57.42%)
Male	967.00 (41.50%)
Diverse	16.00 (0.69%)
Not disclosed	9.00 (0.39%)
Age (years)	43.17 (18.34)
Analytical rank sum	18.68, 19.00 (5.24)
Education	
Less than high school	25.00 (1.07%)
Master	445.00 (19.10%)
Bachelor	892.00 (38.28%)
Other	172.00 (7.38%)
High school	696.00 (29.87%)
Doctorate	100.00 (4.29%)
Occupation	
Unemployed	262.00 (11.24%)

Variable	Frequency (%) / means, medians (SD)
Retired	172.00 (7.38%)
Self-employed	268.00 (11.50%)
Employed	1,491.00 (63.99%)
Student	62.00 (2.66%)
Other	75.00 (3.22%)
Approved Prolific tasks	1,293.12 (1,880.99)
Ethnicity	
Asian	118.00 (5.06%)
Black	322.00 (13.82%)
No consent	3.00 (0.13%)
Expired data	10.00 (0.43%)
Mixed	108.00 (4.64%)
Other	60.00 (2.58%)
White	1,709.00 (73.35%)

N = 2,330

Note: Age had one outlier that we removed from the analysis.

Table S98: Descriptive statistics for analytical rank sum by condition.

Dependent variable	AI *N* = 773[1]	AI and human *N* = 768[1]	Human *N* = 789[1]
Analytical rank sum	17.83, 18.00, 5.30, 10.00, 26.00	18.75, 19.00, 5.25, 10.00, 26.00	19.44, 20.00, 5.05, 10.00, 26.00

[1]Mean, Median, SD, Min, Max

e. **Key results robustness checks**

Table S99: Results for independent samples t-test on standardized median rank differences.

Dependent variable	Comparison	*df*	*t*	*P*	*d*	95% CI
Standardized median difference (analytical - intuitive)	AI–AI and human	2,327	−3.30	.001	−0.17	[−0.27, −0.06]
	AI–human	2,327	−5.90	< .001	−0.30	[−0.40, −0.20]
	AI and human–human	2,327	−2.57	.010	−0.13	[−0.23, −0.03]

Table S100: Planned contrasts for key dependent variable, including covariates age, gender, education, occupation, ethnicity, and approved Prolific tasks.

Dependent variable	Comparison	*df*	*t*	*P*	*d*	95% CI
Analytical rank sum	Human–AI	2,316	6.21	< .001	0.31	[0.21, 0.41]
	Human–AI and human	2,316	2.43	.015	0.13	[0.03, 0.23]
	AI–AI and human	2,316	−3.75	< .001	−0.18	[−0.28, −0.07]

Table S101: Results for contrasts on standardized median rank differences including covariates age, gender, education, occupation, ethnicity, and approved Prolific tasks.

Dependent variable	Comparison	*df*	*t*	*P*	*d*	95% CI
Standardized median rank difference (analytical - intuitive)	AI–AI and human	2,321	−3.69	< .001	−0.17	[−0.27, −0.06]
	AI–Human	2,321	−6.09	< .001	−0.30	[−0.40, −0.20]
	AI and human–Human	2,321	−2.37	.018	−0.13	[−0.22, −0.03]

Table S102: Descriptive statistics for rank of analytical and intuitive attributes and results for Kruskal−Wallis test between conditions.

Dependent variable	AI *N* = 773[1]	Human *N* = 789[1]	AI and human *N* = 768[1]	*P*
Analytical attributes	4.46, 4, (2.25)	4.86, 5, (2.17)	4.69, 5, (2.19)	< .001

Dependent variable	AI<br>*N* = 773[1]	Human<br>*N* = 789[1]	AI and human<br>*N* = 768[1]	*P*
Intuitive attributes	4.54, 5, (2.33)	4.14, 4, (2.35)	4.31, 4, (2.37)	< .001

[1]Mean, median, (SD)

Table S103: Wilcoxon rank-sum tests between conditions for rank of analytical and intuitive attributes.

Dependent variable	Comparison	|*z*|	*P*	*r*
Analytical attributes	AI–human	7.08	< .001	.09
	AI–AI and human	4.02	.001	.05
	Human–AI and human	3.09	.002	.04
Intuitive attributes	AI–human	6.80	< .001	.09
	AI–AI and human	3.85	< .001	.05
	Human–AI and human	2.85	.004	.04

f. **Key results without exclusions**

Table S104: Descriptive statistics for analytical rank sum by condition.

Dependent variable	AI<br>*N* = 796[1]	AI and human<br>*N* = 789[1]	Human<br>*N* = 802[1]
Analytical rank sum	17.84, 18.00, 5.30, 10.00, 26.00	18.79, 19.00, 5.26, 10.00, 26.00	19.45, 20.00, 5.04, 10.00, 26.00

[1]Mean, Median, SD, Min, Max

Table S105: Wilcoxon-rank sum tests for key dependent variable.

Dependent variable	Comparison	|*z*|	*P*	*r*	95% CI
Analytical rank sum	Human–AI	6.05	< .001	0.15	[0.10, 0.20]
	Human–AI and human	2.41	.016	0.06	[0.01, 0.11]
	AI–AI and human	3.56	< .001	0.09	[0.04, 0.14]

H(2, n = 2,387) = 36.82, $P < .001$, $\eta^2 = 0.01$, 95% CI = [0.01, 0.03]

Figure S17: Mean rank per attribute including 95% CI for the mean and results for ANOVA between condition.

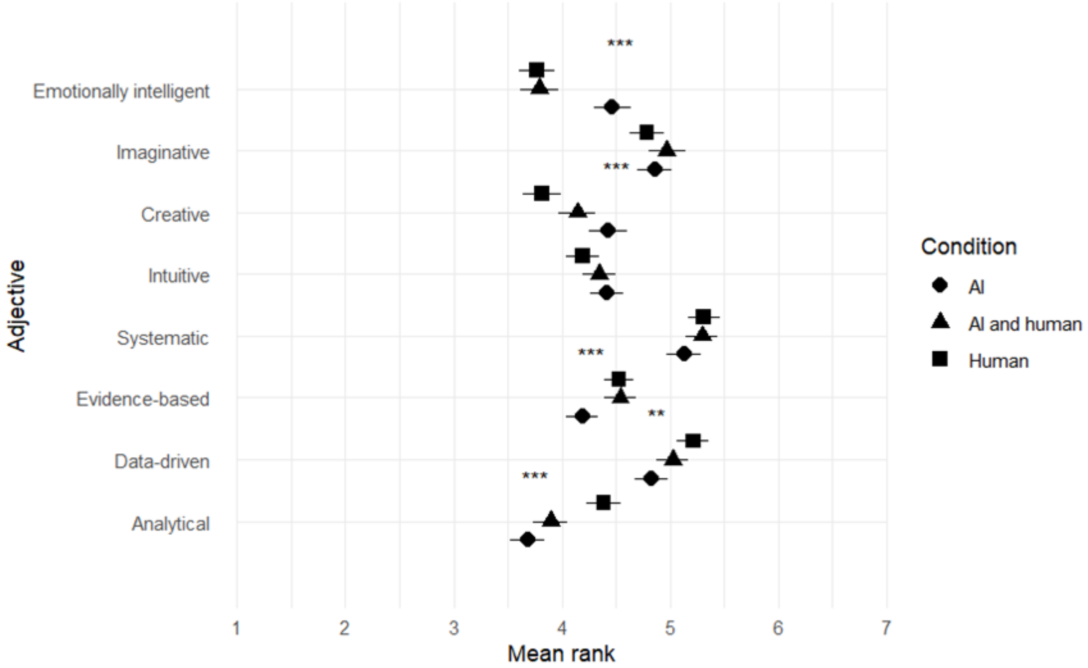

s.

Note: **P* < .05, ***P* < .01, ****P* < .001, +*P* < .1

g. **Assessment protocols**

In Study 6, we followed the identical assessment protocol from Study 4 in the human and AI conditions. We had an RA assess the responses in the human condition and input the data in ChatGPT 4o for the AI condition (see below). In the AI and human condition, we first asked ChatGPT 4o for identification of strong and good matched (as per the manipulation) and then had an RA select candidates.

Figure S18: ChatGPT 4o prompting and output screenshots for the AI and AI and human condition, as well as the instructions given to the RA.

AI condition

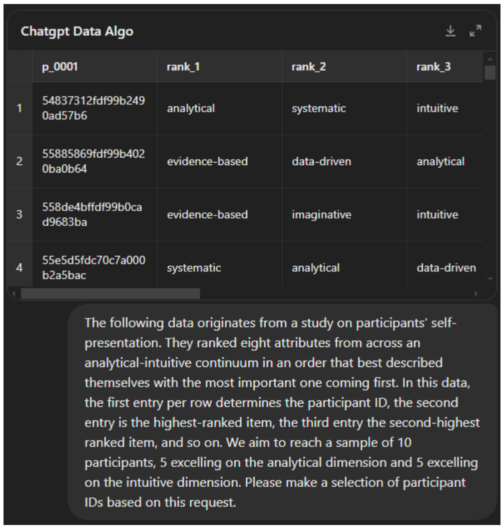

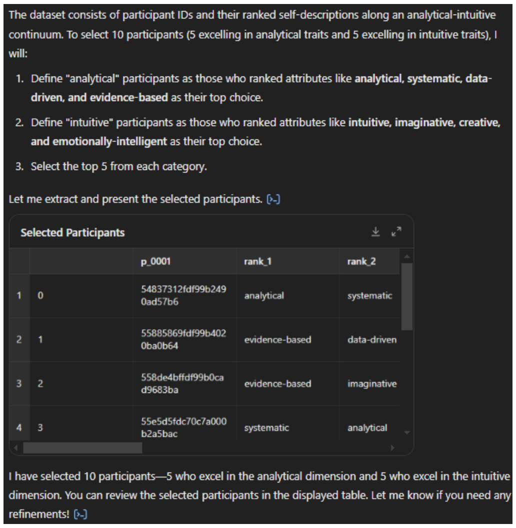

AI and human condition

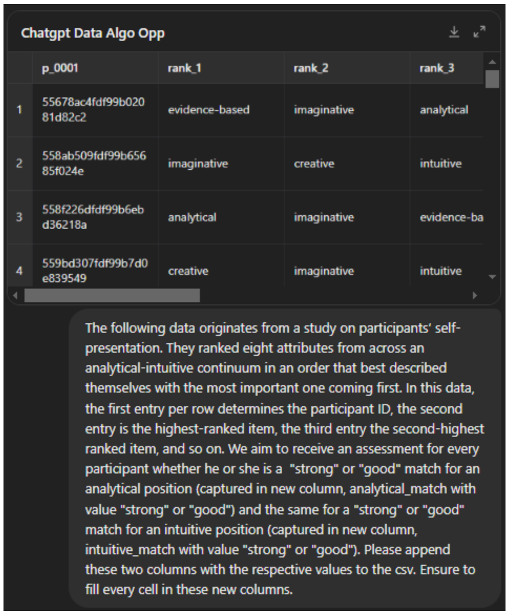

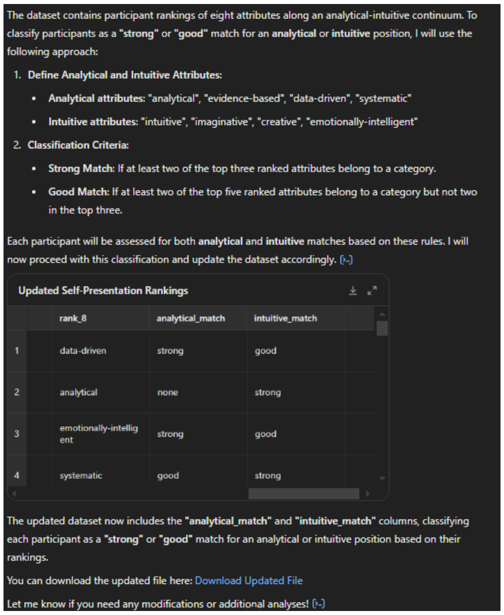

Human condition

The following data originates from a study on participants' self-presentation. They ranked eight attributes from across an analytical-intuitive continuum in an order that best described themselves with the most important one coming first. In this data, the first entry per row determines the participant ID, the second entry is the highest-ranked item, the third entry the second-highest ranked item, and so on. We aim to reach a sample of 10 participants, 5 excelling on the analytical dimension and 5 excelling on the intuitive dimension. Previously, an AI identified how well a participant matches our requirements (last two columns) based on responses. Please make a selection of participant IDs based on this request and the identified matches.

References

1. S. Abdurahman, H. Vu, W. Zou, L. Ungar, S. Bhatia, A deep learning approach to personality assessment: Generalizing across items and expanding the reach of survey-based research. *J. Pers. Soc. Psychol.* **126**, 312–331 (2024).

2. E. D. Campion, *et al.*, Using natural language processing to increase prediction and reduce subgroup differences in personnel selection decisions. *J. Appl. Psychol.* **109**, 307–338 (2024).

3. A. Chalfin, *et al.*, Productivity and selection of human capital with machine learning. *Am. Econ. Rev.* **106**, 124–127 (2016).

4. G. Curto, M. F. Jojoa Acosta, F. Comim, B. Garcia-Zapirain, Are AI systems biased against the poor? A machine learning analysis using Word2Vec and GloVe embeddings. *AI Soc.* **39**, 617–632 (2024).

5. J. Fan, *et al.*, How well can an AI chatbot infer personality? Examining psychometric properties of machine-inferred personality scores. *J. Appl. Psychol.* **108**, 1277–1299 (2023).

6. L. Hickman, *et al.*, Automated video interview personality assessments: Reliability, validity, and generalizability investigations. *J. Appl. Psychol.* **107**, 1323 (2021).

7. A. Lambrecht, C. Tucker, Algorithmic bias? An empirical study of apparent gender-based discrimination in the display of STEM career ads. *Manag. Sci.* **65**, 2966–2981 (2019).

8. J. Liff, N. Mondragon, C. Gardner, C. J. Hartwell, A. Bradshaw, Psychometric properties of automated video interview competency assessments. *J. Appl. Psychol.* **109**, 921–948 (2024).

9. B. Lira, *et al.*, Using artificial intelligence to assess personal qualities in college admissions. *Sci. Adv.* **9**, eadg9405 (2023).

10. S. Zhang, N. Mehta, P. V. Singh, K. Srinivasan, Frontiers: Can an artificial intelligence algorithm mitigate racial economic inequality? An analysis in the context of Airbnb. *Mark. Sci.* **40**, 813–820 (2021).

11. V. Deriu, R. Pozharliev, M. De Angelis, How trust and attachment styles jointly shape job candidates' AI receptivity. *J. Bus. Res.* **179**, 114717 (2024).

12. E. Fumagalli, S. Rezaei, A. Salomons, OK computer: Worker perceptions of algorithmic recruitment. *Res. Policy* **51**, 104420 (2022).

13. M. F. Gonzalez, *et al.*, Allying with AI? Reactions toward human-based, AI/ML-based, and augmented hiring processes. *Comput. Hum. Behav.* **130**, 107179 (2022).

14. F. Kares, C. J. König, R. Bergs, C. Protzel, M. Langer, Trust in hybrid human-automated decision-support. *Int. J. Sel. Assess.* **31**, 388–402 (2023).

15. M. Langer, C. J. König, M. Papathanasiou, Highly automated job interviews: Acceptance under the influence of stakes. *Int. J. Sel. Assess.* **27**, 217–234 (2019).

16. A. Mirowska, L. Mesnet, Preferring the devil you know: Potential applicant reactions to artificial intelligence evaluation of interviews. *Hum. Resour. Manag. J.* **32**, 364–383 (2022).

17. R. Raveendhran, N. J. Fast, Humans judge, algorithms nudge: The psychology of behavior tracking acceptance. *Organ. Behav. Hum. Decis. Process.* **164**, 11–26 (2021).

18. J. Schick, S. Fischer, Dear computer on my desk, which candidate fits best? An assessment of candidates' perception of assessment quality when using AI in personnel selection. *Front. Psychol.* **12**, 739711 (2021).

19. Y. Acikgoz, K. H. Davison, M. Compagnone, M. Laske, Justice perceptions of artificial intelligence in selection. *Int. J. Sel. Assess.* **28**, 399–416 (2020).

20. R. Bedemariam, J. Wessel, The roles of outcome and race on applicant reactions to AI systems. *Comput. Hum. Behav.* **148**, 107869 (2023).

21. A. Bonezzi, M. Ostinelli, Can algorithms legitimize discrimination? *J. Exp. Psychol. Appl.* **27**, 447–459 (2021).

22. Y. E. Bigman, D. Wilson, M. N. Arnestad, A. Waytz, K. Gray, Algorithmic discrimination causes less moral outrage than human discrimination. *J. Exp. Psychol. Gen.* **152**, 4–27 (2022).

23. T. Feldkamp, M. Langer, L. Wies, C. J. König, Justice, trust, and moral judgements when personnel selection is supported by algorithms. *Eur. J. Work Organ. Psychol.* **33**, 130–145 (2024).

24. A. Hilliard, N. Guenole, F. Leutner, Robots are judging me: Perceived fairness of algorithmic recruitment tools. *Front. Psychol.* **13**, 940456 (2022).

25. A. S. Jago, K. Laurin, Assumptions about algorithms' capacity for discrimination. *Pers. Soc. Psychol. Bull.* **48**, 582–595 (2022).

26. J. Ochmann, L. Michels, V. Tiefenbeck, C. Maier, S. Laumer, Perceived algorithmic fairness: An empirical study of transparency and anthropomorphism in algorithmic recruiting. *Inf. Syst. J.* **34**, 384–414 (2024).

27. C. Yan, Q. Chen, X. Zhou, X. Dai, Z. Yang, When the automated fire backfires: The adoption of Algorithm-based HR decision-making could induce consumer's unfavorable ethicality inferences of the company. *J. Bus. Ethics* **190**, 841–859 (2024).

28. S. Tully, C. Longoni, G. Appel, EXPRESS: Lower Artificial Intelligence Literacy Predicts Greater AI Receptivity. *J. Mark.* 00222429251314491 (2025). https://doi.org/10.1177/00222429251314491.

29. J.-P. Stein, T. Messingschlager, T. Gnambs, F. Hutmacher, M. Appel, Attitudes towards AI: measurement and associations with personality. *Sci. Rep.* **14**, 2909 (2024).

30. R. Cadario, C. Longoni, C. K. Morewedge, Understanding, explaining, and utilizing medical artificial intelligence. *Nat. Hum. Behav.* **5**, 1636–1642 (2021).

31. L. Greenhalgh, Z. Rosenblatt, Job Insecurity: Toward Conceptual Clarity. *Acad. Manage. Rev.* **9**, 438 (1984).

32. R. N. Carleton, D. R. McCreary, P. J. Norton, G. J. G. Asmundson, Brief Fear of Negative Evaluation scale—revised. *Depress. Anxiety* **23**, 297–303 (2006).

33. B. Rammstedt, O. P. John, Measuring personality in one minute or less: A 10-item short version of the Big Five Inventory in English and German. *J. Res. Personal.* **41**, 203–212 (2007).
